# Beyond antibiotics: exploring multifaceted approaches to combat bacterial resistance in the modern era: a comprehensive review

**DOI:** 10.3389/fcimb.2025.1493915

**Published:** 2025-03-18

**Authors:** Aref Yarahmadi, Hamide Najafiyan, Mohammad Hasan Yousefi, Elham Khosravi, Ehsan Shabani, Hamed Afkhami, Seyed Soheil Aghaei

**Affiliations:** ^1^ Department of Biology, Khorramabad Branch, Islamic Azad University, Khorramabad, Iran; ^2^ Department of Microbiology and Virology, School of Medicine, Mashhad University of Medical Sciences, Mashhad, Iran; ^3^ Department of Tissue Engineering and Applied Cell Sciences, School of Medicine, Qom University of Medical Sciences, Qom, Iran; ^4^ Cellular and Molecular Research Center, Qom University of Medical Sciences, Qom, Iran; ^5^ Department of Clinical Pharmacy, Faculty of Pharmacy, Tehran University of Medical Sciences, Tehran, Iran; ^6^ Nervous System Stem Cells Research Center, Semnan University of Medical Sciences, Semnan, Iran; ^7^ Department of Medical Microbiology, Faculty of Medicine, Shahed University, Tehran, Iran; ^8^ Department of Microbiology, Qom Branch, Islamic Azad University, Qom, Iran; ^9^ Applied Physiology Research Center, Qom Medical Sciences, Islamic Azad University, Qom, Iran

**Keywords:** antibiotic resistance, bacterial resistance mechanisms, non-antibiotic therapies, alternative treatments, biofilm

## Abstract

Antibiotics represent one of the most significant medical breakthroughs of the twentieth century, playing a critical role in combating bacterial infections. However, the rapid emergence of antibiotic resistance has become a major global health crisis, significantly complicating treatment protocols. This paper provides a narrative review of the current state of antibiotic resistance, synthesizing findings from primary research and comprehensive review articles to examine the various mechanisms bacteria employ to counteract antibiotics. One of the primary sources of antibiotic resistance is the improper use of antibiotics in the livestock industry. The emergence of drug-resistant microorganisms from human activities and industrial livestock production has presented significant environmental and public health concerns. Today, resistant nosocomial infections occur following long-term hospitalization of patients, causing the death of many people, so there is an urgent need for alternative treatments. In response to this crisis, non-antibiotic therapeutic strategies have been proposed, including bacteriophages, probiotics, postbiotics, synbiotics, fecal microbiota transplantation (FMT), nanoparticles (NPs), antimicrobial peptides (AMPs), antibodies, traditional medicines, and the toxin-antitoxin (TA) system. While these approaches offer innovative solutions for addressing bacterial infections and preserving the efficacy of antimicrobial therapies, challenges such as safety, cost-effectiveness, regulatory hurdles, and large-scale implementation remain. This review examines the potential and limitations of these strategies, offering a balanced perspective on their role in managing bacterial infections and mitigating the broader impact of antibiotic resistance.

## Graphical Abstract

Non-antibiotic therapies targeting bacterial infections.

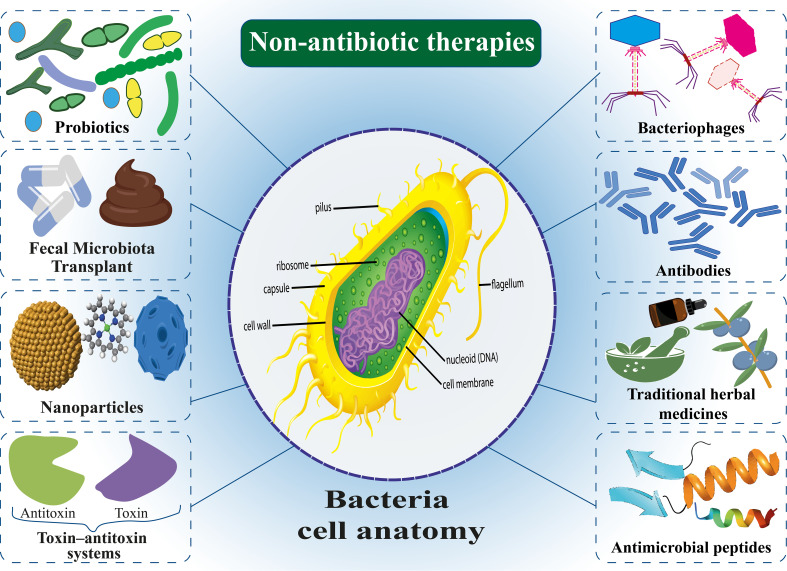

## 1 Introduction

Since the discovery of penicillin in 1929, pathogens responsible for various diseases have been effectively targeted through the widespread use of this antibiotic, its subsequent modifications, and the identification of bioactive scaffolds derived from natural sources. These advancements have propelled the field of antibiotics into a golden era. Antibiotics have made it possible to cure potentially lethal bacterial infections from medical procedures (Konwar et al., 2022). However, antibiotic resistance has led to a concerning increase in drug-resistant bacterial populations, posing a significant threat to global health. In 2008, the Infectious Diseases Society of America (IDSA) acknowledged the growing prevalence of antibiotic resistance in the United States and worldwide (Control, C.F.D, 2002). According to projections from the World Health Organization, antibiotic-resistant infections currently claim approximately 700,000 lives globally each year. By 2050, this number could rise to 10 million deaths, making antibiotic-resistant diseases a more significant cause of mortality than cancer (O’Neill, 2016). In both the United States and globally, *Staphylococcus aureus* is a major contributor to mortality, causing severe infections such as skin and soft tissue infections, inflammatory diseases, and joint and bone infections, as well as complications in individuals with emphysema and AIDS (Klevens et al., 2007; Alder et al., 2020; Del Giudice, 2020; Chen et al., 2022). The treatment of multidrug-resistant (MDR) and extensively drug-resistant (XDR) bacterial infections presents a considerable challenge for clinicians, often resulting in limited therapeutic options and increased morbidity and mortality (Basak et al., 2016; Silva et al., 2018).

To address this crisis, a significant approach that has been proposed is to focus on non-antibiotic alternatives for controlling bacterial infections. These techniques serve as adequate substitutes for antibiotics, possessing comparable capabilities to manage bacterial infections without contributing to antibiotic resistance (Opal, 2016; Lagadinou et al., 2020). Many strategies have been used to treat antibiotic-resistant infections, including using bacteriophages, probiotics, nanoparticles (NPs), antimicrobial peptides (AMPs), antibiofilms, and traditional natural remedies. Extensive research has been conducted on each of these alternatives. While some methods demonstrate considerable effectiveness in laboratory settings, their implementation in clinical practice necessitates further research and effort (Barbosa et al., 2017). Some of these alternative methods have been used before, but antibiotics have been phased out, such as bacteriophages and natural remedies, which have been put back on the agenda due to antibiotic resistance, although these methods may also present some challenges (Golkar et al., 2014). For instance, bacteriophages themselves can cause resistance to phage in bacteria, or natural remedies are more bacteriostatic and must be prescribed with antibiotics for effectiveness (Khaledi et al., 2022). Some natural remedies may cause several side effects due to the complexity and properties of the ingredients. NPs require unique design based on the physicochemical properties of the material (Baker et al., 2020).

This review aims to evaluate the current state of antibiotic resistance and explore non-antibiotic therapies as viable alternatives. Specifically, the objective is to examine the mechanisms by which bacteria develop resistance, assess the role of antibiotic use in livestock as a significant contributor to resistance, and evaluate the potential of non-antibiotic therapies in mitigating antibiotic resistance and enhancing public health outcomes.

## 2 Review methodology

This review used a structured search strategy to identify relevant literature on antibiotic resistance and non-antibiotic therapies. Searches were performed in major academic databases, including PubMed, Scopus, and Web of Science, utilizing a combination of keywords such as “antibiotic resistance,” “alternative therapies,” “bacteriophages,” “probiotics,” “fecal microbiota transplantation,” “antimicrobial peptides,” “antibodies,” “nanoparticles,” “traditional medicines,” and “toxin-antitoxin systems.” Articles published from 1958 to 2025 were included to capture foundational studies and recent advancements in the field. The inclusion criteria focused on studies that addressed bacterial resistance mechanisms, evaluated non-antibiotic therapeutic strategies, and presented experimental or clinical data. Excluded articles were those deemed irrelevant, non-English publications, or duplicate studies across databases. Data extraction involved identifying key findings related to the mechanisms of resistance, therapeutic efficacy, and clinical applicability of the therapies. The results were synthesized to provide a comprehensive analysis of both emerging and established alternatives to antibiotics, highlighting historical perspectives alongside contemporary research.

## 3 The current state of antibiotic resistance

**Figure 1:**
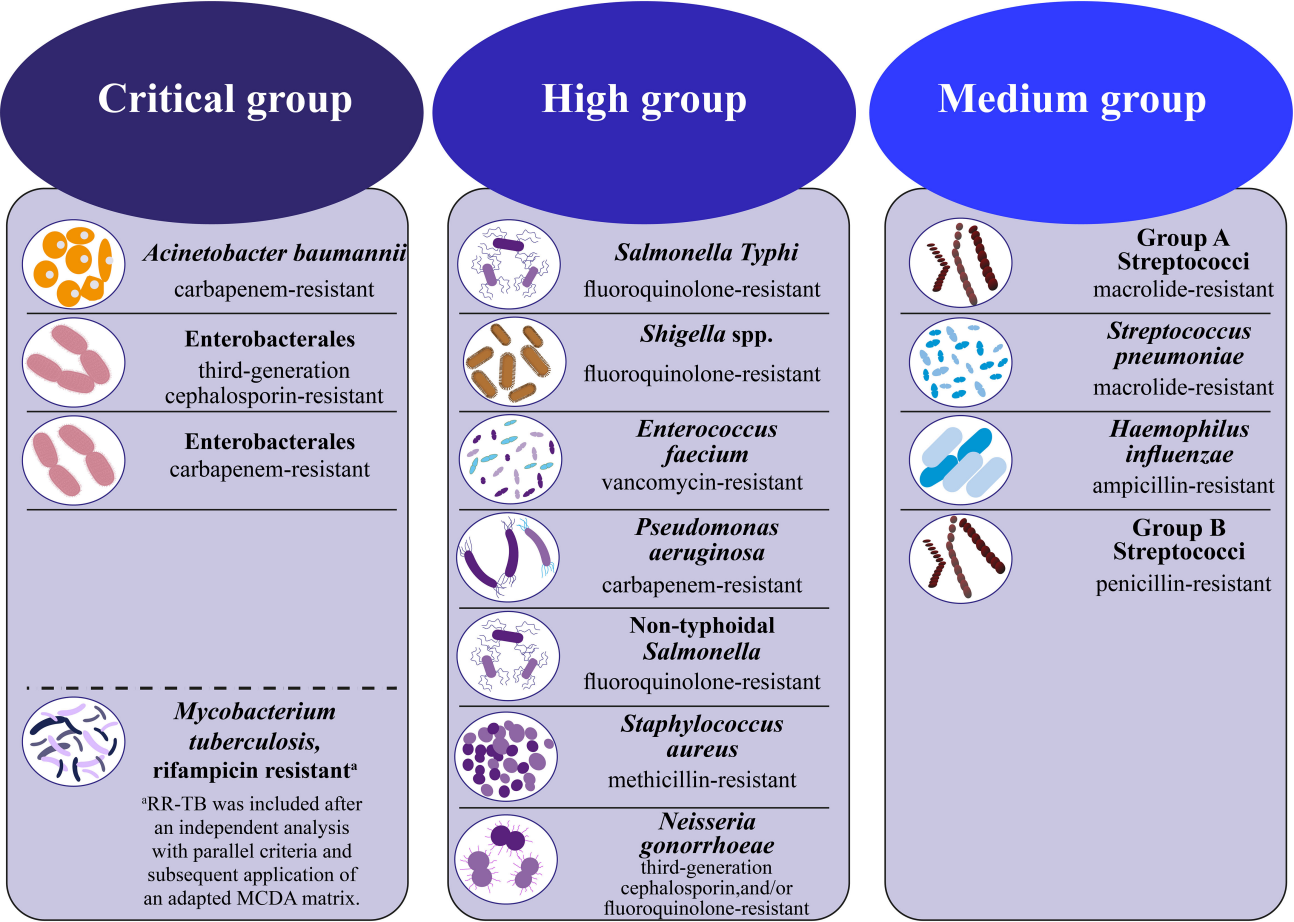
WHO bacterial priority pathogens list, 2024 (Organization, W.H, 2024).

Recently, resistance to routinely used antibiotics has risen, leading to the formation of MDR bacteria that can withstand the effects of last-resort medicines like tigecycline and colistin. The WHO has recently published a roster of significant pathogens that demonstrate elevated levels of resistance to antibiotics. This list is categorized into three priority tiers according to the urgent need for novel antibiotic treatments, namely: critical, high, and medium^1,^ ^2^ (**Figure 1**). Also, the WHO concluded in a study on the worldwide lack of new antimicrobials released in April 2021 that the clinically licensed medicines and existing antibiotic development pipelines are insufficient to treat germs resistant to drugs (Lin et al., 2017; Mancuso et al., 2021; Hou et al., 2023). The report examines the clinically developed antibiotics against the diseases listed in the bacterial priority pathogens list from February 2017. In their analysis, which looked at 27 non-traditional antibacterial agents including bacteriophages and antibodies, the WHO first included non-traditional antibacterial medications (Konwar et al., 2022). Furthermore, the Indian Council of Medical Research (ICMR) illustrated current resistance developments on the priority infections in its annual report on the antimicrobial resistance (Kamruzzaman et al., 2021) research and surveillance network (from January 2020 to December 2020) (Konwar et al., 2022). Based on data gathered from research laboratories and tertiary hospitals, the report emphasized the declining sensitivities of bacteria to frequently used antibiotics for drug-resistant diseases such as β-lactam—β-lactamase inhibitors, cephalosporins, monobactams, and carbapenems. The resistance trend among isolates from secondary bacterial infections in Coronavirus disease (COVID-19)-positive individuals was also investigated, given that India was affected by the COVID-19 pandemic throughout the reporting period. According to these findings, the most often isolated pathogen from the respiratory tract of COVID-19-positive individuals was *Klebsiella pneumoniae* (*K. pneumoniae*), which was followed by *Acinetobacter baumannii* (*A. baumannii*) and *Escherichia coli* (*E. coli*). Furthermore, it has been shown that there was a significant rise in antibiotic resistance of these bacteria when they were isolated from individuals who tested positive for COVID-19. Additionally, the Centers for Disease Control and Prevention (CDC) recently reported that 35,000 people in the US die from infections caused by antibiotic-resistant bacteria, which affect an estimated 2.8 million people annually. In addition, $4.6 billion was needed to address infections caused by six types of bacteria resistant to multiple drugs. The CDC has classified 18 strains of antibiotic-resistant bacteria and fungi into three distinct categories according to the degree of threat they pose to human health, namely urgent, serious, and concerning (Walia et al., 2019; Martins and Rabinowitz, 2020; Sato et al., 2021; Bhardwaj et al., 2022; Konwar et al., 2022). Given these circumstances, the creation of innovative treatments for bacterial infections brought on by pathogens resistant to several drugs is desperately needed (Giurazza et al., 2021).

## 4 Mechanisms of antibiotic resistance in bacteria

**Figure 2:**
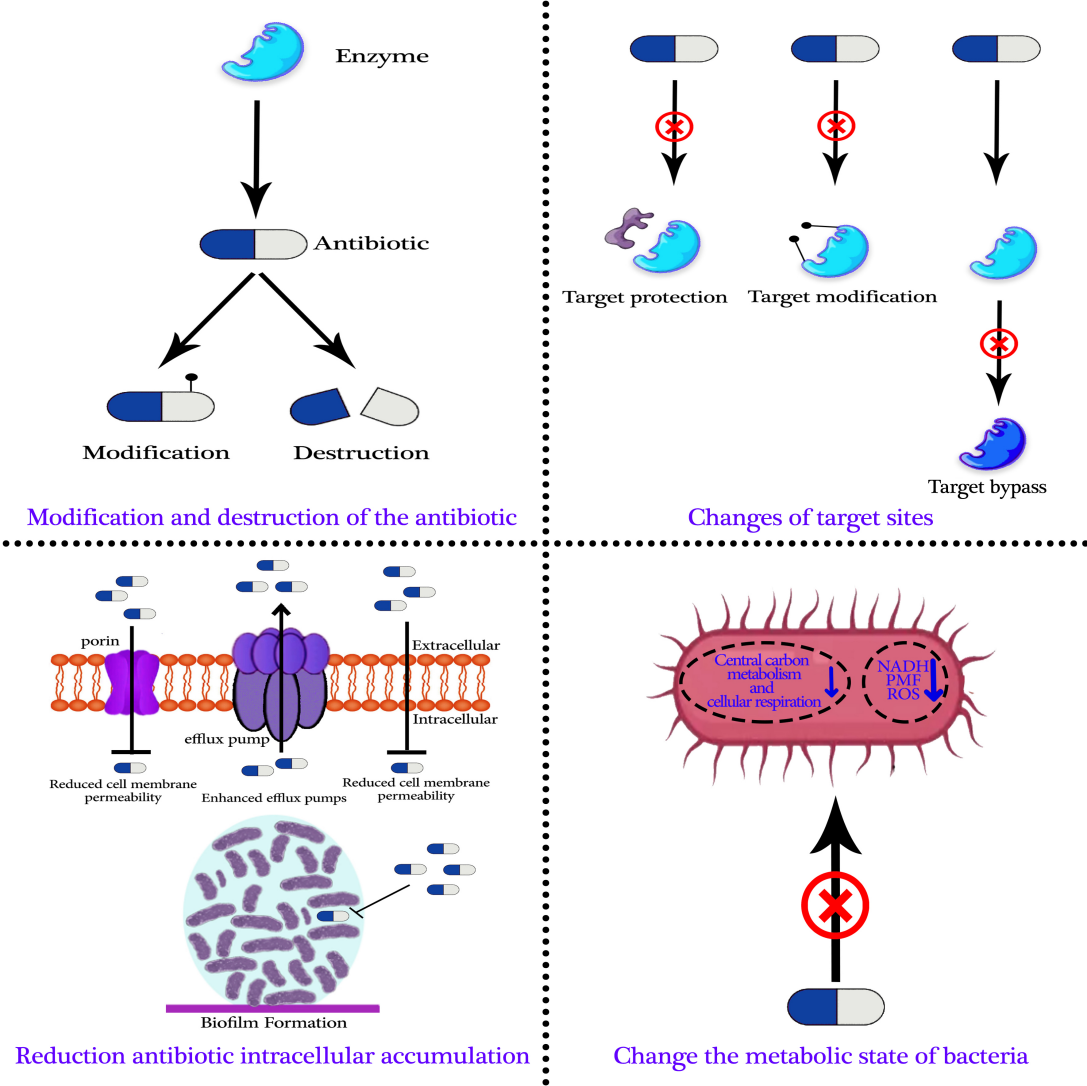
The primary mechanisms of bacterial resistance to antibiotics.

Research has demonstrated that bacterial evolution is the actual cause of antibiotic-resistant microorganisms (Huo et al., 2022; Grant et al., 2023). Researchers discovered antibiotic-resistance genes in bacteria even before the discovery of antibiotics (D’Costa et al., 2006; Pawlowski et al., 2016). Antibiotic-resistant bacteria can now develop extrachromosomal mobile elements or chromosomes as resistance mechanisms. Numerous processes can lead to the development of antibiotic resistance, such as the antibiotic’s modification and destruction, target site modifications, a decrease in the intracellular accumulation of antibiotics, and modifications to the metabolic state of bacteria. Knowing the basis and secret to developing novel strategies to prevent or reverse bacterial resistance is comprehending the process of bacterial resistance (**Figure 2**) (Davies and Bennett, 2017; Mancuso et al., 2021; Mutuku et al., 2022; Xiao et al., 2023).

### 4.1 Modification and destruction of the antibiotic

The synthesis of particular enzymes by bacteria to modify and inactivate antibiotics represents a primary strategy employed by these microorganisms to counteract the effects of antibiotics, thereby rendering them ineffective. This phenomenon is also a significant mechanism contributing to the development of antibiotic resistance (De Pascale and Wright, 2010). Various antibiotic classes, including β-lactams, carbapenems, fluoroquinolones, aminoglycosides, tetracyclines, and macrolides, can be broken down or altered by a broad range of resistance enzymes (Egorov et al., 2018).

β-lactamase is a significant enzyme associated with drug resistance, frequently synthesized by Gram-negative bacteria. This enzyme is categorized into two main types: serine-β-lactamase and metal-β-lactamase. β-lactamases exert their effect on β-lactam antibiotics by hydrolyzing the β-lactam ring structure, thereby altering the conformation of the antibiotic molecule (Tooke et al., 2019). One of the most essential β-lactamases available today is extended-spectrum beta-lactamases (ESBLs), which can both hydrolyze and confer resistance in bacteria to a wide range of β-lactam antibiotics, including cefotaxime, ceftazidime, and aztreonam. Metal-β-lactamase exhibits a broad substrate range, effectively hydrolyzing all β-lactam antibiotics except monocyclic β-lactam antibiotics. The enzymatic activity of metal-β-lactamase is contingent upon the presence of metal ions, specifically Zn, and is not susceptible to inhibition by currently available lactase inhibitors (Mojica et al., 2022; Arer and Kar, 2023; Yang et al., 2023). Furthermore, it is challenging to overcome its mediated antibiotic resistance due to its varied structure and mode of action. New Delhi metalloproteinase-1 (NDM-1) is a recently identified metalloenzyme that can inactivate carbapenems and other β-lactam antibiotics, including penicillin. This enzymatic activity contributes to a significant antibiotic resistance in bacteria, rendering them resistant to a wide array of antibiotics, such as β-lactams, aminoglycosides, carbapenems, macrolides, and quinolones. Notably, these bacteria exhibit sensitivity solely to tigecycline and polymyxin (Kumarasamy et al., 2010; Johnson and Woodford, 2013; Wang et al., 2021). Consequently, bacteria that produce NDM-1 are commonly referred to as super bacteria. Furthermore, the NDM-1 gene is situated on bacterial plasmids, facilitating its horizontal transfer among microorganisms. This characteristic contributes to the widespread prevalence of NDM-1 and complicates efforts for prevention and control (Khan et al., 2017). It is concerning since co-carrying different extended-spectrum β-lactamases, and carbapenemases might result in resistance to practically all β-lactam antibiotics (Potter et al., 2016; Pitout et al., 2019).

Apart from the direct degradation of antibiotics, another significant pathway for drug resistance in bacteria is the alteration of antibiotics (Wright, 2005). Aminoglycoside antibiotics are a typical example, as they include easily modifiable amino groups and several exposed hydroxyl groups (Azucena and Mobashery, 2001; Ramirez and Tolmasky, 2010). The diminished affinity of modified aminoglycoside antibiotics for ribosomal targets reduces their antibacterial efficacy and facilitates the development of bacterial resistance. Notable enzymes that modify aminoglycosides include acetyltransferases, phosphorylases, nucleosidases, and adenylyltransferases (Wright, 1999; Ramirez and Tolmasky, 2010). The genes that encode the enzymes that alter aminoglycosides are often located on plasmids and transposons. However, they can also be found on chromosomes (Azucena and Mobashery, 2001). In a recent study, Bordeleau et al. identified a novel aminoglycoside-modifying enzyme known as APMA, which functions as an acetyltransferase and can inactivate apramycin (Bordeleau et al., 2021). The association of aminoglycoside-modifying enzymes with ESBLs is concerning, as it contributes to the development of MDR. Additionally, various antibiotic-modifying enzymes have been characterized for their roles in conferring resistance to a broad spectrum of antibiotics, including aminoglycosides, rifamycins, macrolides, lincosamides, streptogramins, and phenicols (Darby et al., 2023). For instance, the *lnu* gene encodes a nucleotidyltransferase that modifies lincomycin by adding phosphate-containing groups to the antibiotic, reducing its efficacy (Lüthje et al., 2007). Furthermore, erythromycin esterase and macrolide 2′-phosphotransferase, enzymes produced by members of the Enterobacteriaceae family, disrupt the activity of macrolide antibiotics. These enzymes inhibit the binding of macrolides to the 50S ribosomal subunit by degrading the tetracyclic macrolide’s lipophilic ring structure. This enzymatic activity plays a critical role in developing bacterial resistance to macrolide antibiotics (Boumghar-Bourtchai et al., 2008).

### 4.2 Changes in target sites

An additional mechanism by which bacteria acquire antibiotic resistance involves alterations to the target site, thereby facilitating the development of resistance (Lambert, 2005; Schaenzer and Wright, 2020). Bacteria employ various mechanisms to render antibiotics ineffective by preventing their binding to the target site, thereby contributing to drug resistance. These mechanisms include mutations in the gene that encodes the target, enzymatic modification of the target, and the development of alternative pathways that circumvent the target altogether (Darby et al., 2023; Smith et al., 2023). A solitary point mutation within the *ropB* gene of *E. coli*, which is responsible for encoding RNA polymerase, can confer significant resistance to rifampicin (Jin and Gross, 1988; Floss and Yu, 2005). Resistance to fluoroquinolones arises from mutations in the genes that encode DNA gyrase and topoisomerase IV, both of which are critical for DNA replication. The simultaneous presence of multiple mutations is generally associated with an increased likelihood of developing a significant level of drug resistance. For instance, four specific mutations in the penicillin-binding protein PBP5 are frequently observed in *Enterococcus faecium* strains exhibiting substantial drug resistance. However, the presence of a mutation at any single site does not independently confer a high level of drug resistance; instead, it is the concurrent occurrence of mutations at all four sites that results in a pronounced degree of drug resistance (Kebriaei et al., 2020; An et al., 2023; Hunashal et al., 2023).

Furthermore, less antibiotic binding results from changing the antibiotic’s action target. Macrolide antibiotics inhibit bacterial protein synthesis by reversibly binding to the peptidyl-tRNA binding site of the 50S ribosomal subunit. This binding obstructs the translocation process of newly synthesized peptidyl-tRNA molecules from the acceptor site to the donor site. Ribosomal methylation modifications facilitated by *erm* genes represent the primary mechanism through which bacteria inhibit the efficacy of macrolide antibiotics. Notable examples of common erythromycin resistance methyltransferases (ERMs) include *erm*(A) and *erm*(C) found in *Staphylococcus* species, as well as *erm*(B) present in *Enterococcus* and *Pneumococcus* (Weisblum, 1995; Leclercq, 2002). Currently, the most extensively researched colistin resistance gene, *mcr*, encodes a phosphoethanolamine transferase. This enzyme facilitates the addition of phosphoethanolamine to lipid A, which diminishes the negative charge of lipopolysaccharide (LPS). This modification subsequently reduces the binding affinity of colistin, thereby enabling bacterial resistance to this antibiotic (Liu et al., 2016). Furthermore, chloramphenicol-florfenicol resistance (*CFR*) is attributed to a methyltransferase encoded by the *CFR* gene on a plasmid. This enzyme methylates explicitly the adenine residue at position A2503 in the 23S rRNA, thereby conferring resistance to linezolid in bacterial populations (Toh et al., . 2007; Schwarz et al., 2000).

Furthermore, target bypass represents a strategic approach designed to render the original target obsolete by creating alternative pathways that circumvent the effects of antibiotics. The interaction of methicillin with its target, PBP2a, inhibits the synthesis of the bacterial cell wall, ultimately leading to bacterial cell death. The protein PBP2a, encoded by the *mecA* gene in *S. aureus*, is a substitute for the original target of methicillin. This replacement protein does not impede cell wall synthesis when methicillin binds to it, resulting in the emergence of methicillin-resistant *S. aureus* (MRSA) (Stapleton and Taylor, 2002; Larsen et al., 2022). In *E. coli*, the formation of the cell wall through peptidoglycan cross-linking is primarily facilitated by penicillin-binding proteins (PBPs). These proteins, which are the main targets of β-lactam antibiotics, catalyze D, D-transpeptidase activity. In a recent study, Caveney et al. identified an alternative cross-linking mechanism mediated by the L, D-transpeptidase YcbB, which can bypass the D, D-transpeptidase activity associated with PBPs. This mechanism contributes to the development of bacterial resistance to β-lactam antibiotics (Caveney et al., 2019).

### 4.3 Decrease in intracellular accumulation of antibiotics

Certain antibiotics affect targets located within the cell or the cell membrane, necessitating their passage through these barriers to achieve therapeutic action. Consequently, some bacteria have developed strategies to minimize contact with antibiotic targets, including the formation of biofilms, decreased permeability of the cell membrane, and the upregulation of efflux pumps. The distinct composition of the outer membrane in Gram-negative bacteria establishes a natural permeability barrier. Consequently, the permeability of antibiotics in Gram-negative bacteria is reduced compared to that in Gram-positive bacteria, particularly concerning lipophilic antibiotics. Gram-negative bacteria possess specialized proteins on their outer membrane, known as porins, which facilitate the transport of certain hydrophilic substances and nutrients. Notable examples include OmpC and OmpF in *E. coli*, ompD in *Salmonella*, OmpK35 and OmpK36 in *K. pneumoniae*, and OprD in *Pseudomonas aeruginosa* (*P. aeruginosa*) (Hasdemir et al., 2004; Tsai et al., 2011; Fernández and Hancock, 2012). Bacteria develop resistance to these antibiotics by decreasing the expression of specific proteins, thereby diminishing bacterial permeability. Furthermore, the association between the loss or down-regulation of porin expression and the emergence of drug resistance is intricate, frequently occurring in conjunction with the expression of efflux pumps (Fernández and Hancock, 2012; Prajapati et al., 2021).

In addition to inhibiting the entry of antibiotics into the cell, the efflux of intracellular antibiotics represents a significant mechanism contributing to bacterial resistance. The efflux pumps located on the bacterial cell membrane are critical components in the mechanism of bacterial drug resistance, as they actively transport antibiotics out of the cell (Webber and Piddock, 2003; Fernández and Hancock, 2012). Antimicrobial efflux pumps in bacteria are classified into five primary superfamilies: 1) ATP-binding cassette transporters (ABC family); 2) major facilitator superfamily (MFS family); 3) resistant-nodulation-division families (RND family); 4) small MDR families (SMR family); and 5) multidrug and toxic compound extrusion families (MATE) (Henderson et al., 2021; Si et al., 2023). The RND family of efflux pumps is among the most thoroughly investigated, owing to the diverse array of substrates present in nearly all Gram-negative bacterial strains (Symmons et al., 2009). The active efflux pump in Gram-negative bacteria comprises three distinct components: an outer membrane protein, a membrane fusion protein, and an efflux protein or transporter within the inner membrane. The RND efflux pump AcrAB-TolC, consisting of the periplasmic fusion protein AcrA, the plasma membrane transporter AcrB, and the outer membrane channel protein TolC, is a critical component in the mechanism of MDR observed in *E. coli* (Jang and Tolc, 2023). Before exerting its effects on intracellular target sites, the drug initially associates with the plasma membrane transporter AcrB, subsequently being extruded from the cell via AcrA and the outer membrane channel TolC. AcrB exhibits minimal substrate specificity, allowing a wide range of structurally diverse compounds to function as substrates. This characteristic contributes to its role in MDR. Significantly, AcrAB-TolC is pivotal in developing acquired drug resistance associated with resistant plasmids, as it enables and regulates the dissemination of genes that encode MDR efflux pumps (Jang and Tolc, 2023).

The overexpression of a drug efflux pump constitutes a significant mechanism underlying MDR in *A. baumannii*. AdeABC is the inaugural and most extensively researched RND efflux pump identified in *A. baumannii*. AdeABC is implicated in aminoglycoside resistance and exhibits a specific efflux mechanism for tetracyclines and quinolones. Furthermore, AdeABC can interact with carbapenemases or outer membrane proteins to facilitate resistance to carbapenems. Furthermore, efflux pumps significantly contribute to the antibiotic resistance observed in *P. aeruginosa*. For instance, the MexE-MexF-OprN efflux system is implicated in expulsing carbapenems, chloramphenicol, and fluoroquinolone antibiotics (Poole and Srikumar, 2001; Aeschlimann and Therapy, 2003).

Furthermore, bacterial biofilms serve as a natural impediment that limits the penetration of antibiotics into bacterial cells. The development of bacterial biofilms not only functions as a physical barrier but also facilitates the activity of certain enzymes, such as β-lactamase, which degrade antibiotics, consequently enhancing bacterial resistance. Furthermore, certain positively charged substances that contribute to biofilm formation may create a charge barrier that impedes the efficacy of certain positively charged antibiotics, including aminoglycosides (Høiby et al., 2010; Grande et al., 2020; Singh et al., 2022).

### 4.4 Change the metabolic state of bacteria

A substantial body of evidence indicates a strong correlation between bacterial metabolic processes and the efficacy of antibiotics (Schrader et al., 2020). Bactericidal antibiotic therapy disrupts cellular homeostasis, increasing ATP requirements and an elevated metabolic burden. Consequently, this disruption gradually results in the accumulation of toxic metabolic by-products, ultimately culminating in cellular death (Bhargava and Collins, 2015; Yang et al., 2019). Bacteria exhibiting reduced metabolic activity demonstrate resistance or tolerance to various classes of antibiotics, while heightened drug sensitivity correlates with increased metabolic activity (Bhargava and Collins, 2015; Stokes et al., 2019). Lopatkin et al (Lopatkin et al., 2021). identified a correlation between genes involved in central carbon and energy metabolism and antibiotic resistance. The metabolic changes observed lead to a reduction in basal respiration, inhibiting the activation of antibiotic-induced tricarboxylic acid cycle (TCA cycle) activity. This mechanism allows for the evasion of metabolic toxicity and the reduction of drug lethality (Lopatkin et al., 2021). Genetic modifications that enhance the basal respiration rate of *E. coli* have been shown to improve the efficacy of bactericidal antibiotics when applied to wild-type bacterial cells (Lobritz et al., 2015). Furthermore, quiescent or auxotrophic bacterial strains have the potential to exhibit resistance to a diverse range of antibiotics. Research indicates that under conditions of nutrient deprivation, *E. coli* demonstrates an increased synthesis of guanosine tetraphosphate (ppGpp). This accumulation of ppGpp interferes with the biosynthesis of peptidoglycans and phospholipids, ultimately contributing to developing bacterial resistance to penicillin antibiotics (Amato et al., 2013). The advancement of metabolomics has established a valuable methodology for investigating the metabolic profiles of drug-resistant bacterial strains. Metabolomic investigations of MDR bacteria have revealed that alterations in glucose and amino acid metabolism can disrupt the central metabolic pathway known as the TCA cycle. This disruption may influence electron transfer within the respiratory chain, subsequently impacting bacterial sensitivity to antibiotics and contributing to the development of bacterial tolerance or drug resistance (Vincent et al., 2016; Zampieri et al., 2017). Peng et al (Peng et al., 2015). conducted a comparative analysis of the metabolomics of kanamycin-resistant *Edwardes fluminata* and its sensitive counterpart, *E. dwardes*. Their findings revealed that the MDR bacteria exhibited deficiencies in central metabolic pathways, particularly in glucose and amino acid metabolism. These results are consistent with earlier research on resistant strains of *P. aeruginosa* and *Stenotrophomonas maltophilia* (Alonso et al., 2004; Stickland et al., 2010). Moreover, the exogenous injection of glucose and alanine, in conjunction with antibiotics, may restore the kanamycin sensitivity of resistant bacteria. This highlights the intimate relationship between a bacterium’s metabolic state and antibiotic resistance. In conclusion, the physiological metabolism of bacteria plays a significant role in determining their sensitivity to antibiotics; however, this metabolic process is highly intricate and influenced by numerous factors. Consequently, further research is essential to elucidate the mechanisms underlying the relationship between bacterial physiological metabolism and antibiotic sensitivity (Tenover, 2006; Peng et al., 2015; Keller and Dörr, 2023; Xiao et al., 2023).

## 5 One of the primary sources of antibiotic resistance: livestock

Antibiotics have a significant impact on animals’ and humans’ health, thus even though they are beneficial, overuse of them can have adverse effects, most notably the emergence of resistant bacteria (Page and Gautier, 2012; Chantziaras et al., 2014; Simoneit et al., 2015). To prevent infections and make up for unsanitary conditions on commercial livestock farms, antibiotics are administered to many food-producing animals, particularly chickens, pigs, and cattle (Sivaraman and Yann, 2018). To mitigate this impact, some industrialized nations have implemented regulations to curb the overuse of antibiotics as a preventative measure or as a tool for animal acceleration (Muaz et al., 2018). Over the last 30 years, several monitoring systems have been put in place to examine the use of antibiotics in animal breeding. The Danish Integrated Antimicrobial Resistance Monitoring and Research Program was the first to be introduced in 1995 (Hammerum et al., 2007). Many research initiatives have been created in this field recently; one such initiative is the Disseminating Innovative Solutions for Antibiotic Resistance Management project (DISARM), which involves nine European nations (Ghimpeteanu et al., 2022). Livestock can introduce resistant germs into the environment through various means, such as contaminated biowaste, meat, or milk or direct contact with the animals and people who work on animal farms. In a similar vein, human diseases receive antimicrobial-resistant genes from zoonotic pathogens. As animals and animal-derived products are transported globally, the spread of AMR affecting the food supply in one country can present risks in other regions. For instance, following the administration of avoparcin, a glycopeptide antibiotic commonly utilized as a growth promoter for animals in Europe, the dissemination of vancomycin-resistant *Enterococci* (Ferry et al., 2020) was observed in animal feed and meat (Wegener et al., 1999). As a result, the European Union (EU) outlawed the use of avoparcin in food-producing animals, and a decline in the prevalence of VRE in humans and animals has been seen (Bates, 1997). Likewise, the use of fluoroquinolones (e.g., enrofloxacin) in milch and meat animals is primarily responsible for the rise of ciprofloxacin-resistant *Salmonella*, *E. coli*, and *Campylobacter*, which have resulted in difficult-to-treat illnesses in humans. Drug-resistant bacteria have been known to spread worldwide through food commerce and travel on several occasions. When modest dosages of antibiotics are given to bacteria over extended periods, they become resistant to them. It’s standard procedure to give livestock low-dose antibiotics to encourage weight increase. Antibiotics are also haphazardly administered to flocks or herds that are overcrowded in an attempt to prevent sickness. Antibiotic resistance arises and spreads as a result of such behaviors. These actions cause an enormous buildup of antibiotics in the environment and for bacteria that come into touch with them to develop resistance (Allen and , 2014). Several nations, notably the EU, have outlawed using antibiotics in feed or as growth hormones (Heuer et al., 2011). Hence, the exclusion of antibiotics from animal feed has been offset by enhancements in the efficiency of swine and poultry production facilitated by incorporating various feed supplements. These additives include organic and inorganic acids, immunoglobulins, omega-3, probiotics, β-glucans derived from yeast, essential oils, prebiotics, zinc oxide, cysteine, threonine, as well as a variety of herbs and spices (Adewole et al., 2016; Bucław, 2016). However, the results of another study showed that the removal of preventative in-feed antibiotics results in a little decline in animal health and productivity (Diana et al., 2019). Apart from the development of drug resistance resulting from genetic changes, the most likely sources of antibiotic resistance among microorganisms originating from non-clinical contexts (Martinez et al., 2008). As a result, research is concentrated on comprehending the molecular mechanisms and origins of resistance acquisition. To demonstrate that animals serve as reservoirs for resistant genes, it is challenging to pinpoint the specific pathway and amount of resistance components that animals pass on to humans. The environment’s naturally occurring resistance genes add to the complexity of this. For instance, resistance to natural antibiotics is known to develop far before agriculture advanced. The metallo-β-lactamases, exemplified by NDM, have an ancient evolutionary origin to the extent that there is no discernible sequence similarity between various classes of these genes (Hall et al., 2004; Bebrone, 2007; Ma et al., 2021).

## 6 Non-antibiotic therapies

### 6.1 Bacteriophages

Bacteriophages, commonly called phages, are viruses that selectively infect and eliminate bacteria without harming human cells (Kasman and Porter, 2022; Teymouri et al., 2024). Phages in medical applications can be traced back to ancient times when river water was used to treat conditions like leprosy (Keen, 2012). Ernest Hankin, an Italian bacteriologist, stated in 1896 that there was an unknown substance in the Ganges River that could limit the cholera epidemic. Later, in 1904, Frederick Twort hypothesized that this substance was an unknown virus, and in 1910, Félix d’Hérelle independently observed and identified bacteriophages. A landmark event occurred when a patient fully recovered from an infection within 24 hours of receiving phage therapy (Setlow et al., 1992; Harper, 2021). This breakthrough led to the widespread production of phages for various pathogens at the Institut Pasteur in France, continuing until 1974. Documented evidence of phage therapy persisted until 1979; however, the advent of antibiotics, which provided a simpler and more convenient method for treating bacterial infections, resulted in the decline of phage-based treatments (Setlow et al., 1992; Harper, 2021; Summers, 2021). Ironically, the rise of antibiotic resistance has renewed interest in phage therapy as a viable alternative, highlighting the cyclical nature of history in addressing infectious diseases.

Reasons for the benefits of phage therapy over antibiotics:

Bacteriophages are specific to their host compared to antibiotics multiply in the same area and do not damage other organs. Still, antibiotics attack both the pathogen and the normal flora, which in some cases can cause secondary infection. Of course, the therapeutic use of phage with a combination of antibiotics can be a valuable method, although bacteriophage can be used alone against bacteria with a lytic mechanism (Golkar et al., 2014; Danis-Wlodarczyk et al., 2021; Meneses et al., 2023). Phage therapy’s effectiveness against resistant bacteria to most commercially available antibiotics, if not all of them, has been thoroughly investigated *in vitro*, *in vivo*, and with human subjects (Melo et al., 2020; Taati Moghadam et al., 2020; Liu et al., 2021; Gomez-Ochoa et al., 2022). **Tables 1** and **2** highlight the many *in vitro* and *in vivo* investigations that have demonstrated the intense antibacterial action of bacteriophages against MDR microorganisms. Phage therapy’s effectiveness was assessed against several MDR bacterial taxa, including *A. baumannii*, a pathogen that is highly challenging to treat with conventional antibiotics (Peleg et al., 2008). Phage treatment has been demonstrated in studies to be very successful against isolates of MDR *A. baumannii* that are resistant to carbapenem and colistin (Zhou et al., 2018; Ebrahimi et al., 2021; Rai and Kumar, 2022). The therapeutic efficacy of polymyxin B and two bacteriophages against carbapenem-resistant *A. baumannii* was compared by Zhou et al (Zhou et al., 2018). The study showed that phage treatment boosted the survival of *A. baumannii*-infected larvae by up to 75% using the Galleria mellonella larva model. In comparison, only 25% more larvae infected with *A. baumannii* survived when exposed to polymyxin B (Zhou et al., 2018). Another research investigation evaluated the bacteriophages’ efficacy in eradicating colistin-resistant *A. baumannii* (Ebrahimi et al., 2021). A single phage treatment lasting only 40 minutes resulted in a significant reduction in the quantity of colistin-resistant *A. baumannii*, according to the study (Ebrahimi et al., 2021). Moreover, phage therapy was successful in combating XDR *A. baumannii* strains. Wang and colleagues (Wang et al., 2018) evaluated the ϕkm18p phage’s ability to eradicate XDR *A. baumannii*. The study demonstrated that treating mice infected with XDR *A. baumannii* with mono-phage enhanced survival by up to nearly 100%. Also, in both *in vitro* and *in vivo* investigations, the antibacterial efficacy of bacteriophages against MDR *P. aeruginosa* was assessed (Shokri et al., 2017; Arumugam et al., 2022; Kamer et al., 2022). These investigations indicate that bacteriophages have a strong killing ability against MDR *P. aeruginosa*. Phage cocktail, for example, has been tried against *P. aeruginosa* resistant to colistin (Shokri et al., 2017; Yuan et al., 2019). Psu1, Psu2, and Psu3 phages destroyed the colistin-resistant *P. aeruginosa* bacterial cells (Shokri et al., 2017). Another research study examined the antibacterial efficacy of a lytic phage known as vB_PaeM_LS1 against clinical isolates of *P. aeruginosa*, including MDR strains. The Phage vB_PaeM_LS1 demonstrated strong antibacterial effects against MDR and non-MDR *P. aeruginosa* isolates (Yuan et al., 2019). Phage therapy works well against Gram-negative bacterial infections but also against Gram-positive bacterial infections like MRSA (Takemura-Uchiyama et al., 2014; Al-Ouqaili, 2018; Ding et al., 2018; Barros et al., 2019). The effectiveness of bacteriophages as a treatment for MRSA infections has been shown in several trials (Ding et al., 2018; Barros et al., 2019; Ferry et al., 2020; Ramirez-Sanchez et al., 2021). Takemura-Uchiyama et al (Takemura-Uchiyama et al., 2014), for example, conducted a preclinical investigation to assess the effectiveness of phage S13¢ against hospital-acquired MRSA isolates using a mouse model of lung-derived septicemia. Six hours (h) after infection, phage S13¢ was injected intraperitoneally into MRSA-infected mice. According to the study, mice afflicted with a deadly dosage of MRSA were saved by phage S13¢. On day 5, the survival rates of the phage-treated mice were much more significant (67% vs. 10%) than those of the untreated animals. Based on these findings, S13¢ can save mice from a fatal dosage of MRSA (Takemura-Uchiyama et al., 2014).

**Table 1:** Research conducted *in vitro* settings has investigated the effectiveness of bacteriophages or their derived enzymes in combating MDR bacteria.

Organism	Bacteriophage	Outcome	Reference
Colistin-resistant *P. aeroginosa*	Phage cocktails (Psu1, Psu2, and Psu3)	The phage cocktail demonstrated efficient lysis of MDR *P. aeruginosa*.	(Shokri et al., 2017)
Colistin-resistant MDR *A. baumannii*	IsfAB78 phage	The IsfAB78 bacteriophage demonstrated notable lytic activity against MDR *A. baumannii*	(Ebrahimi et al., 2021)
MDR *A. baumannii*, *K. pneumonia*, and *P. aeruginosa*	Endolysin ElyA1	ElyA1 demonstrated efficacy against all 25 strains of *P. aeruginosa* and *A. baumannii* that were tested, exhibiting susceptibility. Thirteen of the seventeen *K. pneumoniae* isolates exhibited susceptibility to ElyA1, resulting in a reduction of bacterial load by at least two log10 units.	(Blasco et al., 2020)
MDR *K. pneumoniae*	ZCKP1	————	(Taha et al., 2018)
VRE, MRSA, and *E. coli*	Vb_saum_LM12, vb_efas_LM99 vb_ecom_JB75	The three bacteriophages exhibited notable inhibitory properties against MDR strains of *E. coli*, *E. faecalis*, and *S. aureus*.	(Barros et al., 2019)
MDR *P. aeruginosa*	Bacteriophage vb_paem_LS1	The bacteriophage vb_paem_LS1 demonstrated antimicrobial effects against various strains of *P. aeruginosa*, including those resistant to multiple drugs.	(Yuan et al., 2019)
MDR *P. aeruginosa*	EM Phage	Synergistic activity with meropenem and colistin; triple combinations (phage-meropenem-colistin and phage-ciprofloxacin-colistin) achieved up to 4.50 log10 CFU reduction, restored ciprofloxacin and meropenem susceptibility, reduced OMV production and minimized resistance development.	(Holger et al., 2022)
MDR *K. pneumoniae*	ZCKP8 Phage	Lytic phage ZCKP8 (Siphoviridae) demonstrated dose-dependent inhibition of *K. pneumoniae in vitro*. It showed resistance to high temperatures (<70°C), UV stability for 45 min, and activity at pH 5. In a rat wound model, phage treatment significantly reduced infection and improved wound healing, as histological examination shows.	(Fayez et al., 2021)

**Table 2:** *In vivo*, research studies have utilized phage therapy to combat MDR bacteria.

Organism	Model	Bacteriophage	Outcome	Limitations of the Study	Reference
MDR *A. baumannii*	Galleria mellonella larva Murine skin and murine lung infection models	Endolysin colistin and ElyA1	The survival rate of the treated larvae was enhanced by the concurrent administration of colistin and ElyA1. The concurrent administration of colistin and ElyA1 resulted in a reduction of bacterial burden within the skin wounds of the mice that received the treatment.	There is a lack of investigation into the precise mechanism of interaction between ElyA1 and colistin. Focus is limited to three specific MDR pathogens (*A. baumannii*, *P. aeruginosa*, and *K. pneumoniae*).	(Blasco et al., 2020)
Carbapenem resistant *A. baumannii*	Galleria mellonella larva	2 lytic phages (WCHABP12 and WCHABP1)	Either phage WCHABP12 or WCHABP1 protected the larvae against a lethal dosage of *A. baumannii*.	Lack of identification and functional characterization of the novel holin-like proteins potentially involved in the lytic cycle. Absence of experiments to determine the bacteriophages’ efficacy in more advanced animal models or clinical settings.	(Zhou et al., 2018)
Vancomycin-intermediate *Staphylococcus aureus* (VISA), MRSA	Mice	AB-SA01	The treatment led to a significant reduction in the bacterial load present in the lungs of mice.	While AB-SA01 showed high efficacy against *Staphylococcus aureus* strains, rare or atypical bacterial strains that might exhibit resistance were not comprehensively evaluated in this study. The potential synergistic effects of AB-SA01 when combined with other antibiotics or therapies have not been thoroughly explored, which could be critical in clinical scenarios.	(Lehman et al., 2019)
MRSA	Mouse model of lung-derived septicemia	S130’	This resulted in a notable rise in the survival rate of the mice that received treatment.	Findings are based on mouse models, which may not fully replicate human pathophysiology. The potential development of phage-neutralizing antibodies in hosts was not addressed.	(Takemura-Uchiyama et al., 2014)
MRSA	Nude mice	Phage JD007	Inhibited the formation of dermal abscesses caused by *S. aureus*. Phage JD007 failed to elicit a strong immune response in mice treated with it.	Limited targeting specific bacterial strains; may require phage cocktails for broader application. Results based on a nude mouse model may not fully replicate human immune system complexities. Limited analysis of long-term cytokine changes and their implications during phage therapy.	(Ding et al., 2018)
Carbapenem-resistant *A. baumannii*	Mice	Phage SH-Ab15519	Improved the survival rate in the group of mice that underwent the treatment.	The therapeutic efficacy of phage SH-Ab15519 was evaluated against a limited number of *A. baumannii* strains, which may not represent broader genetic diversity. Potential long-term effects of phage therapy, including immune responses or the emergence of phage-resistant bacterial strains, were not assessed.	(Hua et al., 2018)
MDR *K. pneumoniae*		Phage 1513	The treatment led to an increased survival rate in the mice.	The efficacy of phage 1513 was evaluated against a single clinical strain of *Klebsiella pneumoniae*, which may limit its applicability to other strains with different genetic profiles. The long-term safety and potential immune responses to repeated phage administration were not assessed.	(Cao et al., 2015)
XDR *A. baumannii*		ϕkm18p	Improved the survival rate of the treated mice and reduced the bacterial burden within them.	Phage-resistant mutants were observed after therapy, which could limit the long-term efficacy of the phage treatment. The phage φkm18p had a narrow host range, which may reduce its effectiveness across diverse *A. baumannii* strains.	(Wang et al., 2018)

While bacteriophages efficiently eliminate MDR strains, their size increases the possibility of adverse immunological reactions (Loc-Carrillo and Abedon, 2011). To address this obstacle, rather than employing whole phages, a portion of the phage elements can be harnessed as an antibacterial agent. Endolysins, for example, are phage-encoded enzymes that break down the cell walls of both Gram-positive and Gram-negative bacteria (Haddad Kashani et al., 2018). The bactericidal effectiveness of endolysins is notably higher in Gram-positive bacteria due to the accessibility of the peptidoglycan layer. Since Gram-negative bacteria have an outside membrane layer around the peptidoglycan layer, many endolysins have less access to their targets (Haddad Kashani et al., 2018). One of the challenges associated with phage therapy is the emergence of phage-resistant bacteria. However, studies have demonstrated that this phenomenon does not always result in harmful outcomes. For example, mutations leading to phage resistance can alter bacterial phenotypes, including their susceptibility to antibiotics such as daptomycin (Taati Moghadam et al., 2020; Pal et al., 2024). Hood et al. highlighted this interaction, and more recently, Sumrall and colleagues demonstrated that when the virulent phage A511 is exposed to *Listeria monocytogenes* 1042 under laboratory conditions, the presence of glucose and galactose can remove teichoic acids from the bacterial cell wall. This modification is characteristic of pathogenic serotype b and results in the conversion of the bacteria into a less invasive serum form (Cerveny et al., 2002; Sumrall et al., 2019).

Furthermore, Capparelli and colleagues found that phage-resistant *Staphylococcus aureus* strains can potentially be utilized for vaccine production, illustrating a beneficial application of phage resistance (Capparelli et al., 2007; Capparelli et al., 2010).

To increase the effectiveness of phages on resistant bacteria, they are used in different ways:

1. Engineered phages: by modifying the phage genome, they increase the antibacterial capacity of the phage. Treated with antibiotics, however, the effect of phage has not been reported alone (Meile et al., 2022).
2. Phage encapsulation: This process utilizes NPs, such as polyethylene glycol-based platforms. While encapsulation effectively prevents bacteria from accessing the phage, it offers additional benefits, as it is less susceptible to the patient’s immune system. Consequently, the phage remains in the bloodstream for an extended duration, lasting six times longer than in its unencapsulated form (Loh et al., 2021).
3. Use of phage components: Phage components are tiny, similar in size to antibiotic molecules, and, unlike complete phages, they cannot replicate within the host, making them easier to administer. One notable example is PA-PP, a serine protease that degrades the outer membrane porin protein. This type of phage-derived enzyme produced during the phage life cycle, has recently emerged as an up-and-coming alternative to conventional antibiotics (Miernikiewicz et al., . 2023; Ssekatawa et al., 2021; Wang et al., 2024).
4. The use of phage cocktails that consist of several phages with different mechanisms of action. This cocktail is composed of 2 to 10 phages. This antibacterial strategy is unsafe because if the phages are not stored in a sufficient dose in the cocktail or do not attack the same bacteria, they cause resistance (Duc et al., 2023).
5. Use of phages in combination with antibiotics: The potential of combining phages with antibiotics was first reported in 2007. In what is known as phage-antibiotic synergy (PAS), the activity of phages is enhanced when used in conjunction with sub-inhibitory concentrations of antibiotics (Nilsson, 2014; Tagliaferri et al., 2019). Garcia and Johnson used a combination of phage MR-5 with linezolid to inhibit the formation of biofilms created by MRSA. They found that the two together had a more significant effect on bacterial colonization than either alone (Kaur et al., 2014). Another study on *Pseudomonas aeruginosa* revealed that the simultaneous use of phages with antibiotics, even when the host bacteria were antibiotic-resistant, could increase bacterial susceptibility to antibiotics. This phenomenon occurs as antibiotic exposure sensitizes the bacteria, facilitating the phage’s ability to exert selective pressure. Combination therapy presents a highly effective solution for managing bacterial infections. However, to achieve optimal outcomes, further research involving human and *in vivo* models is necessary to explore interactions between phages, antibiotics, and human tissues (Caflisch et al., 2019; Li et al., 2021; Zhao et al., 2024).

#### 6.1.1 Phage therapy: regulatory challenges

Phage therapy, despite its immense potential, encounters notable challenges that hinder its widespread clinical adoption. A major challenge is the lack of a clear regulatory pathway and concerns over intellectual property rights, as phages differ fundamentally from conventional therapeutics. Unlike antibiotics, which can be chemically defined and patented, phages are biological entities that are harder to patent due to global legal precedents limiting the ownership of natural organisms or genes. Modifying existing phages, creating phage cocktails, or synthesizing phages from scratch are potential avenues for securing intellectual property, though the latter is costly and often unnecessary (Kingwell, 2015; Barbu et al., 2016; Anomaly, 2020).

Governments, rather than private enterprises, are urged to fund phage research due to the uncertainty of financial returns and the niche nature of phage therapy. Proposals such as establishing publicly funded phage libraries can help address issues of access and availability while encouraging broader research and application. These repositories, hosted by national health organizations and authorized labs, would catalog and distribute diverse phages for therapeutic use (Anomaly, 2020).

Incentives like transferable patent extensions and monetary prizes could further stimulate private investment. These measures would provide financial rewards for developing innovative therapies targeting difficult-to-treat infections, potentially bridging the gap between regulatory challenges and market viability. Regulatory bodies like the FDA require robust clinical evidence of safety and efficacy before approving phage therapies for human diseases, emphasizing the need for streamlined processes to support this innovative treatment approach (Spellberg, 2009; Anomaly, 2020).

### 6.2 Probiotics, postbiotics, and synbiotics

As per the recommendations outlined by the World Gastroenterology Organization, probiotics are defined as living microorganisms that, when ingested in sufficient quantities, provide advantageous effects on the individual’s health (Organisation, 2017; Baryshnikova et al., 2023). Pharmaceutical interventions targeting the gut microbiota can be classified into distinct categories: authentic probiotics (comprising live microorganisms), prebiotics (comprising compounds that act as nourishment for microorganisms), synbiotics (a blend of probiotics and prebiotics), and symbiotics (a blend of diverse probiotics) (Chaudhari and Dwivedi, 2022; Yarahmadi and Afkhami, 2024). Probiotics have gained popularity among individuals advocating for healthy living and are frequently utilized as functional foods or dietary supplements in the US and EU nations. These products are readily available in supermarkets, pharmacies, and online platforms (Liu et al., 2018). Various well-known probiotic microorganisms include different species of Bifidobacterium (such as *B. infantis*, *B. bifidum*, *B. longum*, *B. adolescents*, and *B. breve*), Lactobacillus (including *L. acidophilus*, *L. casei*, *L. plantarum*, *L. bulgaricus*, *L. reuteri*, *L. lactis*, *L. fermentum*, *L. rhamnosus*, *L. johnsonii*, and *L. gassed*, *L. paracasei*), non-pathogenic strains of Enterococcus (such as *E. faecium* and *E. salivarius*), certain non-pathogenic species of *E. coli*, non-pathogenic *Bacillus* spp. (specifically *Bacillus subtilis*), lactic acid streptococci (*S. thermophilus*), yeast fungi like *Saccharomyces boulardii*, and newer variations like probiotic products containing *Clostridium butyricum* or *Akkermansia muciniphila* (Liu et al., 2018; Jain et al., 2023). Probiotics have a history of use in the treatment of infectious disorders. Because of their capacity to obstruct pathogen development, quorum sensing, and biofilm formation, they may prove to be a helpful weapon against newly developing resistant pathogens (Lebeer et al., 2010; Sassone-Corsi and Raffatellu, 2015; Raheem et al., 2021; Fernández-Alonso et al., 2022). Additionally, probiotic bacteria generate bioactive substances with antimicrobial qualities (such as enzymes, cell wall fragments, AMPs, exopolysaccharides, and numerous other bioactive compounds) (Żółkiewicz et al., 2020). Among probiotics, lactic acid bacteria, especially *Lactobacillus* species, are frequently utilized as supplemental agents in anti-*Helicobacter pylori* (*H. pylori*) treatment (Hu et al., 2021; Penumetcha et al., 2021). The potential incorporation of lactobacilli or bifidobacteria as supplementary agents in eradication therapy shows promise due to the bacteriocins they produce, which possess inhibitory effects on the proliferation of *H. pylori* and interfere with its attachment to the epithelial cells of the stomach (Kim et al., 2003; Penumetcha et al., 2021; Yang et al., 2024). In a randomized controlled trial, a combination of probiotics (*Lactobacillus Acidophilus* LA-5, *Lactiplantibacillus plantarum*, *Saccharomyces boulardii*, and *Bifidobacterium lactis* BB-12) along with four antibiotics (omeprazole, clarithromycin, amoxicillin, and metronidazole) was administered for the treatment of *H. pylori* infection. The findings demonstrated that the experimental group using probiotics had a 92 percent cure rate for *H. pylori*, compared to the control group’s 86.8 percent cure rate (Viazis et al., 2022). Probiotics are therefore an effective additional therapy option for *H. pylori* infection. Probiotics are essential for treating *H. pylori* infection because they not only stop urease activity but also prevent *H. pylori* from adhering to host cells (Liu et al., 2024).

According to Lahtinen et al (Lahtinen et al., 2007), 3 of the 38 *Bifidobacterium* strains isolated from old persons stopped *S. aureus* from growing. Piewengam et al (Piewngam et al., 2018). showed that lipopeptides produced by *Bacillus* species prevent *S. aureus* from detecting quorum. Lastly, the generation of virulence factors (such as protease, pyocyanin, and rhamnolipid) with *P. aeruginosa* was decreased by the probiotic *Pediococcus acidilactici* HW01 (Lee et al., 2020). The supernatant from *L. plantarum* CIRM653 markedly inhibited *K. pneumoniae* biofilm development, according to research by Lagrafeuille et al (Lagrafeuille et al., 2018). The supernatant downregulated operons essential for quorum detection and disrupted the activation of genes linked to biofilms. Furthermore, *Bifidobacterium longum* 5(1A) decreased *K. pneumoniae* infection in mice by enhancing neutrophil recruitment, lowering bacterial burden, and generating proinflammatory cytokines (Vieira et al., 2016). The use of probiotics can prevent the colonization of the colon microbiota. Among the probiotics that are effective in colonizing *A. baumannii*, *P. aeruginosa* and Candida albicans can be found in *L. plantarum*., *L.fermentum* pointed out (Wieërs et al., 2020). It has been observed that VRE is less decolonized if Barenzilla (*L. paracasei*) is used in patients with leukemia (Scher et al., 2013). In a study by Spalton and colleagues (Stapleton et al., 2011) on premenopausal women with a history of urinary tract infections, they prescribed lactin-v (*Lactobacillus crispatus*) or placebo daily for 5 days and then once a week for 10 weeks. The results showed that patients treated with *Lactobacillus* vaginally had a significant reduction in UTI episodes compared with those who received placebo. Lactobacilli could be used to treat recurrent urinary tract infections, and long-term use of antibiotics in women. be useful (Wieërs et al., 2020). If microbiota can be modified with probiotics, the problem of drug resistance will be significantly reduced. However, further study is needed to understand better the material released by probiotic bacteria and the parabiotic effect of inactive bacterial cells (Taverniti and Guglielmetti, 2011; Fernández-Alonso et al., 2022).

### 6.3 Fecal microbiota transplantation

FMT involves transferring minimally processed fecal material from a healthy donor into the gastrointestinal (GI) tract of an individual with a medical condition, aiming to provide therapeutic benefits. FMT has its roots in the fourth century in China, when treating GI disorders such as food poisoning and severe diarrhea using human feces suspension was recommended and shown to be effective (Zhang et al., 2012; Yarahmadi et al., 2024a). Ben Eiseman, the chief surgeon of Denver General Hospital, reported on four patients with pseudomembranous colitis in the contemporary era of medicine who recovered following rectal instillation of donor feces (Eiseman et al., 1958). Remarkably, he carried out FMT in 1958, almost two decades before the discovery that *Clostridium difficile (C. difficile*) was the cause of antibiotic-induced pseudomembranous colitis. “This simple yet rational therapeutic method should be given more extensive clinical evaluation,” he said, noting “immediate and dramatic” reactions (Eiseman et al., 1958). Around 2008, a *C. difficile* infection pandemic caused interest in FMT to reemerge. FMT is the procedure of introducing a suspension of commensal bacteria-containing feces from a healthy person donor into the recipient’s intestinal lumen by a variety of techniques, such as colonoscopy, nasogastric, nasoduodenal, and enema (Bakken et al., 2011; Smits et al., 2013; Cheng and Fischer, 2023). Few studies have demonstrated the efficacy of FMT as a treatment plan for patients with *C. difficile* infection in addition to other GI disorders like colitis, irritable bowel syndrome, constipation, diarrhea, and several neurological conditions like multiple sclerosis and Parkinson’s disease (Kumar et al., 2021; Matheson and Holsinger, 2023). One of the most prevalent super germs in the modern era is *C. difficile* (Lawson et al., 2016). Due to the extensive use of antibiotics and the resulting alteration of the intrinsic gut flora, which leaves the host vulnerable to *C. difficile* invasion, colonization, and infection, the outbreak has grown to epidemic proportions (Khoruts and Sadowsky, 2016). Antibiotics are the principal treatment for *C. difficile* infections; however, while they efficiently eliminate the infection, they paradoxically make the dysbiosis worse (Thorpe et al., 2018). Through a variety of methods of action, FMT rapidly restores microbial diversity and the dominance of protective species, inhibiting the activity of *C. difficile* (Cheng and Fischer, 2023).

The interest in the potential of FMT to eliminate or decrease the presence of Antimicrobial-Resistant Organisms (ARO) in the GI tract was sparked by findings in individuals with *C. difficile* infection, as documented in studies like the one conducted by Millan and colleagues (Millan et al., 2016). Before and after FMT, stool samples from patients with *C. difficile* infection and FMT donors were examined. Comparing donors to a “healthy” control group revealed that the burden of antibiotic resistance gene (ARG) in their global resistome was similar (average of 3.4 vs. 6.0 ARG), primarily due to tetracycline resistance, whereas patients with *C. difficile* infection had an average of >30 ARG. ARG was more varied, indicating resistance to fluoroquinolone, beta-lactams, and multidrug efflux pumps in individuals infected with *C. difficile*. In addition, it was observed that FMT patients who “responded” to FMT—that is, who did not further develop a *C. difficile* infection—had lower levels of ARG carriage than non-responders. This decrease lasted a minimum of a year (Millan et al., 2016; Lamberte and an Schaik, 2022). A *post hoc* analysis of the PUNCH CD trial, which looked at RBX2660, a “microbiota restoration therapy” similar to FMT, as a treatment for *C. difficile* infection, produced similar findings. Researchers discovered that RBX2660 decreased the number of Enterobacterales resistant to antibiotics in patients. The degree of donor microbiota engraftment was correlated with a reduction in ARG carriage (Langdon et al., 2021). The same medicine, currently sold under the name “REBYOTATM,” was granted a license in late 2022 to treat *C. difficile* infections (Hunt et al., 2024).

Clinical research has shown that autologous FMT (aFMT) is superior to probiotic treatment and causes the GI microbiota of antibiotic-treated human patients to recover quickly and nearly completely (Suez et al., 2018). The results of an intention-to-treat clinical study with 22 patients in the donor FMT group showed that 20 of the 22 patients (90.9%) had a clinical cure for their *C. difficile* infection. The clinical recovery success rate exceeded that of aFMT at 62.5%. Furthermore, FMT from donors effectively reinstated gut microbial diversity and functionality in recipients to levels comparable to those of the donors (Kelly et al., 2016).

FMT has demonstrated promising outcomes in pediatric cases. The first successfully treated pediatric patient was a 14-year-old girl diagnosed with hemophagocytic lymphohistiocytosis, who experienced recurrent infections caused by carbapenemase-producing *Klebsiella pneumoniae*. Following a single FMT, no recurrence was observed over a 1.5-year follow-up period (Freedman and Eppes, 1805). In another case, a 16-year-old girl with acute myelogenous leukemia received two FMTs to address colonization by VRE and carbapenemase-producing bacteria. The treatment successfully decolonized VRE, although colonization by carbapenemase-producing bacteria persisted. Notably, no adverse events were reported in either case (Battipaglia et al., 2019). Additionally, five patients who had undergone hematopoietic stem cell transplantation and were colonized with MDR bacteria were treated with FMT. While four out of the five patients achieved decolonization within one week, they all experienced recolonization within one month (Merli et al., 2020). Ongoing clinical trials (ClinicalTrials.gov identifiers: NCT06156956, NCT04593368, and NCT02543866) are currently enrolling participants to evaluate the effectiveness of FMT in decolonizing antibiotic-resistant bacteria in larger pediatric populations.

### 6.4 Nanoparticles

Due to the urgent need to develop new and alternative antibacterial agents for drug resistance, nanoscience has made significant progress in recent years. Metal NPs have shown substantial progress in recent years as potent antibacterial agents and alternatives to drug resistance. This ability of NPs can be attributed to their unique physical and chemical properties. Based on their surface charge, NPs can be attached to components of the pathogen surface that have the opposite charge (Franco et al., 2022; Ye et al., 2022). In addition to serving as vehicles for specific drug delivery, NPs can also possess antibacterial properties of their own through a variety of mechanisms, including biofilm inhibition, bacterial wall disruption, host immune response modulation, reactive oxygen species (ROS) generation, and damage to the resistant bacteria’s essential DNA and protein molecules (Ozdal and Gurkok, 2022). NPs can be used as carriers of antibiotics or other small molecules like antibodies or chemotherapeutic drugs since their diameters are more significant than antibiotics (Puiu et al., 2021). One of the most often described processes linked to metal NPs is the suppression of protein synthesis and damage to DNA. These disintegrate the enzymes, other proteins generated in the bacterial cell membranes, and ribosomal subunit proteins. Likewise, there has been evidence of bacterial DNA degradation, compression, and fragmentation, which has decreased the physiological function of genes (Yougbaré et al., 2021; Franco et al., 2022).

Zinc oxide nanoparticles (ZnONPs) were employed in a study by Su et al (Su et al., 2015). to investigate their impact on *E. coli* DNA. The researchers observed that the nanoparticles caused significant damage in 10 specific regions of the bacterial genome. Additionally, ZnONPs were found to alter gene expression, ribosome composition, molecular structure-activity relationships, and RNA modifications in *E. coli*. These findings highlight the multifaceted effects of ZnONPs on bacterial molecular pathways and their potential as antimicrobial agents. Similarly, Nagy et al (Nagy et al., 2011). used silver NPs to damage DNA by positively regulating several antioxidant genes, ATPase pumps, metal depletion, and genes encoding metal transport in *E. coli* and *S. aureus*. This investigation led them to the conclusion that bacteria’s antioxidant ability is reduced by the antibacterial action of silver NPs.

To increase the efficacy of drug delivery to *H. pylori* colonization sites and boost the rate of *H. pylori* eradication, a drug delivery system must be developed to shield the medication from the stomach’s acidic environment (Luo et al., 2018). Typically measuring less than 100 nm, NPs are the most widely utilized delivery carriers. Due to their substantial specific surface area, NPs can transport extra medications to their intended location (Sousa et al., 2022). The most popular carrier for nano-delivery methods is chitosan. It can pass through mucous layer pores to the stomach epithelium’s surface and transport medications to the *H. pylori* infection location for therapy (Sun et al., 2022). Chitosan demonstrates superior biocompatibility and effectively adheres to the gastric mucosa, extending the drug’s residence time within the target cells (Sousa et al., 2022; Yarahmadi et al., 2024b).

Metal NPs include silver copper oxide hybrid, which due to its radical properties can produce hydrogen peroxide, which disrupts the metabolic process of bacteria. Alone has a history of treatment as copper has been used in the treatment of wounds and cleansing of drinking water. It has also been used as a biosensor, dye, and antimicrobial industry that shows different effects in contact with pathogens (Mohanraj, 2017; Baptista et al., 2018). The healthcare industry has been dramatically impacted by the antibacterial ability of silver NPs, which are used to create bactericidal coatings for medical equipment. Additionally, they can be found in textiles, packaging materials, and cosmetics (Ibrahim Khan and Khan, 2017).

Numerous investigations have demonstrated the exceptional antibacterial activity of cerium oxide nanoparticles (CNPs) (Farias et al., 2018; Zhang et al., 2019; Pop et al., 2020; Zamani et al., 2021). The antibacterial properties of CNPs against *S. aureus* have been shown extensively, with studies highlighting their potent inhibitory effects (Thill et al., 2006; Krishnamoorthy et al., 2014; Arumugam et al., 2015; Gopinath et al., 2015; Reddy Yadav et al., 2016; Surendra et al., 2016). Additionally, *P. aeruginosa’s* sensitivity to CNPs has been evaluated and confirmed through various methods, including agar diffusion assays and microdilution tests (Ravishankar et al., . 2015; Arumugam et al., 2015; Masadeh et al., 2015).

Iron oxide nanoparticles (IONPs) are versatile materials utilized in various applications, including serving as contrast agents for magnetic resonance imaging (MRI), biosensors, disease diagnostics, drug delivery systems, pollutant removal, biomedical devices, and antimicrobial agents (Negrescu et al., . 2022; Sakowicz and Lojewska, 2021a; Dadfar et al., 2019; Fernández Llamas et al., 2020; Sánchez-López et al., 2020; Shakil et al., 2023). While divalent metals such as iron ions are critical for microbial growth, IONPs exhibit broad-spectrum antibacterial activity. This activity is primarily mediated through mechanisms such as the generation of ROS and electrostatic interactions, which can involve attractive and repulsive forces (Slavin et al., 2017; Makabenta et al., 2021; Hochvaldová et al., 2022; Karnwal et al., 2023). Furthermore, IONPs’ antibacterial activity may be influenced by additional factors such as synthesis techniques, precursors, size, and concentration (Assa et al., 2016). Gudkov et al (Gudkov et al., 2021). reported that the antibacterial properties of IONPs can be influenced by modifications in their synthesis methods and size. They identified several bacteriostatic mechanisms, including the generation of ROS, electrostatic interactions, disruption of the bacterial cell membrane, and the fragmentation of DNA and proteins through the induction of free radicals. Additionally, Alprol et al (Alprol et al., 2023). emphasized that IONPs synthesized through green methods demonstrate strong antibacterial effects against Gram-positive bacteria and moderate inhibition of Gram-negative bacteria, primarily by inducing oxidative stress through ROS generation.

As different bacterial strains have different cell wall structures, the antibacterial efficacy of IONPs also varies by strain. The antibacterial efficacy of IONPs is influenced by the bacterial strain, owing to variations in cell wall structure. Gram-positive bacteria possess a thick peptidoglycan layer with a net negative charge, primarily due to the presence of teichoic acid (Swoboda et al., 2010). In contrast, Gram-negative bacteria have a thinner peptidoglycan layer covered by an additional LPS layer, which also carries a higher net negative charge. IONPs interact with the negatively charged bacterial membranes through electrostatic forces, leading to membrane depolarization and disruption of membrane integrity (Azam et al., 2012; Ismail et al., 2015; de Lacerda Coriolano et al., 2021). As a result, due to these structural differences, Gram-positive bacteria are generally more susceptible to IONPs than Gram-negative bacteria (Nakai and Tsuruta, 2021; Shoudho et al., 2024). In addition to NPs derived from cerium oxide and iron oxide, natural plant extracts have also been explored for their antimicrobial potential.

For instance, in one study, *Murraya koenigii* (Curry leaf) was used as a source of nano-hybrid production, which was used against drug-resistant pathogens. This experiment was performed in several ways and was approved. The agenda and the experiment were performed in three ways:

1. Through good propagation and use of experimental pathogens of *A. baumannii* 211 and 210, *P. aeruginosa* strain 40, *S. aureus* resistant to methicillin and *E. coli* 55 and adding nano-hybrid particles and examining the restraint zone throughout the well (Baker et al., 2020).
2. Using the broth dilution method, which increases the concentration of nan hybrid particles, and finally uses a spectrophotometer with visible UV light at a wavelength of 600 nm (growth pattern was examined) (Syed et al., 2019).
3. Using the method of minimum inhibitory concentration. In this method, the lowest concentration of NPs that can inhibit the growth of the desired pathogen was investigated; it is almost similar to the MIC method for antibiotics (Syed et al., 2017).

With all the methods mentioned above, the antimicrobial activity of nano-hybrids prepared from Murraya koenigii plant on the experimental pathogens mentioned above was confirmed by the fact that in the first method, which was the release of a well, the inhibitory zone above the well indicates repression. The second method, which was the dilution of broth, showed the activity of NPs based on the concentration of broth. The lowest concentration of NPs was found to suppress the growth of experimental pathogens (Baker et al., 2020).

#### 6.4.1 Nanoparticles: challenges and limitations

Lab-scale production of nanomaterials is simple. However, the limitations of the sophisticated experimental equipment and the lack of sufficient understanding of scale-up techniques make large-scale manufacturing seem like a challenging task. The difficulties in reproducing the preparatory process and scaling up the approach are also perceived to have negative impacts (Muthu and Wilson, 2012; Afkhami et al., 2024). Among the challenges faced during the production of nanomaterials are contaminants, insufficient batch-to-batch variability, poor quality control, biocompatibility, chemical instability, low production yield, high cost, scalability issues, lack of infrastructure, government regulations, and lack of funding (Baig et al., 2021; Chen et al., 2022).

The toxicity of NPs remains one of the main problems. Factors influencing toxicity include surface area, particle size and shape, solubility, and aggregation. Because of their tiny size, physiological barriers may be broken down, which might harm one’s health (Ryman-Rasmussen et al., 2006; Jia et al., 2018). There is evidence that free radicals produced by NP harm cellular membranes, organelles, and DNA (Xia et al., 2006). Intracellularly delivered nanomaterials may trigger an immune response by interacting with cell surface receptors (Penn et al., 2005; Vallhov et al., 2006). If pregnant mice are exposed to titanium dioxide NPs, they may cause morphological abnormalities in the growing baby because they can cross the placental barrier (Ferdous and Nemmar, 2020). Additionally, it has been demonstrated that Ag NPs reduce estrogen plasma levels, which impacts embryonic development and increases the frequency of resorbed fetuses (Teng et al., 2016; Ferdous and Nemmar, 2020).

Nanomaterials’ distinct physicochemical characteristics are directly linked to their toxicity; some of these characteristics, however, give goods incorporating nanomaterials more promising performances and a wider range of applications than those of conventional chemicals. The “nanomaterial paradox” has made it challenging for those involved to develop and use nanotechnology without having unfavorable effects. To address this issue, significant national and regional regulatory organizations have been actively monitoring goods enabled by nanotechnology and working together to create a consensus on global standards and regulations (Hamburg, 2012; Rauscher et al., 2017). Products that incorporate nanomaterials are currently governed by the general and industry-specific regulatory and legal frameworks in place (Hamburg, 2012; Soltani and Pouypouy, 2019). For example, nanomaterials are subject to the same regulations as all other chemicals and mixtures in the EU. This means that before they can be imported and used in the EU, they must be registered under the Registration, Evaluation, Authorization, and Restriction of Chemicals regulations. Additionally, nanomaterials that have hazardous properties must be reported to the European Chemicals Agency and must be labeled and packaged by the Classification, Labelling, and Packaging Regulation regulations to ensure their safe use (Bowman et al., 2010; Liu et al., 2022). Moreover, potential hazards are linked to particular nanomaterials and particular applications, according to the European Commission’s “Second Regulatory Review on Nanomaterials” study. Nanomaterials should be evaluated for risk individually using relevant data (Liu et al., 2022). To facilitate the regulation of nanomaterials in novel foods, food contact materials, cosmetic products, and medical devices and to enable consumer, worker, and environmental protection, organizations such as the European Food Safety Authority, European Medicines Agency, Joint Research Centre-Institute for Health and Consumer Protection, and European Agency for Safety and Health at Work have established corresponding guidance documents in this regard (Soltani and Pouypouy, 2019). In a similar vein, the Food and Drug Administration (FDA), one of the most significant regulatory agencies in the United States, upholds its product-focused regulatory policies for products that are enabled by nanotechnology and regulate them under the existing statutory authorities through particular premarket review and/or postmarket oversight systems (Hamburg, 2012). The industry is in charge of making sure that a product satisfies all applicable safety standards and other regulations, regardless of whether it is subject to premarket evaluation (for example, new pharmaceuticals and biological products) or not (for example, cosmetics) (Hamburg, 2012). To assist the industry in fulfilling its statutory responsibilities, the FDA also promotes early industry interaction and provides both general and particular technical guidance on various subjects (Hamburg, 2012). The Environmental Protection Agency is another significant US government agency that oversees nanomaterials. It established several environmental regulations, such as the Federal Insecticide, Fungicide, and Rodenticide Act, the Toxic Substances Control Act, the Clean Air Act, the Clean Water Act, and others, to control nanomaterials during their production, use, commercial distribution, disposal, and release into the environment (Hanson et al., 2011). Other countries regulate nanomaterials and ordinary chemicals through shared legal acts, such as Japan’s Act on the Evaluation of Chemical Substances and Regulation of Their Manufacture, Food Sanitation Act, and Pharmaceutical and Medical Device Act, and Korea’s Quality Management & Safety Control of Industrial Products Act, Industrial Safety & Health Act, and Toxic Chemical Control Act, among others (Rasmussen et al., 2017). To recap, while nanomaterials now share similar regulatory and legislative frameworks with regular chemicals, practically all regulatory authorities pay special attention to nanomaterials and issue guidelines or standardizations for almost every stage of their safety assessment (Rasmussen et al., 2017; Miernicki et al., 2019).

### 6.5 Antimicrobial peptides

AMPs are low molecular weight proteins with potent antimicrobial activity, playing a crucial role in modulating the immune response against various pathogens, including bacteria, viruses, and fungi (Erdem Büyükkiraz and Kesmen, 2022). Unlike conventional antibiotics, microorganisms have limited ability to develop resistance to most AMPs, making them highly effective alternatives. These peptides are characterized by low toxicity, biological diversity, direct antimicrobial action, and the ability to reduce drug interactions. To date, 60 peptide-based drugs have been approved for clinical use (Boparai and Sharma, 2020; Erdem Büyükkiraz and Kesmen, 2022).

AMPs were first isolated from *Bacillus* species in 1339, and subsequent studies have identified them in a variety of organisms, including both erythrocytes and prokaryotes. For example, the skin of certain amphibians, such as the *Bombina variegata* frog, contains more than 300 distinct AMPs. Among these, the peptides Bombinin H4 and H2 exhibit strong antimicrobial properties, particularly in combating resistant infections (Andrä et al., 2001; Bednarska et al., 2017; Chen et al., 2022). Additional antimicrobial peptides have been discovered in various organisms, including bovine lactoferrin, human leukocyte lysozyme, and peptides from the female reproductive system. Several AMPs have demonstrated inhibitory effects against pathogens such as *Shigella*, *Salmonella*, *Escherichia coli*, and *Staphylococcus aureus*, often produced by Bacillus species (Boparai and Sharma, 2020; Bin Hafeez et al., 2021; Erdem Büyükkiraz and Kesmen, 2022).

In terms of function, peptides are divided into two groups:

1- membrane 2- non-membrane

Membrane peptides disrupt bacterial membranes, while non-membrane peptides move across the membrane without damaging the membrane but disrupt normal cell function. Since the outer membrane of prokaryotic cells contains LPS. Or do ticoic acids therefore have a negative charge. Cationic AMPs interact with these membranes and cause membrane permeability, thus disrupting the bacterial membrane. At the same time, eukaryotic cells due to the presence of phosphatidylcholine And sphingomyelin phospholipids do not have these interactions (Sani and Separovic, 2016; Boparai and Sharma, 2020; Zhang and Yang, 2022).

AMPs that have more solubility in aqueous media, i.e., have a cationic alpha helix, are capable of causing death by osmotic shock. Some of the uses of these peptides include scorpion peptide in the treatment of eye diseases caused by acanthamoeba and rBPI21, which is used in the treatment of local infections such as open heart surgery in children and severe burns (Conlon and Sonnevend, 2011). Use skin-derived peptides Amphibians for MDR infections caused by *Acinetobacter pneumoniae*, *Bacillus pneumoniae*, and *E. coli* bacteria (Alsterin, Scafin, Psudin, etc.) (Boparai and Sharma, 2020).

Use natural salivary peptides (P113) that have high activity against Gram-positive and Gram-negative and *Candida albicans* are in the form of mouthwash and are used in HIV-positive patients (Yu et al., 2017). It is used in clinical trials due to its unique properties, such as low toxicity of selective efficacy and high strength and broad spectrum (Bach, 2018). MRSA was initially discovered sixty years ago. Infection with MRSA has since spread quickly around the world (Jacobs, 1999). Cbf-K16, an AMP structurally similar to enterotoxin, is a variant of Bf-30 identified in the venom of the Golden Ring Snake. Composed of 30 amino acids, Cbf-K16 has been shown in previous studies to exhibit broad-spectrum antibacterial activity with minimal cytotoxicity. Additionally, it demonstrates potent bactericidal effects against MRSA (Diene et al., 2011; Li et al., 2016).

Additionally, Ib-AMP4, a plant-derived AMP isolated from impatiens seeds, demonstrates significant bactericidal activity against both Gram-positive and Gram-negative bacteria (Flórez-Castillo et al., 2019). In a study by Sadelaji et al (Sadelaji et al., 2022), the antibacterial efficacy of Ib-AMP4 against MRSA was investigated through *in vitro* and *in vivo* experiments. The results confirmed that Ib-AMP4 effectively inhibits MRSA. Scanning Electron Microscopy (SEM) analyses revealed that Ib-AMP4 disrupts MRSA biofilms. Time-kill curve and growth kinetics assessments indicated rapid antibacterial activity. Furthermore, in a mouse model, treatment with Ib-AMP4 led to the complete survival of infected mice, with no detectable bacterial presence in their blood samples (Sadelaji et al., 2022). These findings highlight the strong antibacterial potential of Ib-AMP4, suggesting its possible application as an effective therapeutic agent against MRSA infections.

Recent investigations have highlighted daptomycin as a promising alternative for managing antibiotic-resistant infections, particularly MDR Gram-positive bacterial infections. Daptomycin, a cyclic lipopeptide derived from *Streptomyces roseosporus*, disrupts bacterial cell membrane function by inducing depolarization (Jones et al., 2021; Boulekbache et al., 2024). Preliminary pharmacokinetic data indicate that daptomycin can achieve limited penetration into the cerebrospinal fluid, positioning it as a potential therapeutic option for acute bacterial meningitis in pediatric and adult populations (Chavanet et al., 2023; Jaber and Beahm, 2023). As Jaber et al (Jaber and Beahm, 2023). noted, while daptomycin shows promise as an alternative treatment for Gram-positive bacterial meningitis, additional clinical studies are necessary to refine its dosing strategies, duration of therapy, and therapeutic application.

Another AMP advanced to clinical trials for therapeutic validation is melittin, a bioactive peptide derived from bee venom. Melittin has demonstrated remarkable antibacterial properties, particularly against antibiotic-resistant bacteria, making it a promising candidate for addressing the growing challenge of antimicrobial resistance (Askari et al., 2021; Guha et al., 2021; Memariani and Memariani, 2024). Beyond its antibacterial efficacy, melittin also exhibits antiviral activity against pathogens such as herpes simplex virus (HSV), severe acute respiratory syndrome coronavirus 2 (SARS-CoV-2), and human immunodeficiency virus (HIV) (Zhang et al., 2024). Currently, in Phase 1 clinical trials, melittin is being evaluated primarily for its potential in treating infections caused by resistant bacterial strains, as highlighted by Zhang et al (Zhang et al., 2024).

#### 6.5.1 AMPs: challenges and limitations

A recent study that examined the literature from several databases concluded that AMPs are among the highly desirable research areas for academics with enormous potential in the pharmaceutical industry (Luo et al., 2023). Only a tiny number of AMPs received FDA approval for clinical usage despite their great potential and level of interest. The lengthy and intricate process of drug discovery and development is well known to include several stages, including target identification and discovery, preclinical and clinical trials (phases 1–3), screening and design improvements, and approval. AMPs have their restrictions among them. First, the mechanism of action is still not entirely understood, which is crucial for creating peptides with reduced toxicity and maximized effectiveness (Bucataru and Ciobanasu, 2024).

AMPs face challenges related to stability and limited antibacterial activity. These peptides are prone to protease degradation and are sensitive to factors such as serum components, salt concentrations, and pH fluctuations. Additionally, the electrostatic interactions between AMPs and bacterial membranes can be significantly affected by the ionic strength of the surrounding environment. High salt concentrations, such as those in physiological saline solutions, can impact the stability and efficacy of AMPs. Salt ions may interact with the charged amino acid residues of AMPs, leading to conformational changes that could reduce their antimicrobial effectiveness. Furthermore, salt levels and the hydrophobic nature of AMPs can influence their solubility and tendency to aggregate, further affecting their functional properties (Kang et al., 2014; Lin et al., 2023).

Another factor that can have a significant influence on the stability and activity of AMPs is the pH of the surrounding environment. Most AMPs demonstrate optimal activity in neutral or slightly acidic pH environments, characteristic of many body tissues. For instance, histidine has a pKa of approximately 6.5, and clavanin A, a histidine-rich peptide, exhibits a high net positive charge at pH 5.5. However, at pH 7.4, it becomes relatively uncharged, leading to a significant reduction in its antibacterial activity (Lee et al., 1997). However, excessive pH levels, such as those seen in the stomach or the intestines, can impact the structure and functionality of AMPs (Malik et al., 2016).

In the bloodstream, AMPs are exposed to various serum components, such as proteins and other biomolecules. These components can interact with AMPs, potentially altering their structure or biological activity. Furthermore, serum proteins may bind to AMPs, influencing their distribution, stability, and effectiveness (Dijksteel et al., 2021). Possessing a net positive charge, the LL-37 peptide can attach to anionic serum proteins and turn inactive (Lin et al., . 2020). AMPs are frequently quickly removed from circulation by the liver and kidneys following their proteolytic cleavage. Increased clearance limits the effectiveness of AMPs by lowering their systemic exposure and therapeutic concentrations. To address the instability of AMPs under these conditions, various strategies have been developed to improve their stability, bioavailability, and therapeutic potential. These approaches include modifying the peptide sequence by deleting or substituting specific amino acids, C-terminal amidation, N-terminal acetylation, methylation, replacing L-amino acids with their D-enantiomers, as well as employing techniques such as dimerization, hybridization, or encapsulation within NPs (Chen and Jiang, 2023).

The potential cytotoxicity of AMPs in humans is a critical consideration during preclinical and clinical studies. While AMPs generally exhibit lower toxicity to mammalian cells than many conventional antibiotics, their cytotoxic effects can vary depending on factors such as peptide sequence, concentration, route of administration, and target cell type. Based on their mechanism of action, AMPs may induce toxicity either by binding to specific receptors or disrupting cell membranes. Receptor-binding peptides can modulate the immune system to achieve bacteriostatic effects. However, at high concentrations, AMPs may trigger excessive or dysregulated immune responses, potentially leading to inflammatory secondary conditions such as rosacea, atopic dermatitis, or psoriasis (Chen and Lu, 2020). Melittin and other pore-forming peptides exhibit non-specific toxicity and a lytic impact, even on human cells. This peptide’s antibacterial action is ineffective when used at acceptable dosages (Askari et al., 2021). Melittin is currently undergoing clinical phase I trials. Hydrophobicity plays a crucial role in the antimicrobial activity of peptides by enhancing their ability to integrate into microbial membranes. However, excessive hydrophobicity can compromise the selectivity of AMPs, leading to the disruption of mammalian cell membranes, increased cytotoxicity, and reduced specificity for microbial cells (Kumar et al., 2018). To minimize the potential toxicity linked to excessive hydrophobicity, one approach is to strike a balance between hydrophobic and hydrophilic residues in AMPs, optimizing their antimicrobial efficacy against pathogens while minimizing harm to mammalian cells. Drug delivery systems, ideally non-cytotoxic and non-immunogenic, offer a strategy to administer AMPs with reduced toxicity to host cells. Nanotechnology-based delivery platforms, such as the encapsulation of AMPs in liposomes or NPs, hold significant promise for improving stability and minimizing toxicity. Their small size, large surface area, and ability to target infected sites further enhance their potential for effective and selective peptide delivery (Fadaka et al., 2021).

The production cost of AMPs is notably high, primarily due to their extended amino acid sequences. The length of an AMP significantly impacts its ability to target and penetrate microbial membranes, with longer peptides often exhibiting broader-spectrum activity against diverse microorganisms. Typically, a minimum of 7–8 amino acids is required to form an amphiphilic structure. For an α-helical structure capable of traversing bacterial lipid bilayers, at least 22 amino acids are necessary, whereas β-sheet AMPs require only 8 amino acids. Consequently, developing peptide-based drugs at a reasonable cost remains challenging, mainly due to low synthesis yields and the complexities of purification processes (Sun et al., . 2014; Magana et al., 2020).

### 6.6 Antibodies as non-traditional antibacterial agents

The WHO released a list of non-traditional antibacterial in April 2021. Among them are different approaches to controlling small molecules, such as using antibodies as antibiotics. Serum antibodies were the most often used drug for treating infectious infections before developing antibiotics. To cure bacterial infectious illnesses, researchers are looking at vaccination tactics in this field once more in light of the emergence of antibiotic resistance (Domenech et al., 2018; Cavaco et al., 2022). Antibodies as medicinal agents use the immune system’s potential in humans. Human monoclonal antibodies have a longer half-life—21 days on average for IgG types—and are less likely to be eliminated by the body. They also exhibit reduced toxicity. Antibodies also have the benefit of being highly specific, which makes them a valuable therapeutic agent without interfering with the natural bacterial flora. These benefits have led to the development of several monoclonal antibodies, some of which are presently undergoing clinical studies. These antibodies have demonstrated efficacy against hospital-acquired pathogens like *S. aureus* and *P. aeruginosa*, as well as challenging-to-treat pathogens such as *A. baumannii*. A human monoclonal antibody created by Trellis Biosciences has been shown to inhibit the formation of biofilms produced by various bacteria by explicitly targeting the DNABII protein essential for biofilm development (Zurawski and McLendon, 2020; Vacca et al., 2022). A further product that Roche is developing is an *S. aureus*-specific antibody–antibiotic conjugate. The human monoclonal antibody has been engineered to attach to the exterior of *S. aureus*, thereby bringing the linked antibiotic into the immediate vicinity, augmenting the antibiotic’s efficacy (Zurawski and McLendon, 2020; Yu et al., 2023). A bi-specific antibody known as MEDI3902 was created by AstraZeneca PLC (previously known as MedImmune). This antibody is designed to specifically target a surface polysaccharide found in *P. aeruginosa*, thereby impacting its ability to form biofilms (Zurawski and McLendon, 2020). A recent phase 2 trial was carried out utilizing suvratoxumab, a human monoclonal antibody, in a double-masked, placebo-controlled manner. The study aimed to assess the efficacy of suvratoxumab in managing ventilator-associated pneumonia induced by *S. aureus* in patients within intensive care units who are on mechanical ventilators. By specifically targeting the pore-forming α-toxin, this monoclonal antibody significantly lessens its harmful effects (François et al., 2021). Since monoclonal antibodies are less likely than antibiotics to cause the rising antibiotic resistance, more studies in the field of antibodies are needed to enhance their defense against drug resistance (Yu et al., 2023).

### 6.7 Traditional medicines

Traditional medicines offer promising alternatives for combating biofilm-associated infections. Among these, andrographolide, an active compound found in the chloroform extract of *Andrographis paniculata*, holds significant potential. Commonly referred to as the “King of Bitters” in China, this plant belongs to the *Acanthaceae* family. Andrographolide (AG) has been shown to inhibit the *LasI* and *LasR* genes in *Pseudomonas aeruginosa*, which are critical quorum sensing regulators. Additionally, a 14-alpha-acyl andrographolide analog can triple the levels of RsmA, a small RNA-binding protein in *P. aeruginosa* that negatively regulates pathogenicity (Burrowes et al., . 2005; Gabrielian et al., 2002; Heurlier et al., 2004; Ma et al., 2012). Furthermore, AG can inhibit biofilm formation in *Staphylococcus aureus* by targeting the SarA system, a key regulator of biofilm development in this pathogen. In laboratory studies, AG demonstrated concentration-dependent inhibition of biofilm formation by *S. aureus MTCC* 96 (Zhang et al., 2021; Agrawal et al., 2022; Ma et al., 2023).

Another traditional plant, *Houttuynia cordata*, commonly consumed as a cold tea to promote health, also exhibits biofilm-inhibitory properties (Wu et al., 2021). Its ethanolic extract significantly prevents biofilm formation by *S. aureus*, including methicillin-resistant strains, through its combined anti-inflammatory and anti-biofilm effects. This plant has been particularly noted for its ability to reduce the risk of oral infections and treat skin abscesses (Wu et al., 2021; Chang et al., 2023). Laboratory studies suggest it inhibits biofilm formation during the early stages by depleting DNA required for biofilm assembly (Rafiq et al., 2022).

Among the various medicinal plants studied for their antimicrobial properties, *Polygonum cuspidatum* stands out as a shrub with potent biofilm-inhibiting activity. The dried stems and roots of this plant have been shown to prevent dental plaque formation by targeting *Streptococcus mutans* in the oral cavity. Although its activity lacks bactericidal properties, it effectively inhibits biofilm growth and reduces acid production by bacteria. Observations indicate that *Staphylococcus aureus* is more sensitive to this plant’s active compounds than *Pseudomonas aeruginosa* (Su et al., 2015; Ke et al., 2023).

Several medicinal plants have demonstrated synergistic effects when combined with antibiotics. For instance, naringin, a compound extracted from grapefruit peels, enhances the efficacy of ciprofloxacin and tetracycline against biofilms formed by *P. aeruginosa*. Approximately 75% of such combinations exhibit synergistic activity, underscoring the potential of integrating natural remedies with conventional antibiotics to overcome resistant biofilms (Dey et al., 2020; Zhang et al., 2020).

Despite their promising potential, the use of medicinal plants faces several challenges. Herbal medicines often contain complex compounds that may lead to unforeseen side effects, limiting their application. Standardizing formulations and identifying active components remain critical areas for further research. Nevertheless, combining natural remedies with antibiotics could offer a promising strategy to address biofilm-related infections and antibiotic resistance (Zhang et al., 2020; Vaou et al., 2021).

The following section delves into the TA system and its potential role in non-antibiotic therapeutic strategies.

### 6.8 Toxin-antitoxin system

Toxin-antitoxin (TA) systems are compact genetic units prevalent in the genomes of bacteria and archaea (Qiu et al., 2022). These systems have garnered significant attention as an alternative approach to combating antibiotic resistance. A typical TA system consists of two closely linked genes: one encodes a toxic protein that disrupts essential cellular processes, while the other encodes an antitoxin, either a protein or RNA, that neutralizes the toxin’s activity. The antitoxin is generally produced in excess to ensure the toxin remains inactive under normal conditions. However, if the antitoxin is degraded, often by cellular proteases, the toxin is released, leading to a bacteriostatic or bactericidal effect that can destroy bacterial cells (Harms et al., 2018; Równicki et al., 2020). TA systems are classified into seven types based on the mechanism of antitoxin action and their molecular nature, with type II systems—encoding protein-based antitoxins—being the most studied and prevalent. These systems are organized in operons, and their expression is tightly regulated by toxin-antitoxin complexes. This regulation ensures a balance between toxin and antitoxin levels to maintain cellular homeostasis or induce cell death under specific conditions (Yamaguchi and Inouye, 2011; Coussens and Daines, 2016; Harms et al., 2018; Równicki et al., 2020; Gu et al., 2021). TA systems are widely distributed and abundant in both Gram-positive and Gram-negative bacteria, often residing on chromosomes or plasmids. Their dual ability to modulate bacterial survival and target specific cells highlights their potential for addressing antibiotic resistance and controlling pathogenic bacteria (Pandey and Gerdes, 2005; Qiu et al., 2022).

Studies indicate that TA systems play critical roles in various biological processes, particularly in enabling bacteria to survive under stressful conditions by limiting their metabolic activity. As mentioned, TA system toxins can function as molecular “time bombs.” Compounds that artificially activate these toxins present a promising avenue for developing novel antimicrobial agents as alternatives to traditional antibiotics (Równicki et al., 2020; Sonika et al., 2023). Initially, the TA locus was identified in bacterial plasmids. However, it is now recognized as an integral component of the mobilome—a collection of genetic elements often subject to horizontal transfer. Given the significance of type II TA systems in infection management, extensive research has focused on their distribution and roles in clinically relevant pathogenic bacteria, including *Escherichia coli*, *Mycobacterium tuberculosis*, *Neisseria gonorrhoeae*, *Streptococcus* spp., *Burkholderia* spp., and ESKAPE pathogens (Fernández-García et al., 2016; Harms et al., 2018; Kang et al., 2018).

For instance, *Mycobacterium tuberculosis* has garnered attention due to its abundance of TA systems, which are closely linked to adaptive responses to host-induced stress and drug treatments. In *Streptococcus suis*, the type II TA system (Xress-MNTss) has been found to play a significant role in antibiotic resistance and pathogenicity by regulating drug-resistance genes and auto-regulation mechanisms (Bordes and Genevaux, 2021; Gu et al., 2021).

TA systems are intricately connected to bacterial persistence, as they can form regulatory cascades that influence persistence-related pathways. If toxin activity within TA systems can be effectively controlled, bacterial persistence could also be mitigated. This approach would render dormant cells susceptible to antibiotic treatment. Conversely, increasing toxin levels extends bacterial persistence. Developing strategies to deactivate toxins and activate antitoxins could potentially “wake up” persistent cells, making them more sensitive to antibiotics. However, the ubiquity and high prevalence of TA systems across numerous bacterial strains pose a significant challenge in achieving this goal (Fernández-García et al., 2016; Jurėnas et al., 2022; Sonika et al., 2023).

**Table 3** summarizes the key advantages, limitations, and implementation challenges of the non-antibiotic therapies discussed in this review. This comparison aims to provide a concise perspective on their potential and feasibility for clinical application.

**Table 3:** Comparison of non-antibiotic therapies.

Therapy	Advantages	Limitations	Implementation challenges	References
Bacteriophages	High specificity; effective against MDR bacteria	Development of phage resistance; immune reactions	Regulatory hurdles; production scalability	(Anomaly, 2020; Danis-Wlodarczyk et al., 2021; Ssekatawa et al., 2021)
Probiotics, Postbiotics, and Synbiotics	Restores gut microbiota; reduces pathogen colonization; enhances immune response	Variable efficacy; strain specificity	Standardization of formulations; large-scale validation	(Chaudhari and Dwivedi, 2022; Al-Habsi et al., 2024; Liang et al., 2024)
FMT	Restores gut microbiota balance; effective against *Clostridioides difficile* infections	Risk of infection transmission; donor variability	Regulatory oversight; development of standardized protocols	(Park and Seo, 2021; Cheng and Fischer, 2023; Thanush and Venkatesh, 2023)
NPs	Broad-spectrum activity; ability to penetrate biofilms	Potential toxicity; stability issues	Large-scale production; regulatory compliance	(Baig et al., 2021; Chen et al., 2022; Franco et al., 2022; Ozdal and Gurkok, 2022)
AMPs	Low resistance potential; broad-spectrum efficacy	Costly synthesis; potential cytotoxicity	Ensuring stability; effective delivery mechanisms	(Boparai and Sharma, 2020; Erdem Büyükkiraz and Kesmen, 2022; Bucataru and Ciobanasu, 2024)
TA Systems	Induces bacterial self-destruction; highly specific	Complexity of activation pathways; bacterial diversity	Development of reliable activators; understanding bacterial strain variations	(Równicki et al., 2020; Kamruzzaman et al., 2021)
Traditional Medicines	Widely available; potential for synergistic effects	Limited scientific validation; variability in formulations	Standardization and identification of active components	(Ma et al., 2012; Dey et al., 2020; Bittner Fialová et al., 2021)
Antibodies	Highly specific; adaptable to different targets	High cost; potential immune reactions	Scalability of production; ensuring consistent efficacy	(Zurawski and McLendon, 2020; Cavaco et al., 2022)

## 7 Conclusion

Although antibiotics have been the cornerstone of bacterial infection treatment, the escalating rates of antibiotic resistance are ushering in an era where conventional therapies may become increasingly ineffective. The golden age of antibiotics is widely regarded as over, and without effective alternatives, even common infections could soon become incurable. The emergence of drug-resistant microorganisms, driven by human activities and industrial livestock production, has created profound public health and environmental challenges. Among the major contributors to antibiotic resistance are biofilms, which play a critical role in chronic diseases, refractory wounds, and other severe conditions, often linked to the overuse and misuse of antibiotics.

In response to these challenges, non-antibiotic therapies have emerged as a promising frontier for addressing antimicrobial resistance. Strategies such as bacteriophages, probiotics, postbiotics, synbiotics, FMT, NPs, antibodies, AMPs, TA systems, and natural medicines are being actively explored. Among these, bacteriophages have garnered significant attention due to their targeting and selective targeting of specific bacteria selectively. This approach is auspicious, as it offers precision that antibiotics lack, potentially minimizing off-target effects and preserving the host microbiome. Additionally, clinical trials and case studies have provided encouraging evidence for the efficacy of these alternative approaches in various contexts, including combating biofilm-associated infections.

Despite these advances, several key research gaps remain. For these novel therapies to become mainstream, several key areas require further development. First, comprehensive clinical trials are essential to validate the safety, efficacy, and scalability of these therapies across diverse patient populations and infection types. For example, while bacteriophages show great promise, their use requires a tailored approach due to the specificity of phage-host interactions, necessitating further research into personalized treatment protocols. Similarly, the therapeutic potential of AMPs and nanoparticles needs rigorous investigation to address concerns about toxicity, stability, and delivery mechanisms.

Second, integrating non-antibiotic therapies into existing clinical practices demands a systematic approach. This includes developing standardized guidelines for their use, understanding their interactions with conventional antibiotics, and assessing the feasibility of combination therapies. Combining these novel interventions with existing antibiotics could enhance therapeutic outcomes, potentially reducing treatment durations and minimizing the emergence of resistance. Finally, there is a pressing need for policy and regulatory frameworks to facilitate the adoption of these therapies. Policymakers and researchers must collaborate to establish clear pathways for approval, production, and distribution. Furthermore, public health initiatives to promote awareness and education about these alternatives will be critical for their acceptance among healthcare providers and patients. Looking ahead, the field must prioritize interdisciplinary collaboration to address these challenges. Advances in genomics, bioinformatics, and synthetic biology could further optimize non-antibiotic therapies, making them more effective and accessible.

In conclusion, while overcoming antibiotic resistance is fraught with challenges, the emergence of non-antibiotic therapies offers a glimmer of hope. Researchers and clinicians must focus on advancing these therapies through robust clinical trials, integrating them into clinical practice, and advocating for supportive policies.

## Footnotes

1 2020 antibacterial agents in clinical and preclinical development: an overview and analysis. Available online at: https://www.who.int/publications/i/item/9789240021303 (Accessed Oct 12, 2021).

2 Available online at: https://www.who.int/news/item/27-02-2017-who-publishes-list-of-bacteria-for-which-new-antibiotics-are-urgently-needed.

## References

[B1] Adewole D. Kim I. Nyachoti A. S. (2016). Gut health of pigs: challenge models and response criteria with a critical analysis of the effectiveness of selected feed additives—a review 29 (7), 909. doi: 10.5713/ajas.15.0795 PMC4932585 26954144

[B2] Aeschlimann J. R. Therapy D. (2003). The role of multidrug efflux pumps in the antibiotic resistance of Pseudomonas aeruginosa and other gram-negative bacteria: insights from the Society of Infectious Diseases Pharmacists 23 (7), 916–924. doi: 10.1592/phco.23.7.916.32722 12885104

[B3] Afkhami H. Rezaei N. Karimi P. Hosseini S. Ahmadi R. Mohammadi T.. (2024). Converging frontiers in cancer treatment: the role of nanomaterials, mesenchymal stem cells, and microbial agents—challenges and limitations 15 (1), 1–35. doi: 10.1007/s12672-024-01590-0 PMC11662135 39707033

[B4] Agrawal P. Nair F. Pharmacology C. (2022). An insight into the pharmacological and analytical potential of Andrographolide 36 (4), 586–600. doi: 10.1111/fcp.12757 35001431

[B5] Alder K. D. Smith K. Johnson L. Brown M. Davis R. Wilson T.. (2020). Intracellular Staphylococcus aureus in bone and joint infections: a mechanism of disease recurrence, inflammation, and bone and cartilage destruction 141, 115568. doi: 10.1016/j.bone.2020.115568 32745687

[B6] Al-Habsi N. Al-Mansoori B. Al-Zaabi C. Al-Harthy D. Al-Rahbi E. Al-Saadi F.. (2024). health benefits of prebiotics, probiotics, synbiotics, and postbiotics 16 (22), 3955. doi: 10.3390/nu16223955 PMC11597603 39599742

[B7] Allen H. K. (2014). Antibiotic resistance gene discovery in food-producing animals 19, 25–29. doi: 10.1016/j.mib.2014.06.001 24994584

[B8] Alonso A. Rodriguez B. Fernandez C. Garcia D. Martinez E. Lopez F.. (2004). Overexpression of the multidrug efflux pump SmeDEF impairs Stenotrophomonas maltophilia physiology 53, 432–434. doi: 10.1093/jac/dkh074 14739147

[B9] Al-Ouqaili M. T. J. (2018). Biofilm antimicrobial susceptibility pattern for selected antimicrobial agents against planktonic and sessile cells of clinical isolates of staphylococci using MICs, BICs and MBECs. Journal of Applied Microbiology 12 (04), 123–135. doi: 10.1016/j.micpath.2018.03.012

[B10] Alprol A. E. Thompson Y. Anderson Z. Bennett A. Carter B. Edwards C.. (2023). Advances in green synthesis of metal oxide nanoparticles by marine algae for wastewater treatment by adsorption and photocatalysis techniques 13, 888. doi: 10.3390/catal13050888

[B11] Amato S. M. Orman M. A. Brynildsen M. C. (2013). Metabolic control of persister formation in Escherichia coli 50, 475–487. doi: 10.1016/j.molcel.2013.04.002 23665232

[B12] An Q. Lin R. Yang Q. Wang C. Wang D. J.J.G.A.R. (2023). Evaluation of genetic mutations associated with phenotypic resistance to fluoroquinolones, bedaquiline, and linezolid in clinical Mycobacterium tuberculosis: A systematic review and meta-analysis 34, 214–226. doi: 10.1016/j.jgar.2023.05.001 37172764

[B13] Andrä J. Berninghausen O. Leippe M. (2001). Cecropins, antibacterial peptides from insects and mammals, are potently fungicidal against Candida albicans. Med. Microbiol. Immunol. 189 (3), 169–173. doi: 10.1007/s430-001-8025-x 11388616

[B14] Anomaly J. J. (2020). The future of phage: Ethical challenges of using phage therapy to treat bacterial infections 13 (1), 82–88. doi: 10.1093/phe/phaa003 PMC7392637 32760449

[B15] Arer V. Kar I. G. (2023). Biochemical exploration of β-lactamase inhibitors 13, 1060736. doi: 10.3389/fgene.2022.1060736 PMC9888030 36733944

[B16] Arumugam A. Karthikeyan C. Hameed A. S.H. Gopinath K. Gowri S. Karthika V. J.M.S.. (2015). Synthesis of cerium oxide nanoparticles using Gloriosa superba L. leaf extract and their structural, optical and antibacterial properties 49, 408–415. doi: 10.1016/j.biotechadv.2015.04.002 25686966

[B17] Arumugam S. N. Manohar P. Sukumaran S. Sadagopan S. Loh B. Leptihn S.. (2022). Antibacterial efficacy of lytic phages against multidrug-resistant Pseudomonas aeruginosa infections in bacteraemia mice models 22 (1), 187. doi: 10.1186/s12866-022-02603-0 PMC9340724 35909125

[B18] Askari P. Namaei M. H. Ghazvini K. Hosseini M. J.B.P. (2021). *In vitro* and *in vivo* toxicity and antibacterial efficacy of melittin against clinical extensively drug-resistant bacteria 22, 1–12. doi: 10.1186/s40360-021-00503-z PMC8281584 34261542

[B19] Assa F. Jafarizadeh-Malmiri H. Ajamein H. Anarjan N. Vaghari H. Sayyar Z.. (2016). A biotechnological perspective on the application of iron oxide nanoparticles 9, 2203–2225. doi: 10.1007/s12274-016-1131-9

[B20] Azam A. Ahmed A. S. Oves M. Khan M. S. Habib S. S. Memic A. J.I. (2012). Antimicrobial activity of metal oxide nanoparticles against Gram-positive and Gram-negative bacteria: a comparative study. Journal of Antimicrobial Chemotherapy 67, 6003–6009. doi: 10.2147/IJN.S35347 PMC3519005 23233805

[B21] Azucena E. Mobashery R. U. (2001). Aminoglycoside-modifying enzymes: mechanisms of catalytic processes and inhibition 4 (2), 106–117. doi: 10.1054/drup.2001.0197 11512519

[B22] Bach H. (2018). A New Era without Antibiotics. Technology, Science, and Culture: A Global Vision. (New York, NY, USA: Springer), 1.

[B23] Baig N. Kammakakam I. Falath A. (2021). Nanomaterials: A review of synthesis methods, properties, recent progress, and challenges 2, 1821–1871. doi: 10.1039/D0MA00807A

[B24] Baker S. Olga P. Tatiana R. Nadezhda P. Tatyana G. Tatyana R.. (2020). Phyto-nano-hybrids of Ag-CuO particles for antibacterial activity against drug-resistant pathogens. J. Genet. Eng. Biotechnol. 18 (1), 53. doi: 10.1186/s43141-020-00068-0 32955647 PMC7505910

[B25] Bakken J. S. Borody T. Brandt L. J. Brill J. V. Demarco D. C. Franzos M. A.. (2011). Treating Clostridium difficile infection with fecal microbiota transplantation 9 (12), 1044–1049. doi: 10.1016/j.cgh.2011.08.014 PMC3223289 21871249

[B26] Baptista P. V. McCusker M. P. Carvalho A. Ferreira D. A. Mohan N. M. Martins M.. (2018). Nano-strategies to fight multidrug resistant bacteria—”A Battle of the Titans. Front. Microbiol. 9, 1441. doi: 10.3389/fmicb.2018.01441 30013539 PMC6036605

[B27] Barbosa C. Trebosc V. Kemmer C. Rosenstiel P. Beardmore R. Schulenburg H.. (2017). Alternative evolutionary paths to bacterial antibiotic resistance cause distinct collateral effects. Mol. Biol. Evol. 34 (9), 2229–2244. doi: 10.1093/molbev/msx158 28541480 PMC5850482

[B28] Barbu E. M. Cady K. C. Hubby P. I. B. (2016). Phage therapy in the era of synthetic biology 8 (10), a023879. doi: 10.1101/cshperspect.a023879 PMC5046696 27481531

[B29] Barros J. Melo L. D. Poeta P. Igrejas G. Ferraz M. P. Azeredo J.. (2019). Lytic bacteriophages against multidrug-resistant Staphylococcus aureus, Enterococcus faecalis and Escherichia coli isolates from orthopaedic implant-associated infections 54 (3), 329–337. doi: 10.1016/j.ijantimicag.2019.06.007 31229670

[B30] Baryshnikova N. V. Ilina A. S. Ermolenko E. I. Uspenskiy Y. P. Suvorov A. N.J.W.J.C.C.. (2023). Probiotics and autoprobiotics for treatment of Helicobacter pylori infection 11 (20), 4740. doi: 10.12998/wjcc.v11.i20.4740 PMC10424037 37583996

[B31] Basak S. Singh P. Rajurkar O. P. (2016). Multidrug resistant and extensively drug resistant bacteria: a study 2016 (1), 4065603. doi: 10.1155/2016/4065603 PMC4749793 26942013

[B32] Bates J. J. (1997). Epidemiology of vancomycin-resistant enterococci in the community and the relevance of farm animals to human infection 37 (2), 89–101. doi: 10.1016/S0195-6701(97)90179-1 9364258

[B33] Battipaglia G. Malard F. Rubio M. T. Ruggeri A. Mamez A. C. Brissot E.. (2019). Fecal microbiota transplantation before or after allogeneic hematopoietic transplantation in patients with hematologic Malignancies carrying multidrug-resistance bacteria 104 (8), 1682. doi: 10.3324/haematol.2018.198549 PMC6669143 30733264

[B34] Bebrone C. J. (2007). Metallo-β-lactamases (classification, activity, genetic organization, structure, zinc coordination) and their superfamily 74 (12), 1686–1701. doi: 10.1016/j.bcp.2007.05.021 17597585

[B35] Bednarska N. G. Wren B. W. Willcocks S. J. (2017). The importance of the glycosylation of antimicrobial peptides: Natural and synthetic approaches. Drug Discovery Today 22 (6), 919–926. doi: 10.1016/j.drudis.2017.02.001 28212948

[B36] Bhardwaj S. Choudhary M. L. Jadhav S. Vipat V. Ghuge R. Salvi S.. (2022). A retrospective analysis of respiratory virus transmission before and during the COVID-19 pandemic in Pune the western region of India 10, 936634. doi: 10.3389/fpubh.2022.936634 PMC9494283 36159243

[B37] Bhargava P. Collins C. M. (2015). Boosting bacterial metabolism to combat antibiotic resistance 21 (2), 154–155. doi: 10.1016/j.cmet.2015.01.012 25651168

[B38] Bin Hafeez A. Jiang X. Bergen P. J. Zhu Y. J.I. (2021). Antimicrobial peptides: an update on classifications and databases 22 (21), 11691. doi: 10.3390/ijms222111691 PMC8583803 34769122

[B39] Bittner Fialová S. Rendeková K. Mučaji P. Nagy M. Slobodníková L. J.I.J.M.S. (2021). Antibacterial activity of medicinal plants and their constituents in the context of skin and wound infections, considering European legislation and folk medicine—a review 22 (19), 10746. doi: 10.1016/j.foodchem.2021.129876 PMC8509446 34639087

[B40] Blasco L. Ambroa A. Trastoy R. Bleriot I. Moscoso M. Fernández-Garcia L.. (2020). *In vitro* and *in vivo* efficacy of combinations of colistin and different endolysins against clinical strains of multi-drug resistant pathogens 10 (1), 7163. doi: 10.1038/s41598-020-64145-7 PMC7188820 32346029

[B41] Boparai J. K. Sharma P. K. (2020). Mini review on antimicrobial peptides, sources, mechanism and recent applications. Protein Pept. Lett. 27 (1), 4–16. doi: 10.2174/18755305MTAwENDE80 31438824 PMC6978648

[B42] Bordeleau E. Stogios P. Evdokimova E. Koteva K. Savchenko A. Wright G. (2021). ApmA is a unique aminoglycoside antibiotic acetyltransferase that inactivates apramycin. mBio 12, e02705–e02720. doi: 10.1128/mBio.02705-20 33563840 PMC7885111

[B43] Bordes P. Genevaux B. (2021). Control of toxin-antitoxin systems by proteases in Mycobacterium tuberculosis 8, 691399. doi: 10.3389/fmolb.2021.691399 PMC8165232 34079824

[B44] Boulekbache A. Maldonado F. Kavafian R. Ferry T. Bourguignon L. Goutelle S. (2024). Comparison of daptomycin and glycopeptide efficacy and safety for the treatment of Gram-positive infections: a systematic review and meta-analysis 79 (4), 712–721. doi: 10.1093/jac/dkae026 38323372

[B45] Boumghar-Bourtchai L.. (2008). Macrolide-resistant shigella sonnei 14 (8), 1297. doi: 10.3201/eid1408.080147 PMC2600399 18680661

[B46] Bowman D. D’Silva J. Van Calster R. (2010). Defining nanomaterials for the purpose of regulation within the European Union 1 (1), 115–122. doi: 10.1017/S1867299X00000209

[B47] Bucataru C. Ciobanasu R. (2024). Antimicrobial peptides: Opportunities and challenges in overcoming resistance. Future Microbiology. 127822. doi: 10.1016/j.micres.2024.127822 38986182

[B48] Bucław M. J. J. (2016). A. Nutrition, The use of inulin in poultry feeding: a review 100 (6), 1015–1022. doi: 10.1016/j.apsoil.2016.08.012 27079815

[B49] Burrowes E. Abbas A. O'Neill A. Adams C. O'Gara F. J.R. (. 2005). Characterisation of the regulatory RNA RsmB from Pseudomonas aeruginosa PAO1 156 (1), 7–16. doi: 10.1128/AAC.49.8.3187-3194.2005 15636743

[B50] Caflisch K. M. Suh G. A. Patel R. (2019). Biological challenges of phage therapy and proposed solutions: a literature review. Expert Rev. anti-infective Ther. 17 (12), 1011–1041. doi: 10.1080/14787210.2019.1694905 PMC6919273 31735090

[B51] Cao F. Wang X. Wang L. Li Z. Che J. Wang L.. (2015). Evaluation of the efficacy of a bacteriophage in the treatment of pneumonia induced by multidrug resistance Klebsiella pneumoniae in mice. Environmental Science & Technology 2015, 4567–4579. doi: 10.1155/2015/752930 PMC4387947 25879036

[B52] Capparelli R. Nocerino N. Lanzetta R. Silipo A. Amoresano A. Giangrande C.. (2007). Experimental phage therapy against Staphylococcus aureus in mice 51 (8), 2765–2773. doi: 10.1128/AAC.01513-06 PMC1932491 17517843

[B53] Capparelli R. Parlato M. Borriello G. Salvatore P. Iannelli D. J.A. (2010). Bacteriophage-resistant Staphylococcus aureus mutant confers broad immunity against staphylococcal infection in mice 5 (7), e11720. doi: 10.1371/journal.pone.0011720 PMC2908692 20661301

[B54] Cavaco M. Castanho M. A. Neves I. M. (2022). The use of antibody-antibiotic conjugates to fight bacterial infections 13, 835677. doi: 10.3389/fmicb.2022.835677 PMC8940529 35330773

[B55] Caveney N. A. Caballero G. Voedts H. Niciforovic A. Worrall L. J. Vuckovic M.. (2019). Structural insight into YcbB-mediated beta-lactam resistance in Escherichia coli 10 (1), 1849. doi: 10.1038/s41467-019-09507-0 PMC6478713 31015395

[B56] Cerveny K. E. DePaola A. Duckworth D. H. Gulig P. A. (2002). Phage therapy of local and systemic disease caused by Vibrio vulnificus in iron-dextran-treated mice. Infection Immun. 70 (11), 6251–6262. doi: 10.1128/IAI.70.11.6251-6262.2002 PMC130292 12379704

[B57] Chang Y. Xia S. Fei P. Feng H. Fan F. Liu Y. (2023). Houttuynia cordata Thunb. crude extract inactivates Cronobacter sakazakii: Antibacterial components, antibacterial mechanism, and application as a natural disinfectant 145, 109467.

[B58] Chantziaras I. Boyen F. Callens B. Dewulf J. J.J.A.C. (2014). Correlation between veterinary antimicrobial use and antimicrobial resistance in food-producing animals: a report on seven countries 69 (3), 827–834. doi: 10.1093/jac/dkt443 24216767

[B59] Chaudhari A. Dwivedi M. K. (2022). “The concept of probiotics, prebiotics, postbiotics, synbiotics, nutribiotics, and pharmabiotics,” in Probiotics in the prevention and management of human diseases (New Delhi, India: Elsevier), 1–11.

[B60] Chavanet P. Fournel I. Bourredjem A. Piroth L. Blot M. Sixt T.. (2023). Addition of daptomycin for the treatment of pneumococcal meningitis: protocol for the AddaMAP study 13 (7), e073032. doi: 10.1136/bmjopen-2023-073032 PMC10373719 37491088

[B61] Chen H. Zhang J. He Y. Lv Z. Liang Z. Chen J.. (2022). Exploring the role of Staphylococcus aureus in inflammatory diseases 14 (7), 464. doi: 10.3390/toxins14070464 PMC9318596 35878202

[B62] Chen Q. Riviere J. E. Lin Z. J.W.I.R.N. (2022). Toxicokinetics, dose–response, and risk assessment of nanomaterials: Methodology, challenges, and future perspectives 14 (6), e1808. doi: 10.1002/wnan.v14.6 PMC9699155 36416026

[B63] Chen X. Liu S. Fang J. Zheng S. Wang Z. Jiao Y.. (2022). Peptides isolated from amphibian skin secretions with emphasis on antimicrobial peptides 14 (10), 722. doi: 10.3390/toxins14100722 PMC9607450 36287990

[B64] Chen N. Jiang C. (2023). Antimicrobial peptides: Structure, mechanism, and modification 255, 115377. doi: 10.1016/j.ejmech.2023.115377 37099837

[B65] Chen C. H. Lu J. A. (2020). Development and challenges of antimicrobial peptides for therapeutic applications 9, 24. doi: 10.3390/antibiotics9010024 PMC7168295 31941022

[B66] Cheng Y. W. Fischer I. C. (2023). R. Surgery, Fecal microbiota transplantation 36 (02), 151–156. doi: 10.1021/acs.jafc.3c01234 PMC9946715 36844708

[B67] Conlon J. M. Sonnevend A. (2011). Clinical applications of amphibian antimicrobial peptides. J. Med. Sci. 4 (2), 62–72. doi: 10.1128/AAC.00123-11

[B68] Control, C.F.D (2002). “Antimicrobial resistance: a growing threat to public health,” in Atlanta: division of healthcare quality promotion (Atlanta, GA, USA: National Center for Infectious Diseases).

[B69] Coussens N. P. Daines D. A. (2016). Wake me when it’s over–bacterial toxin–antitoxin proteins and induced dormancy. Exp. Biol. Med. 241 (12), 1332–1342. doi: 10.1177/1535370216651938 PMC4909563 27216598

[B70] D’Costa V. M. McGrann K. M. Hughes D. W. Wright G. D.J.S. (2006). Sampling the antibiotic resistome 311 (5759), 374–377. doi: 10.1126/science.1120800 16424339

[B71] Dadfar S. M. Roemhild K. Drude N. I. von Stillfried S. Knüchel R. Kiessling F.. (2019). Iron oxide nanoparticles: Diagnostic, therapeutic and theranostic applications 138, 302–325. doi: 10.1016/j.addr.2019.01.005 PMC7115878 30639256

[B72] Danis-Wlodarczyk K. Dąbrowska K. Abedon M. B. (2021). Phage therapy: the pharmacology of antibacterial viruses 40 (1), 81–164. doi: 10.21775/cimb.040.081 32503951

[B73] Darby E. M. Trampari E. Siasat P. Gaya M. S. Alav I. Webber M. A.. (2023). Molecular mechanisms of antibiotic resistance revisited 21 (5), 280–295. doi: 10.1038/s41579-022-00820-y 36411397

[B74] Davies O. L. Bennett S. (2017). WHO publishes list of bacteria for which new antibiotics are urgently needed. WHO Newsletters.

[B75] de Lacerda Coriolano D. de Souza J. B. Bueno E. V. Medeiros S. M.F.R.S. Cavalcanti I. D.L. Cavalcanti I. M.F.J.B.J.M. (2021). Antibacterial and antibiofilm potential of silver nanoparticles against antibiotic-sensitive and multidrug-resistant Pseudomonas aeruginosa strains 52, 267–278. doi: 10.1007/s42770-020-00406-x PMC7966632 33231865

[B76] Del Giudice P.J.A.D.V. (2020). Skin infections caused by Staphylococcus aureus. Frontiers in Microbiology 100, 1–15. doi: 10.2340/00015555-3466 PMC9128951 32207539

[B77] De Pascale G. Wright J. C. (2010). Antibiotic resistance by enzyme inactivation: from mechanisms to solutions 11 (10), 1325–1334. doi: 10.1002/cbic.201000067 20564281

[B78] Dey P. Parai D. Banerjee M. Hossain S. T. Mukherjee S. K. (2020). Naringin sensitizes the antibiofilm effect of ciprofloxacin and tetracycline against Pseudomonas aeruginosa biofilm. Int. J. Med. Microbiol. 310 (3), 151410. doi: 10.1016/j.ijmm.2020.151410 32057619

[B79] Diana A. Boyle L. A. Leonard F. C. Carroll C. Sheehan E. Murphy D.. (2019). Removing prophylactic antibiotics from pig feed: how does it affect their performance and health? 15, 1–8. doi: 10.1186/s12917-019-1808-x PMC6390319 30808361

[B80] Diene S. M. Bruder N. Raoult D. Rolain J.-M. J.I. (2011). Real-time PCR assay allows detection of the New Delhi metallo-β-lactamase (NDM-1)-encoding gene in France 37 (6), 544–546. doi: 10.1016/j.ijantimicag.2011.02.006 21497063

[B81] Dijksteel G. S. Ulrich M. M. Middelkoop E. Boekema B. K.J.F. (2021). Lessons learned from clinical trials using antimicrobial peptides (AMPs) 12, 616979. doi: 10.3389/fmicb.2021.616979 PMC7937881 33692766

[B82] Ding B. Li Q. Guo M. Dong K. Zhang Y. Guo X.. (2018). Prevention of dermal abscess formation caused by Staphylococcus aureus using phage JD007 in nude mice 9, 1553. doi: 10.3389/fmicb.2018.01553 PMC6064926 30083139

[B83] Domenech M. Sempere J. de Miguel S. Yuste J. J.F. (2018). Combination of antibodies and antibiotics as a promising strategy against multidrug-resistant pathogens of the respiratory tract 9, 2700. doi: 10.3389/fimmu.2018.02700 PMC6256034 30515172

[B84] Duc H. M. Zhang Y. Hoang S. M. Masuda Y. Honjoh K.-I. Miyamoto T. J.A. (2023). The use of phage cocktail and various antibacterial agents in combination to prevent the emergence of phage resistance 12 (6), 1077. doi: 10.3390/antibiotics12061077 PMC10295611 37370397

[B85] Ebrahimi S. Sisakhtpour B. Mirzaei A. Karbasizadeh V. Moghim S. J.G.R. (2021). Efficacy of isolated bacteriophage against biofilm embedded colistin-resistant Acinetobacter baumannii 22, 100984. doi: 10.1016/j.genrep.2020.100984

[B86] Egorov A. Ulyashova M. Rubtsova A. N. (2018). Bacterial enzymes and antibiotic resistance 10 (39), 33–48. doi: 10.32607/20758251-2018-10-4-33-48 PMC6351036 30713760

[B87] Eiseman B. Silen W. GS B. AJ K. J.S.( (1958). Fecal enema as an adjunct in the treatment of pseudomembranous enterocolitis 44 (5), 854–859. doi: 10.1016/0006-291X(58)90074-8 13592638

[B88] Erdem Büyükkiraz M. Kesmen M. (2022). Antimicrobial peptides (AMPs): A promising class of antimicrobial compounds 132 (3), 1573–1596. doi: 10.1111/jam.15314 34606679

[B89] Fadaka A. O. Sibuyi N. R.S. Madiehe A. M. Meyer M. J.P. (2021). Nanotechnology-based delivery systems for antimicrobial peptides 13 (11), 1795. doi: 10.3390/pharmaceutics13111795 PMC8620809 34834210

[B90] Farias I. A. P. Santos C. L. D. Sampaio B. R. I. (2018). Antimicrobial activity of cerium oxide nanoparticles on opportunistic microorganisms: a systematic review 2018 (1), 1923606. doi: 10.1155/2018/1923606 PMC5827881 29607315

[B91] Fayez M. S. Hakim T. A. Agwa M. M. Abdelmoteleb M. Aly R. G. Montaser N. N.. (2021). Topically applied bacteriophage to control multi-drug resistant Klebsiella pneumoniae infected wound in a rat model 10 (9), 1048. doi: 10.3390/antibiotics10091048 PMC8470685 34572629

[B92] Ferdous Z. Nemmar S. (2020). Health impact of silver nanoparticles: a review of the biodistribution and toxicity following various routes of exposure 21 (7), 2375. doi: 10.3390/ijms21072375 PMC7177798 32235542

[B93] Fernández L. Hancock M. R. (2012). Adaptive and mutational resistance: role of porins and efflux pumps in drug resistance 25 (4), 661–681. doi: 10.1128/CMR.00043-12 PMC3485749 23034325

[B94] Fernández-Alonso M. Aguirre Camorlinga A. Messiah S. E. Marroquin E. J.J.M.M. (2022). Effect of adding probiotics to an antibiotic intervention on the human gut microbial diversity and composition: A systematic review 71 (11), 001625. doi: 10.1099/jmm.0.001625 36382780

[B95] Fernández-García L. Blasco L. Lopez M. Bou G. García-Contreras R. Wood T.. (2016). Toxin-antitoxin systems in clinical pathogens. Toxins 8 (7), 227. doi: 10.3390/toxins8070227 27447671 PMC4963858

[B96] Fernández Llamas L. Cima-Cabal M. D. Duarte A. C. Rodríguez González A. García Suárez M. P. García-Suárez M. M. (2020). Developing diagnostic and therapeutic approaches to bacterial infections for a new era: implications of globalization. (Cham, Switzerland: Springer Nature). 10.3390/antibiotics9120916 PMC7765786 33339391

[B97] Ferry T. Kolenda C. Batailler C. Gustave C.-A. Lustig S. Malatray M.. (2020). Phage therapy as adjuvant to conservative surgery and antibiotics to salvage patients with relapsing S. aureus prosthetic knee infection 7, 570572. doi: 10.1016/j.envint.2020.105894 PMC7701306 33304911

[B98] Flórez-Castillo J.-M. Ropero-Vega J. L. Perullini M. Jobbágy M. J.H. (2019). Biopolymeric pellets of polyvinyl alcohol and alginate for the encapsulation of Ib-M6 peptide and its antimicrobial activity against E. coli. Antimicrobial Agents and Chemotherapy 5 (6), e01234-19. doi: 10.1016/j.jgar.2019.02.001 PMC6551476 31194071

[B99] Floss H. G. Yu C. R. (2005). Rifamycin mode of action, resistance, and biosynthesis 105 (2), 621–632. doi: 10.1021/cr030112j 15700959

[B100] Franco D. Calabrese G. Guglielmino S. P.P. Conoci S. J.M. (2022). Metal-based nanoparticles: Antibacterial mechanisms and biomedical application 10 (9), 1778. doi: 10.3390/microorganisms10091778 PMC9503339 36144380

[B101] François B. Jafri H. S. Chastre J. Sánchez-García M. Eggimann P. Dequin P.-F.. (2021). Efficacy and safety of suvratoxumab for prevention of Staphylococcus aureus ventilator-associated pneumonia (SAATELLITE): a multicentre, randomised, double-blind, placebo-controlled, parallel-group, phase 2 pilot trial 21 (9), 1313–1323. doi: 10.1016/j.cmi.2021.03.001 33894131

[B102] Freedman A. Eppes S. (1805). “Use of stool transplant to clear fecal colonization with Carbapenem-Resistant Enterobacteraciae (CRE): Proof of concept,” in Open Forum Infectious Diseases (London, UK: Oxford University Press), 2014.

[B103] Gabrielian E. Shukarian A. Goukasova G. Chandanian G. Panossian A. G. Wikman G.. (2002). A double blind, placebo-controlled study of Andrographis paniculata fixed combination Kan Jang in the treatment of acute upper respiratory tract infections including sinusitis. Phytomedicine 9 (7), 589–597. doi: 10.1078/094471102321616391 12487322

[B104] Ghimpeteanu O. M. Pogurschi E. N. Popa D. C. Dragomir N. Dragotoiu T. Mihai O. D.. (2022). Antibiotic use in livestock and residues in food—A public health threat: A review 11 (10), 1430. doi: 10.3390/foods11101430 PMC9142037 35627000

[B105] Giurazza R. Mazza M. C. Andini R. Sansone P. Pace M. C. Durante-Mangoni E. J.L. (2021). Emerging treatment options for multi-drug-resistant bacterial infections 11 (6), 519. doi: 10.3390/life11060519 PMC8229628 34204961

[B106] Golkar Z. Bagasra O. Pace D. G. (2014). Bacteriophage therapy: a potential solution for the antibiotic resistance crisis. J. Infect. Dev. Ctries 8 (2), 129–136. doi: 10.3855/jidc.3573 24518621

[B107] Gomez-Ochoa S. A. Pitton M. Valente L. G. Vesga C. D.S. Largo J. Quiroga-Centeno A. C.. (2022). Efficacy of phage therapy in preclinical models of bacterial infection: a systematic review and meta-analysis 3 (12), e956–e968. doi: 10.1016/S2666-5247(22)00288-9 36370748

[B108] Gopinath K. Karthika V. Sundaravadivelan C. Gowri S. Arumugam A. J.J.N.C. (2015). Mycogenesis of cerium oxide nanoparticles using Aspergillus Niger culture filtrate and their applications for antibacterial and larvicidal activities 5, 295–303. doi: 10.1007/s40097-015-0161-2

[B109] Grande R. Puca V. Muraro T. P. (2020). Antibiotic resistance and bacterial biofilm 30 (12), 897–900. doi: 10.1080/13543776.2020.1830060 32985275

[B110] Grant T. A. López-Pérez M. Haro-Moreno J. M. Almagro-Moreno S. J.P.G. (2023). Allelic diversity uncovers protein domains contributing to the emergence of antimicrobial resistance 19 (3), e1010490. doi: 10.1371/journal.pgen.1010490 PMC10079234 36972246

[B111] Gu Q. He P. Wang D. Ma J. Zhong X. Zhu Y.. (2021). An auto-regulating type II toxin-antitoxin system modulates drug resistance and virulence in streptococcus suis. Front. Microbiol. 12, 671706. doi: 10.3389/fmicb.2021.671706 34475853 PMC8406773

[B112] Gudkov S. V. Burmistrov D. E. Serov D. A. Rebezov M. B. Semenova A. A. Lisitsyn A. B.J.A. (2021). Do iron oxide nanoparticles have significant antibacterial properties? 10 (7), 884. doi: 10.3390/antibiotics10070884 PMC8300809 34356805

[B113] Guha S. Ferrie R. P. Ghimire J. Ventura C. R. Wu E. Sun L.. (2021). Applications and evolution of melittin, the quintessential membrane active peptide 193, 114769. doi: 10.1016/j.bcp.2021.114769 PMC9235364 34543656

[B114] Haddad Kashani H. Schmelcher M. Sabzalipoor H. Seyed Hosseini E. Moniri R. J.C. (2018). Recombinant endolysins as potential therapeutics against antibiotic-resistant Staphylococcus aureus: current status of research and novel delivery strategies. International Journal of Antimicrobial Agents 31 (1), 301–308. doi: 10.1128/cmr.00071-17 PMC5740972 29187396

[B115] Hall B. G. Salipante S. J. Barlow M. E. (2004). Independent origins of subgroup bl+ b2 and subgroup b3metallo-β-lactamases 59, 133–141. doi: 10.1007/s00239-003-2572-9 15383916

[B116] Hamburg M. A. (2012). FDA’s approach to regulation of products of nanotechnology 336 (6079), 299–300. doi: 10.1126/science.1205441 22517845

[B117] Hammerum A. M. Heuer O. E. Emborg H.-D. Bagger-Skjøt L. Jensen V. F. Rogues A.-M.. (2007). Danish integrated antimicrobial resistance monitoring and research program 13 (11), 1633. doi: 10.3201/eid1311.070421 PMC3375779 18217544

[B118] Hanson N. Harris J. Joseph L. A. Ramakrishnan K. Thompson T. (2011). EPA needs to manage nanomaterial risks more effectively. (Washington, D.C., USA: American Society for Microbiology (ASM) Press).

[B119] Harms A. Brodersen D. E. Mitarai N. Gerdes K. (2018). Toxins, targets, and triggers: an overview of toxin-antitoxin biology. Mol. Cell 70 (5), 768–784. doi: 10.1016/j.molcel.2018.01.003 29398446

[B120] Harper D. R. (2021). Technology, Therapy, Introduction to bacteriophages. (London, UK: Routledge), 3–16.

[B121] Hasdemir U. O. Chevalier J. Nordmann P. Page`s J.-M. J.J. (2004). Detection and prevalence of active drug efflux mechanism in various multidrug-resistant Klebsiella pneumoniae strains from Turkey 42 (3), 2701–2706. doi: 10.1128/JCM.42.6.2701-2706.2004 PMC427859 15184455

[B122] Henderson P. J. Maher C. Elbourne L. D. Eijkelkamp B. A. Paulsen I. T. Hassan K. A.J.C.. (2021). Physiological functions of bacterial “multidrug” efflux pumps 121 (9), 5417–5478. doi: 10.1021/acs.chemrev.0c01226 33761243

[B123] Heuer H. Schmitt H. Smalla I. M. (2011). Antibiotic resistance gene spread due to manure application on agricultural fields 14 (3), 236–243. doi: 10.1016/j.mib.2011.04.009 21546307

[B124] Heurlier K. Williams F. Heeb S. Dormond C. Pessi G. Singer D.. (2004). Positive control of swarming, rhamnolipid synthesis, and lipase production by the posttranscriptional RsmA/RsmZ system in Pseudomonas aeruginosa PAO1 186 (10), 2936–2945. doi: 10.1128/JB.186.10.2936-2945.2004 PMC400603 15126453

[B125] Hochvaldová L. Večeřová R. Kolář M. Prucek R. Kvítek L. Lapčík L.. (2022). Antibacterial nanomaterials: Upcoming hope to overcome antibiotic resistance crisis . 11 (1), 1115–1142. doi: 10.1515/ntrev-2022-0059

[B126] Høiby N. Bjarnsholt T. Givskov M. Molin S. Ciofu O. J.I. (2010). Antibiotic resistance of bacterial biofilms 35 (4), 322–332. doi: 10.1016/j.ijantimicag.2009.12.011 20149602

[B127] Holger D. J. Lev K. L. Kebriaei R. Morrisette T. Shah R. Alexander J.. (2022). Bacteriophage-antibiotic combination therapy for multidrug-resistant Pseudomonas aeruginosa: *In vitro* synergy testing 133 (3), 1636–1649. doi: 10.1111/jam.15647 35652690

[B128] Hou J. Long X. Wang X. Li L. Mao D. Luo Y.. (2023). Global trend of antimicrobial resistance in common bacterial pathogens in response to antibiotic consumption 442, 130042. doi: 10.1016/j.jhazmat.2022.130042 36182890

[B129] Hu J. Tian X. Wei T. Wu H. Lu J. Lyu M.. (2021). Anti-Helicobacter pylori activity of a Lactobacillus sp. PW-7 exopolysaccharide 10 (10), . 2453. doi: 10.1016/j.scitotenv.2021.145678 PMC8535340 34681500

[B130] Hua Y. Luo T. Yang Y. Dong D. Wang R. Wang Y.. (2018). Phage therapy as a promising new treatment for lung infection caused by carbapenem-resistant Acinetobacter baumannii in mice 8, 318476. doi: 10.3389/fmicb.2017.02659 PMC5767256 29375524

[B131] Hunashal Y. Kumar G. S. Choy M. S. D Andréa É. D. Da Silva Santiago A. Schoenle M. V.. (2023). Molecular basis of β-lactam antibiotic resistance of ESKAPE bacterium E. faecium Penicillin Binding Protein PBP5 14 (1), 4268. 10.1038/s41467-023-39966-5 PMC10352307 37460557

[B132] Hunt A. Drwiega E. Wang Y. Danziger L. J.A.J.H.-S.P. (2024). A review of fecal microbiota, live-jslm for the prevention of recurrent Clostridioides difficile infection. Nature Reviews Microbiology., zxae066. doi: 10.1093/ajhp/zxae066 38470061

[B133] Huo W. Busch L. M. Hernandez-Bird J. Hamami E. Marshall C. W. Geisinger E.. (2022). Immunosuppression broadens evolutionary pathways to drug resistance and treatment failure during Acinetobacter baumannii pneumonia in mice 7 (6), 796–809. doi: 10.1038/s41564-022-01126-8 PMC9159950 35618774

[B134] Ibrahim Khan K. S. Khan O. C. (2017). Nanoparticles: Properties, applications and toxicities 12 (7), 908–931. doi: 10.1016/j.jinf.2017.03.012

[B135] Ismail R. A. Sulaiman G. M. Abdulrahman S. A. Marzoog T. R.J.M.S. Caa E. (2015). Antibacterial activity of magnetic iron oxide nanoparticles synthesized by laser ablation in liquid 53, 286–297. doi: 10.1016/j.msec.2015.04.047 26042717

[B136] Jaber R. H. Beahm A. A. (2023). Daptomycin for the treatment of acute bacterial meningitis: A narrative review 61 (5), 106770. doi: 10.1016/j.ijantimicag.2023.106770 36870402

[B137] Jacobs M. R. (1999). Drug-resistant Streptococcus pneumoniae: rational antibiotic choices 106 (5), 19–25. doi: 10.1016/S0002-9343(98)00351-9 10348060

[B138] Jain M. Stitt G. Son L. Enioutina E. Y.J.M. (2023). Probiotics and their bioproducts: a promising approach for targeting methicillin-resistant Staphylococcus aureus and vancomycin-resistant enterococcus 11 (10), 2393. doi: 10.3390/microorganisms11102393 PMC10608974 37894051

[B139] Jang S. J. B. R. Tolc A. (2023). a major efflux pump in Gram negative bacteria: toward understanding its operation mechanism 56 (6), 326. doi: 10.5483/BMBRep.2023-0070 PMC10315565 37254571

[B140] Jia G. Han Y. An Y. Ding Y. He C. Wang X.. (2018). NRP-1 targeted and cargo-loaded exosomes facilitate simultaneous imaging and therapy of glioma *in vitro* and *in vivo* 178, 302–316. doi: 10.1016/j.biomaterials.2018.06.029 29982104

[B141] Jin D. J. Gross M. B. (1988). Mapping and sequencing of mutations in the Escherichia colirpoB gene that lead to rifampicin resistance 202 (1), 45–58. doi: 10.1016/0022-2836(88)90517-7 3050121

[B142] Johnson A. P. Woodford M. M. (2013). Global spread of antibiotic resistance: the example of New Delhi metallo-β-lactamase (NDM)-mediated carbapenem resistance 62 (4), 499–513. doi: 10.1099/jmm.0.052555-0 23329317

[B143] Jones T. W. Jun A. H. Michal J. L. Olney W. J.J.A.P. (2021). High-dose daptomycin and clinical applications 55 (11), 1363–1378. doi: 10.1016/j.vaccine.2021.05.001 PMC8573721 33535792

[B144] Jurėnas D. Fraikin N. Goormaghtigh F. Van Melderen L. J.N.R.M. (2022). Biology and evolution of bacterial toxin–antitoxin systems 20 (6), 335–350. doi: 10.1016/j.tim.2022.01.001 34975154

[B145] Kamer A. M. A. Abdelaziz A. A. Nosair A. M. Al-Madboly L. A.J.L.S. (2022). Characterization of newly isolated bacteriophage to control multi-drug resistant Pseudomonas aeruginosa colonizing incision wounds in a rat model: *in vitro* and *in vivo* approach 310, . 121085. doi: 10.1016/j.nano.2022.102567 36265569

[B146] Kamruzzaman M. Wu A. Y. Iredell J. M. (2021). Biological functions of type II toxin-antitoxin systems in bacteria 9 (6), . 1276. doi: 10.3390/microorganisms9061276 PMC8230891 34208120

[B147] Kang S.-J. Park S. J. Mishig-Ochir T. Lee B.-J. J.E.(. (2014). Antimicrobial peptides: therapeutic potentials 12 (12), 1477–1486. doi: 10.1586/14787210.2014.976613 25371141

[B148] Kang S. M.. (2018). A systematic overview of type II and III toxin-antitoxin systems with a focus on druggability. Toxins 10 (12), 515. doi: 10.3390/toxins10120515 30518070 PMC6315513

[B149] Karnwal A.. (2023). Perspectives on usage of functional nanomaterials in antimicrobial therapy for antibiotic-resistant bacterial infections 8 (15), 13492–13508. doi: 10.1021/acsomega.3c00110 PMC10116640 37091369

[B150] Kasman L. M. Porter L. D. (2022). “Bacteriophages,” in StatPearls [Internet] (Amsterdam, Netherlands: StatPearls Publishing).

[B151] Kaur S. Harjai K. Chhibber S. (2014). Bacteriophage mediated killing of Staphylococcus aureus *in vitro* on orthopaedic K wires in presence of linezolid prevents implant colonization. PloS One 9 (3), e90411. doi: 10.1371/journal.pone.0090411 24594764 PMC3940871

[B152] Ke J. Li M.-T. Xu S. Ma J. Liu M.-Y. Han Y. J.P.b.. (2023). Advances for pharmacological activities of Polygonum cuspidatum-A review 61 (1), 177–188. doi: 10.1080/13880209.2022.2158349 PMC9833411 36620922

[B153] Kebriaei R. Stamper K. C. Singh K. V. Khan A. Rice S. A. Dinh A. Q.. (2020). Mechanistic insights into the differential efficacy of daptomycin plus β-lactam combinations against daptomycin-resistant Enterococcus faecium 222 (9), 1531–1539. doi: 10.1093/infdis/jiaa319 PMC7529040 32514561

[B154] Keen E. C. (2012). Felix d’Herelle and our microbial future. Future Microbiology 7 (12), 1337–1339. doi: 10.2217/fmb.12.115 23231482

[B155] Keller M. R. Dörr M. P. (2023). Bacterial metabolism and susceptibility to cell wall-active antibiotics 83, 181–219. doi: 10.1016/bs.ampbs.2023.04.002 PMC11024984 37507159

[B156] Kelly C. R. Khoruts A. Staley C. Sadowsky M. J. Abd M. Alani M.. (2016). Effect of fecal microbiota transplantation on recurrence in multiply recurrent Clostridium difficile infection: a randomized trial 165 (9), 609–616. doi: 10.7326/M16-0271 PMC5909820 27547925

[B157] Khaledi M. Sameni F. Yahyazade S. Radandish M. Owlia P. Bagheri N.. (2022). COVID-19 and the potential of Janus Family kinase (JAK) Pathway Inhibition: as a novel treatment strategy. (Amsterdam, Netherlands: Elsevier), 2545. 10.3389/fmed.2022.961027 PMC9469902 36111104

[B158] Khan A. U. Maryam L. Zarrilli B. M. (2017). Structure, genetics and worldwide spread of New Delhi metallo-β-lactamase (NDM): a threat to public health 17, 1–12. doi: 10.1186/s12866-017-1012-8 PMC5408368 28449650

[B159] Khoruts A. Sadowsky R. G. (2016). Hepatology, Understanding the mechanisms of faecal microbiota transplantation 13 (9), 508–516. doi: 10.1038/nrgastro.2016.98 PMC5909819 27329806

[B160] Kim T.-S. Hur J.-W. Yu M.-A. Cheigh C.-I. Kim K.-N. Hwang J.-K.. (2003). Antagonism of Helicobacter pylori by bacteriocins of lactic acid bacteria 66 (1), . 3–.12. doi: 10.1016/S0168-1605(03)00101-2 12540174

[B161] Kingwell K. J. (2015). Bacteriophage therapies re-enter clinical trials 14 (8), 515. doi: 10.1038/nrd4695 26228748

[B162] Klevens R. M. Morrison M. A. Nadle J. Petit S. Gershman K. Ray S.. (2007). Invasive methicillin-resistant Staphylococcus aureus infections in the United States. Jama 298 (15), 1763–1771. doi: 10.1001/jama.298.15.1763 17940231

[B163] Konwar A. N. Hazarika S. N. Bharadwaj P. Thakur D. J.C.M. (2022). Emerging non-traditional approaches to combat antibiotic resistance 79 (11), 330. doi: 10.1007/s00284-022-03029-7 PMC9510247 36155858

[B164] Krishnamoorthy K. Veerapandian M. Zhang L.-H. Yun K. Kim S. J.J.J.I. Chemistry E. (2014). Surface chemistry of cerium oxide nanocubes: Toxicity against pathogenic bacteria and their mechanistic study 20 (5), 3513–3517. doi: 10.1016/j.jiec.2013.12.043

[B165] Kumar M. Sarma D. K. Shubham S. Kumawat M. Verma V. Nina P. B.. (2021). Futuristic non-antibiotic therapies to combat antibiotic resistance: A review 12, 609459. doi: 10.3389/fmicb.2021.609459 PMC7870489 33574807

[B166] Kumar P. Kizhakkedathu J. N. Straus J. B. (2018). Antimicrobial peptides: diversity, mechanism of action and strategies to improve the activity and biocompatibility *in vivo* 8 (1), 4. doi: 10.3390/biom8010004 PMC5871973 29351202

[B167] Kumarasamy K. K. Toleman M. A. Walsh T. R. Bagaria J. Butt F. Balakrishnan R.. (2010). Emergence of a new antibiotic resistance mechanism in India, Pakistan, and the UK: a molecular, biological, and epidemiological study 10 (9), 597–602. doi: 10.1016/S1473-3099(10)70143-2 PMC2933358 20705517

[B168] Lagadinou M. Onisor M. O. Rigas A. Musetescu D.-V. Gkentzi D. Assimakopoulos S. F.. (2020). Antimicrobial properties on non-antibiotic drugs in the era of increased bacterial resistance 9 (3), 107. doi: 10.3390/antibiotics9030107 PMC7175110 32131427

[B169] Lagrafeuille R. Miquel S. Balestrino D. Vareille-Delarbre M. Chain F. Langella P.. (2018). Opposing effect of Lactobacillus on *in vitro* Klebsiella pneumoniae in biofilm and in an *in vivo* intestinal colonisation model 9 (1), 87–100. doi: 10.3920/BM2017.0002 29022382

[B170] Lahtinen S. J. Jalonen L. Ouwehand A. C. Salminen S. J. J. I. j. o. f. m. (2007). Specific Bifidobacterium strains isolated from elderly subjects inhibit growth of Staphylococcus aureus 117 (1), 125–128. doi: 10.1016/j.ijfoodmicro.2007.02.023 17462772

[B171] Lambert P. A. (2005). Bacterial resistance to antibiotics: modified target sites 57 (10), 1471–1485. doi: 10.1016/j.addr.2005.04.003 15964098

[B172] Lamberte L. E. an Schaik I. M. (2022). Antibiotic resistance in the commensal human gut microbiota 68, 102150. doi: 10.1016/j.mib.2022.102150 35490629

[B173] Langdon A. Schwartz D. J. Bulow C. Sun X. Hink T. Reske K. A.. (2021). Microbiota restoration reduces antibiotic-resistant bacteria gut colonization in patients with recurrent Clostridioides difficile infection from the open-label PUNCH CD study 13, 1–18. doi: 10.1186/s13073-021-00843-9 PMC7888090 33593430

[B174] Larsen J. Raisen C. L. Ba X. Sadgrove N. J. Padilla-González G. F. Simmonds M. S.. (2022). Emergence of methicillin resistance predates the clinical use of antibiotics 602 (7895), 135–141. doi: 10.1016/j.antiviral.2022.105287 PMC8810379 34987223

[B175] Lawson P. A. Citron D. M. Tyrrell K. L. Finegold S. M. J. A. (2016). Reclassification of clostridium difficile as clostridioides difficile (Hall and O’Toole 1935) Prévot 1938 40, 95–99. doi: 10.1016/j.anaerobe.2016.06.008 27370902

[B176] Lebeer S. Vanderleyden J. De Keersmaecker R. M. (2010). Host interactions of probiotic bacterial surface molecules: comparison with commensals and pathogens 8 (3), 171–184. doi: 10.1038/nrmicro2297 20157338

[B177] Leclercq R. J. (2002). Mechanisms of resistance to macrolides and lincosamides: nature of the resistance elements and their clinical implications 34 (4), 482–492. doi: 10.1086/324626 11797175

[B178] Lee I. H. Cho Y. Lehrer R. I.J.I. (1997). Effects of pH and salinity on the antimicrobial properties of clavanins 65 (7), 2898–2903. doi: 10.1128/iai.65.7.2898-2903.1997 PMC175407 9199465

[B179] Lee D. H. Kim B. S. Kang S.-S. J.P.. (2020). Bacteriocin of Pediococcus acidilactici HW01 inhibits biofilm formation and virulence factor production by Pseudomonas aeruginosa 12, 73–81. doi: 10.1007/s12602-019-09623-9 31784952

[B180] Lehman S. M. Mearns G. Rankin D. Cole R. A. Smrekar F. Branston S. D.. (2019). Design and preclinical development of a phage product for the treatment of antibiotic-resistant Staphylococcus aureus infections 11 (1), 88. doi: 10.1016/j.ijantimicag.2019.02.001 PMC6356596 30669652

[B181] Li B. Kang W. Liu H. Wang Y. Yu C. Zhu X.. (2016). The antimicrobial activity of Cbf-K 16 against MRSA was enhanced by β-lactamantibiotics through cell wall non-integrity 39, 978–988. doi: 10.1016/j.foodcont.2016.05.001 27287456

[B182] Li X. He Y. Wang Z. Wei J. Hu T. Si J.. (2021). A combination therapy of Phages and Antibiotics: Two is better than one 17 (13), 3573. doi: 10.7150/ijbs.60551 PMC8416725 34512166

[B183] Liang D. Wu F. Zhou D. Tan B. Chen T. J.C. r. i. f. s.. (2024). Commercial probiotic products in public health: Current status and potential limitations 64 (19), 6455–6476. doi: 10.1080/10408398.2023.2169858 36688290

[B184] Lin X. Hung A. Singleton W. Darmawan K. K. Moses R. Yao B.. (. 2020). Advances in delivery systems for the therapeutic application of LL37 60, 102016. doi: 10.1016/j.foodchem.2020.126789

[B185] Lin D. M. Koskella B. Lin H. C.J.W. j. o. g. p.. (2017). Phage therapy: An alternative to antibiotics in the age of multi-drug resistance 8 (3), 162. doi: 10.4292/wjgpt.v8.i3.162 PMC5547374 28828194

[B186] Lin B. Hung A. Singleton W. Darmawan K. K. Moses R. Yao B.. (2023). The effect of tailing lipidation on the bioactivity of antimicrobial peptides and their aggregation tendency: Special issue: Emerging investigators 4 (4), e329. doi: 10.1002/agt2.v4.4

[B187] Liu Y. Y. Wang Y. Walsh T. R. Yi L.-X. Zhang R. Spencer J.. (2016). Emergence of plasmid-mediated colistin resistance mechanism MCR-1 in animals and human beings in China: a microbiological and molecular biological study 16 (2), 161–168. doi: 10.1016/S1473-3099(15)00424-7 26603172

[B188] Liu D. Van Belleghem J. D. de Vries C. R. Burgener E. Chen Q. Manasherob R.. (2021). The safety and toxicity of phage therapy: a review of animal and clinical studies 13 (7), 1268. doi: 10.3390/v13071268 PMC8310247 34209836

[B189] Liu Y. Zhu S. Gu Z. Chen C. Zhao Y. J.P. (2022). Toxicity of manufactured nanomaterials 69, 31–48. doi: 10.1016/j.partic.2021.11.007

[B190] Liu M. Gao H. Miao J. Zhang Z. Zheng L. Li F.. (2024). Helicobacter pylori infection in humans and phytotherapy, probiotics, and emerging therapeutic interventions: a review 14, 1330029. doi: 10.3389/fmicb.2023.1330029 PMC10806011 38268702

[B191] Liu Y. Tran D. Q. Rhoads C. P. (2018). Probiotics in disease prevention and treatment 58, S164–S179. doi: 10.1002/jcph.v58.S10 PMC6656559 30248200

[B192] Lobritz M. A. Belenky P. Porter C. B. Gutierrez A. Yang J. H. Schwarz E. G.. (2015). Antibiotic efficacy is linked to bacterial cellular respiration 112 (27), 8173–8180. doi: 10.1073/pnas.1509743112 PMC4500273 26100898

[B193] Loc-Carrillo C. Abedon J. B. (2011). Pros and cons of phage therapy 1 (2), 111–114. doi: 10.4161/bact.1.2.14590 PMC3278648 22334867

[B194] Loh B. Gondil V. S. Manohar P. Khan F. M. Yang H. Leptihn S. J.A.. (2021). Encapsulation and delivery of therapeutic phages 87 (5), e01979–e01920. doi: 10.1128/AEM.01979-20 PMC8090888 33310718

[B195] Lopatkin A. J. Bening S. C. Manson A. L. Stokes J. M. Kohanski M. A. Badran A. H.. (2021). Clinically relevant mutations in core metabolic genes confer antibiotic resistance 371 (6531), eaba0862. doi: 10.1126/science.aba0862 PMC8285040 33602825

[B196] Luo M. Jia Y.-Y. Jing Z.-W. Li C. Zhou S.-Y. Mei Q.-B.. (2018). Construction and optimization of pH-sensitive nanoparticle delivery system containing PLGA and UCCs-2 for targeted treatment of Helicobacter pylori 164, 11–19. doi: 10.1016/j.colsurfb.2018.01.008 29367052

[B197] Luo X. Chen H. Song Y. Qin Z. Xu L. He N.. (2023). Advancements, challenges and future perspectives on peptide-based drugs: Focus on antimicrobial peptides 181, 106363. doi: 10.1016/j.ejps.2022.106363 36529161

[B198] Lüthje P. von-Köckritz-Blickwede M. Schwarz A. C. (2007). Identification and characterization of nine novel types of small staphylococcal plasmids carrying the lincosamide nucleotidyltransferase gene lnu (A) 59 (4), 600–606. doi: 10.1128/AAC.00234-07 17329268

[B199] Ma L. Liu X. Liang H. Che Y. Chen C. Dai H.. (2012). Effects of 14-alpha-lipoyl andrographolide on quorum sensing in Pseudomonas aeruginosa. Antimicrobial Agents chemotherapy 56 (12), 6088–6094. doi: 10.1128/AAC.01119-12 22802260 PMC3497202

[B200] Ma F. Xu S. Tang Z. Li Z. Zhang L. J.B.. (2021). Use of antimicrobials in food animals and impact of transmission of antimicrobial resistance on humans 3, 32–38. doi: 10.1016/j.bsheal.2020.09.004

[B201] Ma Q. Wang G. Li N. Wang X. Kang X. Mao Y.. (2023). Insights into the effects and mechanism of andrographolide-mediated recovery of susceptibility of methicillin-resistant Staphylococcus aureus to β-Lactam Antibiotics 11 (1), e02978–e02922. doi: 10.1128/spectrum.02978-22 PMC9927479 36602386

[B202] Magana M. Pushpanathan M. Santos A. L. Leanse L. Fernandez M. Ioannidis A.. (2020). The value of antimicrobial peptides in the age of resistance 20 (9), e216–e230. doi: 10.1016/S1473-3099(20)30327-3 32653070

[B203] Makabenta J. M. V. Nabawy A. Li C.-H. Schmidt-Malan S. Patel R. Rotello V. M.J.N.R.M. (2021). Nanomaterial-based therapeutics for antibiotic-resistant bacterial infections 19 (1), 23–36. doi: 10.1038/s41579-020-0420-1 PMC8559572 32814862

[B204] Malik E. Dennison S. R. Harris F. Phoenix D. A.J.P. (2016). pH dependent antimicrobial peptides and proteins, their mechanisms of action and potential as therapeutic agents 9 (4), 67. doi: 10.3390/ph9040067 PMC5198042 27809281

[B205] Mancuso G. Midiri A. Gerace E. Biondo C. J.P. (2021). Bacterial antibiotic resistance: The most critical pathogens 10 (10), 1310. doi: 10.3390/pathogens10101310 PMC8541462 34684258

[B206] Martinez J. L. Fajardo A. Garmendia L. Hernandez A. Linares J. F. Martínez-Solano L.. (2008). A global view of antibiotic resistance 33 (1), 44–65. doi: 10.1111/j.1574-6976.2008.00142.x 19054120

[B207] Martins A. F. Rabinowitz F. M. (2020). The impact of antimicrobial 89iiiiiiiiiresistance in the environment on public health. Future Med. 15, 699–702. doi: 10.1016/j.jchromb.2020.122134 32530297

[B208] Masadeh M. M. Karasneh G. A. Al-Akhras M. A. Albiss B. A. Aljarah K. M. Al-Azzam S. I.. (2015). Cerium oxide and iron oxide nanoparticles abolish the antibacterial activity of ciprofloxacin against gram positive and gram negative biofilm bacteria 67, 427–435. doi: 10.1007/s10616-014-9701-8 PMC4371563 24643389

[B209] Matheson J. A. T. Holsinger M. S. (2023). The role of fecal microbiota transplantation in the treatment of neurodegenerative diseases: a review 24 (2), 1001. doi: 10.3390/ijms24021001 PMC9864694 36674517

[B210] Meile S. Du J. Dunne M. Kilcher S. Loessner M. J. J. C. o. i. v. (2022). Engineering therapeutic phages for enhanced antibacterial efficacy 52, 182–191. doi: 10.1016/j.coviro.2021.12.003 34952266

[B211] Melo L. D. Oliveira H. Pires D. P. Dabrowska K. Azeredo J. J.C. r. i. m. (2020). Phage therapy efficacy: a review of the last 10 years of preclinical studies 46 (1), 78–99. doi: 10.1080/1040841X.2020.1729695 32091280

[B212] Memariani M. Memariani R. (2024). Therapeutics, Anti-biofilm effects of melittin: lessons learned and the path ahead 30 (3), 1–19. doi: 10.1007/s10989-024-10606-w

[B213] Meneses L. Brandao A. C. Coenye T. Braga A. C. Pires D. P. Azeredo J. J. E. o. J. C. M.. (2023). A systematic review of the use of bacteriophages for *in vitro* biofilm control 42 (8), 919–928. doi: 10.1007/s10096-023-04638-1 PMC10345070 37407800

[B214] Merli P. Putignani L. Ruggeri A. Del Chierico F. Gargiullo L. Galaverna F.. (2020). Decolonization of multi-drug resistant bacteria by fecal microbiota transplantation in five pediatric patients before allogeneic hematopoietic stem cell transplantation: gut microbiota profiling, infectious and clinical outcomes 105 (11), 2686. doi: 10.1016/j.foodres.2020.109123 PMC7604639 33131263

[B215] Miernicki M. Hofmann T. Eisenberger I. von der Kammer F. Praetorius A. J.N. n. (2019). Legal and practical challenges in classifying nanomaterials according to regulatory definitions. 14 (3), 208–216. doi: 10.1038/s41565-019-0396-z 30837754

[B216] Miernikiewicz P. Barylski J. Wilczak A. Dragoš A. Rybicka I. Bałdysz S.. (. 2023). New phage-derived antibacterial enzyme polaR targeting rothia spp 12 (15), 1997. 10.3390/cells12151997 PMC10417112 37566076

[B217] Millan B. Park H. Hotte N. Mathieu O. Burguiere P. Tompkins T. A.. (2016). Fecal microbial transplants reduce antibiotic-resistant genes in patients with recurrent Clostridium difficile infection 62 (12), 1479–1486. doi: 10.1093/cid/ciw185 PMC4885654 27025836

[B218] Mohanraj R. (2017). “Antimicrobial activities of metallic and metal oxide nanoparticles from plant extracts,” in Antimicrobial nanoarchitectonics (Chennai, India: Elsevier), 83–100.

[B219] Mojica M. F. Rossi M.-A. Vila A. J. Bonomo R. A.J.T.L.I.D. (2022). The urgent need for metallo-β-lactamase inhibitors: an unattended global threat 22 (1), e28–e34. doi: 10.1016/S1473-3099(20)30868-9 PMC8266270 34246322

[B220] Muaz K. Riaz M. Akhtar S. Park S. Ismail A. J.J. o. f. p. (2018). Antibiotic residues in chicken meat: global prevalence, threats, and decontamination strategies: a review 81 (4), 619–627. doi: 10.4315/0362-028X.JFP-17-086 29537307

[B221] Muthu M. S. Wilson J. N. (2012). Challenges posed by the scale-up of nanomedicines (Singapore: Taylor & Francis), 307–309. 10.2217/nnm.12.3 22385192

[B222] Mutuku C. Gazdag Z. Melegh S. J.W.J.M.. (2022). Occurrence of antibiotics and bacterial resistance genes in wastewater: resistance mechanisms and antimicrobial resistance control approaches 38 (9), 152. doi: 10.1007/s11274-022-03334-0 PMC9250919 35781751

[B223] Nagy A. Harrison A. Sabbani S. Munson R. S. Jr Dutta P. K. Waldman W. J.J.I. j. o. n. (2011). Silver nanoparticles embedded in zeolite membranes: release of silver ions and mechanism of antibacterial action. Clinical Microbiology Reviews. 24, 1833–1852. doi: 10.2147/IJN.S24019 PMC3173047 21931480

[B224] Nakai K. Tsuruta S. (2021). What are reactive oxygen species, free radicals, and oxidative stress in skin diseases? 22 (19), 10799. doi: 10.3390/ijms221910799 PMC8509443 34639139

[B225] Negrescu A. M. Killian M. S. Raghu S. N. Schmuki P. Mazare A. Cimpean A. J.J. o. F.B. (. 2022). Metal oxide nanoparticles: review of synthesis, characterization and biological effects 13 (4), 274. doi: 10.3390/jfb13040274 PMC9780975 36547533

[B226] Nilsson A. S. (2014). Phage therapy—constraints and possibilities. Upsala J. Med. Sci. 119 (2), 192–198. doi: 10.3109/03009734.2014.902878 24678769 PMC4034558

[B227] O’Neill J. (2016). Tackling drug-resistant infections globally: final report and recommendations. (London, UK: HM Government (UK Government Publishing)).

[B228] Opal S. M. (2016). Non-antibiotic treatments for bacterial diseases in an era of progressive antibiotic resistance (Providence, RI, USA: Springer), 1–3. 10.1186/s13054-016-1549-1 PMC5159963 27978847

[B229] Organisation W. R. (2017). World gastroenterology organisation global guidelines-probiotics and prebiotics. (Geneva, Switzerland: World Health Organization (WHO)).

[B230] Organization, W.H (2024). WHO bacterial priority pathogens list, 2024 (Geneva, Switzerland: World Health Organization).

[B231] Ozdal M. Gurkok S. J. A. (2022). DMPK, Recent advances in nanoparticles as antibacterial agent 10 (2), 115–129. doi: 10.1016/j.plaphy.2022.01.001 PMC8957245 35350114

[B232] Page S. Gautier T. O. (2012). Use of antimicrobial agents in livestock 31 (1), 145. doi: 10.20506/rst.issue.31.1.49 22849274

[B233] Pal N. Sharma P. Kumawat M. Singh S. Verma V. Tiwari R. R.. (2024). Phage therapy: An alternative treatment modality for MDR bacterial infections 56 (10), 785–817. doi: 10.1080/23744235.2024.2379492 39017931

[B234] Pandey D. P. Gerdes K. (2005). Toxin–antitoxin loci are highly abundant in free-living but lost from host-associated prokaryotes. Nucleic Acids Res. 33 (3), 966–976. doi: 10.1093/nar/gki201 15718296 PMC549392

[B235] Park S. Y. Seo C. E. (2021). Fecal microbiota transplantation: is it safe? 54 (2), 157–160. doi: 10.5946/ce.2021.072 PMC8039753 33827154

[B236] Pawlowski A. C. Wang W. Koteva K. Barton H. A. McArthur A. G. Wright G. D.J.N. c. (2016). A diverse intrinsic antibiotic resistome from a cave bacterium 7 (1), 13803. doi: 10.1038/ncomms13803 PMC5155152 27929110

[B237] Peleg A. Y. Seifert H. Paterson M. R. (2008). Acinetobacter baumannii: emergence of a successful pathogen 21 (2), 538–582. doi: 10.1128/CMR.00058-07 PMC2493088 18625687

[B238] Peng B. Su Y.-b. Li H. Han Y. Guo C. Tian Y.-m.. (2015). Exogenous alanine and/or glucose plus kanamycin kills antibiotic-resistant bacteria 21 (2), 249–262. doi: 10.1016/j.cmet.2015.01.008 25651179

[B239] Penn A. Murphy G. Barker S. Henk W. Penn L. J.E.H.P. (2005). Combustion-derived ultrafine particles transport organic toxicants to target respiratory cells 113 (8), 956–963. doi: 10.1289/ehp.7661 PMC1280333 16079063

[B240] Penumetcha S. S. Ahluwalia S. Irfan R. Khan S. A. Reddy S. R. Lopez M. E.V.. (2021). The efficacy of probiotics in the management of Helicobacter pylori: a systematic review. Nature Communications 13 (12), 1–12. doi: 10.7759/cureus.20483 PMC8760009 35047301

[B241] Piewngam P. Zheng Y. Nguyen T. H. Dickey S. W. Joo H.-S. Villaruz A. E.. (2018). Pathogen elimination by probiotic Bacillus via signalling interference 562 (7728), 532–537. doi: 10.1038/s41586-018-0616-y PMC6202238 30305736

[B242] Pitout J. D. Peirano G. Kock M. M. Strydom K.-A. Matsumura Y. J.C. m. r. (2019). The global ascendency of OXA-48-type carbapenemases. Clinical Microbiology Reviews 33 (1), e00117-18. doi: 10.1128/cmr.00102-19 PMC6860007 31722889

[B243] Poole K. Srikumar M. C. (2001). Multidrug efflux in Pseudomonas aeruginosa components, mechanisms and clinical significance 1 (1), 59–71. doi: 10.2174/1568026013395605 11895293

[B244] Pop O. L. Mesaros A. Vodnar D. C. Suharoschi R. Tabaran F. Mageru?an L.. (2020). Cerium oxide nanoparticles and their efficient antibacterial application *in vitro* against gram-positive and gram-negative pathogens 10 (8), 1614. doi: 10.3390/nano10081614 PMC7466638 32824660

[B245] Potter R. F. D’Souza A. W. Dantas R. U. (2016). The rapid spread of carbapenem-resistant Enterobacteriaceae 29, 30–46. doi: 10.1016/j.drup.2016.09.002 PMC5140036 27912842

[B246] Prajapati J. D. Kleinekathofer U. Winterhalter C. R. (2021). How to enter a bacterium: bacterial porins and the permeation of antibiotics 121 (9), 5158–5192. doi: 10.1021/acs.chemrev.0c01213 33724823

[B247] Puiu R. A. Balaure P. C. Constantinescu E. Grumezescu A. M. Andronescu E. Oprea O.-C.. (2021). Anti-cancer nanopowders and MAPLE-fabricated thin films based on SPIONs surface modified with paclitaxel loaded β-cyclodextrin 13 (9), . 1356. doi: 10.1016/j.foodchem.2021.129876 PMC8468465 34575432

[B248] Qiu J. Zhai Y. Wei M. Zheng C. Jiao X. J.M.R. (2022). Toxin–antitoxin systems: classification, biological roles, and applications 264, 127159. doi: 10.1016/j.micres.2022.127159 35969944

[B249] Rafiq S. Hao H. Ijaz M. Raza A. J.P. (2022). Pharmacological effects of Houttuynia cordata Thunb (H. cordata): a comprehensive review 15 (9), 1079. doi: 10.1016/j.lwt.2022.108765 PMC9501394 36145299

[B250] Raheem A. Liang L. Zhang G. Cui S. J.F. i. i. (2021). Modulatory effects of probiotics during pathogenic infections with emphasis on immune regulation 12, 616713. doi: 10.3389/fimmu.2021.616713 PMC8060567 33897683

[B251] Rai S. Kumar M. R. (2022). Bacteriophage therapeutics to confront multidrug-resistant Acinetobacter baumannii-a global health menace 14 (3), 347–364. doi: 10.1111/1758-2229.12988 34196126

[B252] Ramirez M. S. Tolmasky R. U. (2010). Aminoglycoside modifying enzymes 13 (6), 151–171. doi: 10.1016/j.drup.2010.08.003 PMC2992599 20833577

[B253] Ramirez-Sanchez C. Gonzales F. Buckley M. Biswas B. Henry M. Deschenes M. V.. (2021). Successful treatment of Staphylococcus aureus prosthetic joint infection with bacteriophage therapy 13 (6), 1182. doi: 10.3390/v13061182 PMC8233819 34205687

[B254] Rasmussen K. Sokull-Klüttgen B. Yu I. J. Kanno J. Hirose A. Gwinn M. R. (2017). “Regulation and legislation,” in Adverse effects of engineered nanomaterials (Copenhagen, Denmark: Elsevier), 159–188.

[B255] Rauscher H. Rasmussen K. Sokull-Klüttgen T. (2017). Regulatory aspects of nanomaterials in the EU 89 (3), 224–231. doi: 10.1002/cite.201600076

[B256] Ravishankar T. N. Ramakrishnappa T. Nagaraju G. Rajanaika H. J.C. (. 2015). Synthesis and characterization of CeO2 nanoparticles via solution combustion method for photocatalytic and antibacterial activity studies 4 (2), 146–154. doi: 10.1016/j.foodcont.2015.03.001 PMC4420586 25969812

[B257] Reddy Yadav L. Manjunath K. Archana B. Madhu C. Raja Naika H. Nagabhushana H.. (2016). Fruit juice extract mediated synthesis of CeO 2 nanoparticles for antibacterial and photocatalytic activities 131, 1–10. doi: 10.1140/epjp/i2016-16154-y

[B258] Równicki M. Lasek R. Trylska J. Bartosik D. (2020). Targeting type II toxin-antitoxin systems as antibacterial strategies. . Toxins (Basel) 12 (9), 123–135. doi: 10.1016/j.foodchem.2020.126789 32899634 PMC7551001

[B259] Ryman-Rasmussen J. P. Riviere J. E. Monteiro-Riviere T. S. (2006). Penetration of intact skin by quantum dots with diverse physicochemical properties 91 (1), 159–165. doi: 10.1093/toxsci/kfj122 16443688

[B260] Sadelaji S. Ghaznavi-Rad E. Abbasian S. S. Fahimirad S. Abtahi H. J. I. J. o. B. M. S. (2022). Ib-AMP4 antimicrobial peptide as a treatment for skin and systematic infection of methicillin-resistant Staphylococcus aureus (MRSA) 25 (2), 232. doi: 10.1016/j.foodres.2022.110123 PMC9124539 35655604

[B261] Sakowicz C. M. Lojewska E. (2021a). An alternative to antibiotics: selected methods to combat zoonotic foodborne bacterial infections. Polymers. 13, 1–13. doi: 10.1016/j.polymdegradstab.2021.109567 PMC8595143 34626217

[B262] Sánchez-López E.. (2020). Metal-based nanoparticles as antimicrobial agents: an overview 10 (2), 292. doi: 10.3390/nano10020292 PMC7075170 32050443

[B263] Sani M. A. Separovic F. (2016). How membrane-active peptides get into lipid membranes. Accounts Chem. Res. 49 (6), 1130–1138. doi: 10.1021/acs.accounts.6b00074 27187572

[B264] Sassone-Corsi M. Raffatellu O. I. (2015). No vacancy: how beneficial microbes cooperate with immunity to provide colonization resistance to pathogens 194 (9), 4081–4087. doi: 10.4049/jimmunol.1403169 PMC4402713 25888704

[B265] Sato Y. Ubagai T. Tansho-Nagakawa S. Yoshino Y. Ono Y. J.S.R. (2021). Effects of colistin and tigecycline on multidrug-resistant Acinetobacter baumannii biofilms: advantages and disadvantages of their combination 11 (1), 11700. doi: 10.1038/s41598-021-90732-3 PMC8175759 34083569

[B266] Schaenzer A. J. Wright M. M. (2020). Antibiotic resistance by enzymatic modification of antibiotic targets 26 (8), 768–782. doi: 10.1016/j.molmed.2020.05.001 32493628

[B267] Scher J. U. Sczesnak A. Longman R. S. Segata N. Ubeda C. Bielski C.. (2013). Expansion of intestinal Prevotella copri correlates with enhanced susceptibility to arthritis. elife 2, e01202. doi: 10.7554/eLife.01202.028 24192039 PMC3816614

[B268] Schrader S. M. Vaubourgeix J. Nathan T. M. (2020). Biology of Tantimicrobial resistance and approaches to combat it 12 (549), eaaz6992. doi: 10.1126/scitranslmed.aaz6992 PMC8177555 32581135

[B269] Schwarz S. Werckenthin C. Kehrenberg C. J.A. a.. (2000). Identification of a plasmid-borne chloramphenicol-florfenicol resistance gene in Staphylococcus sciuri 44 (9), 2530–2533. doi: 10.1128/AAC.44.9.2530-2533.2000 PMC90098 10952608

[B270] Setlow J. Randesi M. Adams J. Setlow B. Setlow P. (1992). Mutation and killing of Escherichia coli expressing a cloned Bacillus subtilis gene whose product alters DNA conformation. J. bacteriology 174 (9), 2943–2950. doi: 10.1128/jb.174.9.2943-2950.1992 PMC205948 1314805

[B271] Shakil M. S. Bhuiya M. S. Morshed M. R. Babu G. Niloy M. S. Hossen M. S.. (2023). Cobalt ferrite nanoparticle’s safety in biomedical and agricultural applications: a review of recent progress 30 (15), 1756–1775. doi: 10.2174/0929867329666221007113951 36214302

[B272] Shokri D. Soleimani-Delfan A. Fatemi C. P. (2017). Assessment of phage cocktails with extended host range activity against antibiotic resistant strains of Pseudomonas aeruginosa 26, 417–422. doi: 10.1007/s00580-016-2394-y

[B273] Shoudho K. N. Uddin S. Rumon M. M.H. Shakil M. S. J. A. o. (2024). Influence of Physicochemical properties of Iron Oxide nanoparticles on their antibacterial activity 9 (31), 33303–33334. doi: 10.1021/acsomega.4c02822 PMC11308002 39130596

[B274] Si Z. Pethe K. Chan-Park J. A. (2023). Chemical basis of combination therapy to combat antibiotic resistance 3 (2), 276–292. doi: 10.1021/jacsau.2c00532 PMC9975838 36873689

[B275] Silva D. R. Dalcolmo M. Tiberi S. Arbex M. A. Munoz-Torrico M. Duarte R.. (2018). New and repurposed drugs to treat multidrug-and extensively drug-resistant tuberculosis 44, 153–160. doi: 10.1590/s1806-37562017000000436 PMC6044661 29791557

[B276] Simoneit C. Burow E. Tenhagen B.-A. Käsbohrer A. J.P. v. m. (2015). Oral administration of antimicrobials increase antimicrobial resistance in E. coli from chicken–a systematic review 118 (1), 1–7. doi: 10.1016/j.orggeochem.2015.01.001 25433717

[B277] Singh A. Amod A. Pandey P. Bose P. Pingali M. S. Shivalkar S.. (2022). Bacterial biofilm infections, their resistance to antibiotics therapy and current treatment strategies 17 (2), 022003. doi: 10.1088/1748-605X/ac50f6 35105823

[B278] Sivaraman S. Yann A. P. (2018). Antibiotic use in food animals: India overview. Frontiers in Microbiology. 9 (1), 1–31. doi: 10.1016/j.foodchem.2018.03.001 29403456 PMC5778136

[B279] Slavin Y. N. Asnis J. Häfeli U. O. Bach H. (2017). Metal nanoparticles: understanding the mechanisms behind antibacterial activity. J. nanobiotechnology 15 (1), 1–20. doi: 10.1186/s12951-017-0308-z 28974225 PMC5627441

[B280] Smith W. P. Wucher B. R. Nadell C. D. Foster K. R.J.N.R.M. (2023). Bacterial defences: mechanisms, evolution and antimicrobial resistance 21 (8), 519–534. doi: 10.1038/s41579-023-00877-3 37095190

[B281] Smits L. P. Bouter K. E. De Vos W. M. Borody T. J. Nieuwdorp M. J.G. (2013). Therapeutic potential of fecal microbiota transplantation 145 (5), 946–953. doi: 10.1053/j.gastro.2013.08.058 24018052

[B282] Soltani A. M. Pouypouy H. (2019). Standardization and regulations of nanotechnology and recent government policies across the world on nanomaterials, in Advances in phytonanotechnology (Tehran, Iran: Elsevier), 419–446.

[B283] Sonika S. Singh S. Mishra S. Verma S. J.H. (2023). Toxin-antitoxin systems in bacterial pathogenesis. Scientific Reports 9, 1–10. doi: 10.1016/j.heliyon.2023.e14220 PMC10123168 37101643

[B284] Sousa C. Ferreira R. Azevedo N. F. Oleastro M. Azeredo J. Figueiredo C.. (2022). Helicobacter pylori infection: from standard to alternative treatment strategies 48 (3), 376–396. doi: 10.1080/1040841X.2021.1975643 34569892

[B285] Spellberg B. (2009). Rising plague: the global threat from deadly bacteria and our dwindling arsenal to fight them (Washington, D.C., USA: Prometheus Books).

[B286] Ssekatawa K. Byarugaba D. K. Kato C. D. Wampande E. M. Ejobi F. Tweyongyere R.. (2021). A review of phage mediated antibacterial applications 57 (1), 1–20. doi: 10.1080/20905068.2020.1851441

[B287] Stapleton A. E. Au-Yeung M. Hooton T. M. Fredricks D. N. Roberts P. L. Czaja C. A.. (2011). Randomized, placebo-controlled phase 2 trial of a Lactobacillus crispatus probiotic given intravaginally for prevention of recurrent urinary tract infection 52 (10), 1212–1217. doi: 10.1093/cid/cir183 PMC3079401 21498386

[B288] Stapleton P. D. Taylor S. P. (2002). Methicillin resistance in Staphylococcus aureus: mechanisms and modulation 85 (1), 57–72. doi: 10.3184/003685002783238870 PMC2065735 11969119

[B289] Stickland H. G. Davenport P. W. Lilley K. S. Griffin J. L. Welch M. J.J. o. p. r. (2010). Mutation of nfxB causes global changes in the physiology and metabolism of Pseudomonas aeruginosa 9 (6), 2957–2967. doi: 10.1021/pr9011415 20373734

[B290] Stokes J. M. Lopatkin A. J. Lobritz M. A. Collins J. J.J.C. m. (2019). Bacterial metabolism and antibiotic efficacy 30 (2), 251–259. doi: 10.1016/j.cmet.2019.06.009 PMC6990394 31279676

[B291] Su G. Zhang X. Giesy J. P. Musarrat J. Saquib Q. Alkhedhairy A. A.. (2015). Comparison on the molecular response profiles between nano zinc oxide (ZnO) particles and free zinc ion using a genome-wide toxicogenomics approach. 22, 17434–17442. doi: 10.1007/s11356-015-4507-6 25940466

[B292] Su P. W. Yang C.-H. Yang J.-F. Su P.-Y. Chuang L.-Y. J.M. (2015). Antibacterial activities and antibacterial mechanism of Polygonum cuspidatum extracts against nosocomial drug-resistant pathogens 20 (6), 11119–11130. doi: 10.3390/molecules200611119 PMC6272736 26087259

[B293] Suez J. Zmora N. Zilberman-Schapira G. Mor U. Dori-Bachash M. Bashiardes S.. (2018). Post-antibiotic gut mucosal microbiome reconstitution is impaired by probiotics and improved by autologous FMT 174 (6), 1406–1423. e16. doi: 10.1016/j.cell.2018.05.001 30193113

[B294] Summers W. C. (2021). Technology, therapy, The discovery of bacteriophages and the historical context p, 387–400. doi: 10.1007/978-3-319-41986-2

[B295] Sumrall E. T. Shen Y. Keller A. P. Rismondo J. Pavlou M. Eugster M. R.. (2019). Phage resistance at the cost of virulence: Listeria monocytogenes serovar 4b requires galactosylated teichoic acids for InlB-mediated invasion 15 (10), e1008032. doi: 10.1371/journal.ppat.1008032 PMC6779246 31589660

[B296] Sun J. Xia Y. Li D. Du Q. Liang D. J.B.e.B.A.-B. (. 2014). Relationship between peptide structure and antimicrobial activity as studied by de novo designed peptides 1838 (12), 2985–2993. doi: 10.1016/j.foodchem.2014.01.001 25157672

[B297] Sun X. Zhang S. Ren J. Udenigwe C. C.J.C.R. i. F.S. (2022). Sialic acid-based strategies for the prevention and treatment of Helicobacter pylori infection: emerging trends in food industry 62 (7), 1713–1724. doi: 10.1080/10408398.2020.1846157 33207917

[B298] Surendra T. Roopan O. P. Biology P. B. (2016). Photocatalytic and antibacterial properties of phytosynthesized CeO2 NPs using Moringa oleifera peel extract 161, 122–128. doi: 10.1016/j.jphotobiol.2016.05.019 27236047

[B299] Swoboda J. G. Campbell J. Meredith T. C. Walker S. J.C.a.E.j.o.c.b. (2010). Wall teichoic acid function, biosynthesis, and inhibition 11 (1), 35. doi: 10.1002/cbic.200900557 PMC2798926 19899094

[B300] Syed B. Bisht N. Bhat P. S. Prasad A. Dhananjaya B. Satish S.. (2017). Phytogenic synthesis of nanoparticles from Rhizophora mangle and their bactericidal potential with DNA damage activity. Nano-Structures Nano-Objects 10, 112–115. doi: 10.1016/j.nanoso.2017.03.011

[B301] Syed B. Prasad M. N. Satish S. (2019). Synthesis and characterization of silver nanobactericides produced by Aneurinibacillus migulanus 141, a novel endophyte inhabiting Mimosa pudica L. Arabian J. Chem. 12 (8), 3743–3752. doi: 10.1016/j.arabjc.2016.01.005

[B302] Symmons M. F. Bokma E. Koronakis E. Hughes C. Koronakis V. J. P. O. t. N. A. o. S. (2009). The assembled structure of a complete tripartite bacterial multidrug efflux pump 106 (17), 7173–7178. doi: 10.1073/pnas.0900693106 PMC2678420 19342493

[B303] Taati Moghadam M. Khoshbayan A. Chegini Z. Farahani I. Shariati A. J.D.. (2020). Bacteriophages, a new therapeutic solution for inhibiting multidrug-resistant bacteria causing wound infection: lesson from animal models and clinical trials. Scientific Reports. 10, 1867–1883. doi: 10.2147/DDDT.S251171 32523333 PMC7237115

[B304] Tagliaferri T. L. Jansen M. Horz H.-P. J.F.I.c. (2019). Fighting pathogenic bacteria on two fronts: phages and antibiotics as combined strategy 9, 22. doi: 10.3389/fcimb.2019.00022 PMC6387922 30834237

[B305] Taha O. A. Connerton P. L. Connerton I. F. El-Shibiny A. J.F.i.m. (2018). Bacteriophage ZCKP1: a potential treatment for Klebsiella pneumoniae isolated from diabetic foot patients 9, 317049. doi: 10.3389/fmicb.2018.02127 PMC6141743 30254618

[B306] Takemura-Uchiyama I. Uchiyama J. Osanai M. Morimoto N. Asagiri T. Ujihara T.. (2014). Experimental phage therapy against lethal lung-derived septicemia caused by Staphylococcus aureus in mice 16 (6), 512–517. doi: 10.1016/j.micinf.2014.02.011 24631574

[B307] Taverniti V. Guglielmetti S. (2011). The immunomodulatory properties of probiotic microorganisms beyond their viability (ghost probiotics: proposal of paraprobiotic concept). Genes Nutr. 6 (3), 261–274. doi: 10.1007/s12263-011-0218-x 21499799 PMC3145061

[B308] Teng C. Wang Z. Yan R. (2016). Fine particle-induced birth defects: Impacts of size, payload, and beyond 108 (3), 196–206. doi: 10.1002/bdrc.v108.3 27581067

[B309] Tenover F. C. J. (2006). Mechanisms of antimicrobial resistance in bacteria 119 (6), S3–S10. doi: 10.1016/j.amjmed.2006.03.011 16735149

[B310] Teymouri S. Yousefi M. H. Heidari S. Farokhi S. Afkhami H. Kashfi M. (2024). Beyond antibiotics: mesenchymal stem cells and bacteriophages-new approaches to combat bacterial resistance in wound infections. Mol. Biol. Rep. 52 (1), 64. doi: 10.1007/s11033-024-10163-x 39699690

[B311] Thanush D. Venkatesh P. M. (2023). Fecal microbiota transplantation: History, procedure and regulatory considerations. Antibiotics. 12, 104204. doi: 10.1016/j.lpm.2023.104204 37944641

[B312] Thill A. Zeyons O. Spalla O. Chauvat F. Rose J. Auffan M.. (2006). Cytotoxicity of CeO2 nanoparticles for Escherichia coli. Physico-chemical insight of the cytotoxicity mechanism 40 (19), 6151–6156. doi: 10.1016/j.carbpol.2006.03.001 17051814

[B313] Thorpe C. M. Kane A. V. Chang J. Tai A. Vickers R. J. Snydman D. R.J.P.o. (2018). Enhanced preservation of the human intestinal microbiota by ridinilazole, a novel Clostridium difficile-targeting antibacterial, compared to vancomycin 13 (8). e0199810. doi: 10.1371/journal.pone.0199810 PMC6071993 30071046

[B314] Toh S. M. Xiong L. Arias C. A. Villegas M. V. Lolans K. Quinn J.. (. 2007). Acquisition of a natural resistance gene renders a clinical strain of methicillin-resistant Staphylococcus aureus resistant to the synthetic antibiotic linezolid 64 (6), 1506–1514. doi: 10.1016/j.jmb.2007.03.001 PMC2711439 17555436

[B315] Tooke C. L. Hinchliffe P. Bragginton E. C. Colenso C. K. Hirvonen V. H. Takebayashi Y.. (2019). [amp]]beta;-lactamases and β-lactamase inhibitors in the 21st century 431 (18), 3472–3500. doi: 10.1016/j.jmb.2019.04.002 PMC6723624 30959050

[B316] Tsai Y. K. Fung C.-P. Lin J.-C. Chen J.-H. Chang F.-Y. Chen T.-L.. (2011). Klebsiella pneumoniae outer membrane porins OmpK35 and OmpK36 play roles in both antimicrobial resistance and virulence 55 (4), 1485–1493. doi: 10.1128/AAC.01275-10 PMC3067157 21282452

[B317] Vacca F. Sala C. Rappuoli B. (2022). Monoclonal antibodies for bacterial pathogens: Mechanisms of action and engineering approaches for enhanced effector functions 10 (9), 2126. doi: 10.3390/biomedicines10092126 PMC9496014 36140226

[B318] Vallhov H. Qin J. Johansson S. M. Ahlborg N. Muhammed M. A. Scheynius A.. (2006). The importance of an endotoxin-free environment during the production of nanoparticles used in medical applications 6 (8), 1682–1686. doi: 10.1021/nl060860z 16895356

[B319] Vaou N. Stavropoulou E. Voidarou C. Tsigalou C. Bezirtzoglou E. J.M. (2021). Towards advances in medicinal plant antimicrobial activity: A review study on challenges and future perspectives 9 (10), 2041. doi: 10.3390/microorganisms9102041 PMC8541629 34683362

[B320] Viazis N. Argyriou K. Kotzampassi K. Christodoulou D. K. Apostolopoulos P. Georgopoulos S. D.. (2022). A four-probiotics regimen combined with a standard Helicobacter pylori-eradication treatment reduces side effects and increases eradication rates 14 (3), 632. doi: 10.3390/nu14030632 PMC8838490 35276991

[B321] Vieira A. T. Rocha V. M. Tavares L. Garcia C. C. Teixeira M. M. Oliveira S. C.. (2016). Control of Klebsiella pneumoniae pulmonary infection and immunomodulation by oral treatment with the commensal probiotic Bifidobacterium longum 51A 18 (3), 180–189. doi: 10.1016/j.micinf.2015.10.008 26548605

[B322] Vincent I. M. Ehmann D. E. Mills S. D. Perros M. Barrett M. P.J.A. a.. (2016). Untargeted metabolomics to ascertain antibiotic modes of action 60 (4), 2281–2291. doi: 10.1128/AAC.02109-15 PMC4808186 26833150

[B323] Walia K. Kuo C.-F. Yeh C.-M. Chen J.-R. Cheng M.-F. Hung C.-H. J.I.. (2019). Establishing antimicrobial resistance surveillance & research network in India: journey so far 149 (2), 164–179. doi: 10.4103/ijmr.IJMR_226_18 PMC6563732 31219080

[B324] Wang J. L. Zhang J. Wei J. Jiang L. Jiang L. Sun Y.. (2018). Efficacy of φkm18p phage therapy in a murine model of extensively drug-resistant Acinetobacter baumannii infection p, 2301–2310. doi: 10.2147/IDR.S179701 PMC6245353 30532563

[B325] Wang T. Xu K. Zhao L. Tong R. Xiong L. Shi J. J.E. j. o. m. c.. (2021). Recent research and development of NDM-1 inhibitors 223, 113667. doi: 10.1016/j.ejmech.2021.113667 34225181

[B326] Wang M. Aarestrup F. M. Jensen L. B. Hammerum A. M. Bager F. J.E. i. d. (2024). Phage-inspired strategies to combat antibacterial resistance 50 (2), 196–211. doi: 10.1080/1040841X.2023.2181056 38400715

[B327] Webber M. A. Piddock A. C. (2003). The importance of efflux pumps in bacterial antibiotic resistance 51 (1), 9–11. doi: 10.1093/jac/dkg050 12493781

[B328] Wegener H. C. Aarestrup F. M. Jensen L. B. Hammerum A. M. Bager F. J.E. i. d. (1999). Use of antimicrobial growth promoters in food animals and Enterococcus faecium resistance to therapeutic antimicrobial drugs in Europe 5 (3), 329. doi: 10.3201/eid0503.990303 PMC2640785 10341169

[B329] Weisblum B. J. (1995). chemotherapy, Erythromycin resistance by ribosome modification 39 (3), 577–585. doi: 10.1128/AAC.39.3.577 PMC162587 7793855

[B330] Wieërs G. Belkhir L. Enaud R. Leclercq S. Philippart de Foy J.-M. Dequenne I.. (2020). How probiotics affect the microbiota. Front. Cell. infection Microbiol. 8, 454 doi: 10.3389/fcimb.2019.00454 PMC6974441 32010640

[B331] Wright G. D. (1999). Aminoglycoside-modifying enzymes 2 (5), 499–503. doi: 10.1016/S1369-5274(99)00007-7 10508725

[B332] Wright G. D. J. (2005). Bacterial resistance to antibiotics: enzymatic degradation and modification 57, 1451–1470. doi: 10.1016/j.addr.2005.04.002 15950313

[B333] Wu Z. Deng X. Hu Q. Xiao X. Jiang J. Ma X.. (2021). Houttuynia cordata Thunb: an ethnopharmacological review 12, 714694. doi: 10.3389/fphar.2021.714694 PMC8440972 34539401

[B334] Xia T. Kovochich M. Brant J. Hotze M. Sempf J. Oberley T.. (2006). Comparison of the abilities of ambient and manufactured nanoparticles to induce cellular toxicity according to an oxidative stress paradigm 6 (8), 1794–1807. doi: 10.1021/nl061025k 16895376

[B335] Xiao G. Li J. Sun M. S. (2023). The combination of antibiotic and non-antibiotic compounds improves antibiotic efficacy against multidrug-resistant bacteria 24 (20), 15493. doi: 10.3390/ijms242015493 PMC10607837 37895172

[B336] Yamaguchi Y. Inouye M. (2011). Regulation of growth and death in Escherichia coli by toxin–antitoxin systems 9 (11), 779–790. doi: 10.1038/nrmicro2651 21927020

[B337] Yang J. H. Wright S. N. Hamblin M. McCloskey D. Alcantar M. A. Schrübbers L.. (2019). A white-box machine learning approach for revealing antibiotic mechanisms of action 177 (6), 1649–1661.e9. doi: 10.1016/j.foodchem.2019.03.001 PMC6545570 31080069

[B338] Yang Y. Yan Y.-H. Schofield C. J. McNally A. Zong Z. Li G.-B. J.T.i.M. (2023). Metallo-β-lactamase-mediated antimicrobial resistance and progress in inhibitor discovery 31 (7), 735–748. doi: 10.1016/j.tim.2023.01.013 36858862

[B339] Yang Z. Zhou Y. Han Z. He K. Zhang Y. Wu D.. (2024). The effects of probiotics supplementation on Helicobacter pylori standard treatment: an umbrella review of systematic reviews with meta-analyses 14 (1), . 10069. doi: 10.1038/s41598-024-59399-4 PMC11066092 38697990

[B340] Yarahmadi A. Afkhami I. O. (2024). The role of microbiomes in gastrointestinal cancers: new insights 13, 1344328. doi: 10.3389/fonc.2023.1344328 PMC10867565 38361500

[B341] Yarahmadi A. Afkhami H. Javadi A. Kashfi M. J.D.. (2024a). Understanding the complex function of gut microbiota: its impact on the pathogenesis of obesity and beyond: a comprehensive review 16 (1), 308. doi: 10.1186/s13098-024-01561-z PMC11664868 39710683

[B342] Yarahmadi A. Dousti B. Karami-Khorramabadi M. Afkhami H. J.F. i. B.. (2024b). Materials based on biodegradable polymers chitosan/gelatin: a review of potential applications 12, 1397668. doi: 10.3389/fbioe.2024.1397668 PMC11327468 39157438

[B343] Ye L. Cao Z. Liu X. Cui Z. Li Z. Liang Y.. (2022). Noble metal-based nanomaterials as antibacterial agents 904, 164091. doi: 10.1016/j.jallcom.2022.164091

[B344] Yougbaré S. Mutalik C. Okoro G. Lin I.-H. Krisnawati D. I. Jazidie A.. (2021). Emerging trends in nanomaterials for antibacterial applications. Microorganisms 9, 5831–5867. doi: 10.2147/IJN.S328767 PMC8405884 34475754

[B345] Yu K. Lo J. C. Yan M. Yang X. Brooks D. E. Hancock R. E.. (2017). Anti-adhesive antimicrobial peptide coating prevents catheter associated infection in a mouse urinary infection model. Biomaterials 116, 69–81. doi: 10.1016/j.biomaterials.2016.11.047 27914268

[B346] Yu L. Shang Z. Jin Q. Chan S. Y. Hong W. Li N.. (2023). Antibody–antimicrobial conjugates for combating antibiotic resistance 12 (1), 2202207. doi: 10.1002/adhm.202202207 36300640

[B347] Yuan Y. Qu K. Tan D. Li X. Wang L. Cong C.. (2019). Isolation and characterization of a bacteriophage and its potential to disrupt multi-drug resistant Pseudomonas aeruginosa biofilms 128, 329–336. doi: 10.1016/j.micpath.2019.01.032 30682523

[B348] Zamani K. Allah-Bakhshi N. Akhavan F. Yousefi M. Golmoradi R. Ramezani M.. (2021). Antibacterial effect of cerium oxide nanoparticle against Pseudomonas aeruginosa 21, 1–11. doi: 10.1186/s12896-021-00727-1 PMC8650514 34876083

[B349] Zampieri M. Zimmermann M. Claassen M. Sauer U. J.C.. (2017). Nontargeted metabolomics reveals the multilevel response to antibiotic perturbations 19 (6), 1214–1228. doi: 10.1016/j.celrep.2017.04.002 28494870

[B350] Zhang F. Luo W. Shi Y. Fan Z. Ji G. J. O. j. o. t. A. C. o. G.. (2012). Should we standardize the 1,700-year-old fecal microbiota transplantation? 107 (11), 1755. doi: 10.1038/ajg.2012.251 23160295

[B351] Zhang M. Zhang C. Zhai X. Luo F. Du Y. Yan C. J.S.C.M. (2019). Antibacterial mechanism and activity of cerium oxide nanoparticles 62 (11), 1727–1739. doi: 10.1007/s40843-019-9471-7

[B352] Zhang L. Liang E. Cheng Y. Mahmood T. Ge F. Zhou K.. (2020). Is combined medication with natural medicine a promising therapy for bacterial biofilm infection? Biomedicine Pharmacotherapy 128, 110184. doi: 10.1016/j.biopha.2020.110184 32450528

[B353] Zhang L. Wen B. Bao M. Cheng Y. Mahmood T. Yang W.. (2021). Andrographolide sulfonate is a promising treatment to combat methicillin-resistant Staphylococcus aureus and its biofilms 12, 720685. doi: 10.3389/fphar.2021.720685 PMC8481920 34603031

[B354] Zhang H. Q. Sun C. Xu N. Liu W. J.F.I. (2024). The current landscape of the antimicrobial peptide melittin and its therapeutic potential 15, 1326033. doi: 10.3389/fimmu.2024.1326033 PMC10838977 38318188

[B355] Zhang C. Yang A. (2022). Antimicrobial peptides: from design to clinical application 11 (3), 349. doi: 10.3390/antibiotics11030349 PMC8944448 35326812

[B356] Zhao M. Zhang C. Zhai X. Luo F. Du Y. Yan C. J.S.C.M. (2024). Antibacterial effect of phage cocktails and phage-antibiotic synergy against pathogenic Klebsiella pneumoniae 9 (9), e00607–e00624. doi: 10.1128/msystems.00607-24 PMC11406915 39166877

[B357] Zhou W. Feng Y. Zong I. M. (2018). Two new lytic bacteriophages of the Myoviridae family against carbapenem-resistant Acinetobacter baumannii 9, 347622. doi: 10.3389/fmicb.2018.00850 PMC5936750 29760690

[B358] Żółkiewicz J. Kowalski B. Nowak C. Zielinski D. Adamski E. Wójcik F. (2020). Postbiotics—a step beyond pre-and probiotics 12 (8), 2189. doi: 10.1016/j.jbiotec.2020.01.001 PMC7468815 32717965

[B359] Zurawski D. V. McLendon J. A. (2020). Monoclonal antibodies as an antibacterial approach against bacterial pathogens 9 (4), 155. doi: 10.3390/antibiotics9040155 PMC7235762 32244733

